# Heterocyclic iodoniums as versatile synthons to approach diversified polycyclic heteroarenes†

**DOI:** 10.1039/c9ra07288h

**Published:** 2019-10-16

**Authors:** Daqian Zhu, Zhouming Wu, Liyun Liang, Yameng Sun, Bingling Luo, Peng Huang, Shijun Wen

**Affiliations:** State Key Laboratory of Oncology in South China, Collaborative Innovation Center for Cancer Medicine, Sun Yat-sen University Cancer Center 651 Dongfeng East Road Guangzhou 510060 China wenshj@sysucc.org.cn; School of Pharmacy, Guangdong Pharmaceutical University 280 Waihuan East Road Guangzhou 510006 China

## Abstract

Polycyclic heteroarenes are important scaffolds in the construction of pharmaceuticals. We have previously developed a series of novel heterocyclic iodoniums. In our current work, these unique iodoniums were employed to construct various complex polycyclic heteroarenes with structural diversity *via* tandem dual arylations. As a result, indole, thiophene and triphenylene motifs were fused into these heterocycles with high molecular quality, which might provide promising fragments in drug discovery. Moreover, these heterocycles could be diversified at a late stage.

## Abstract 2

The transformation of heterocyclic iodoniums led to the construction of heterocycles with a high structural diversity.

## Graphical abstract

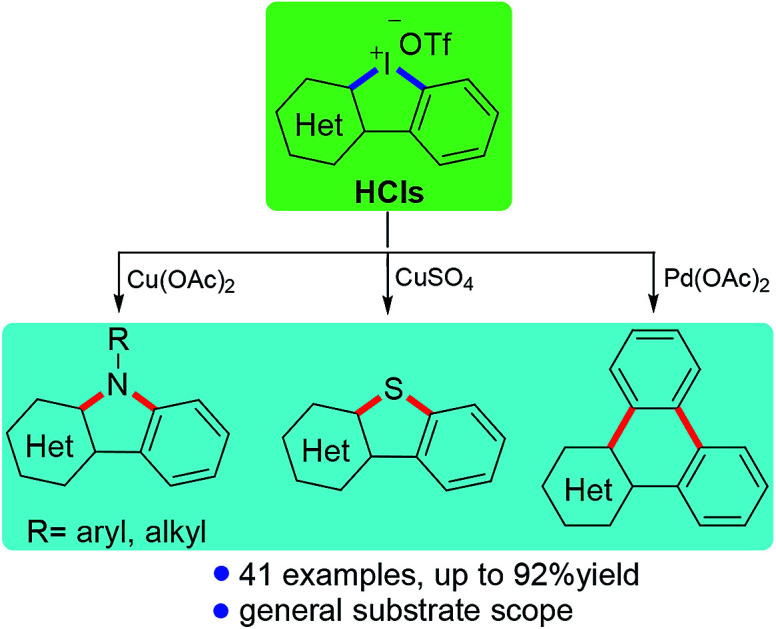

## Introduction

**Fig. 1:**
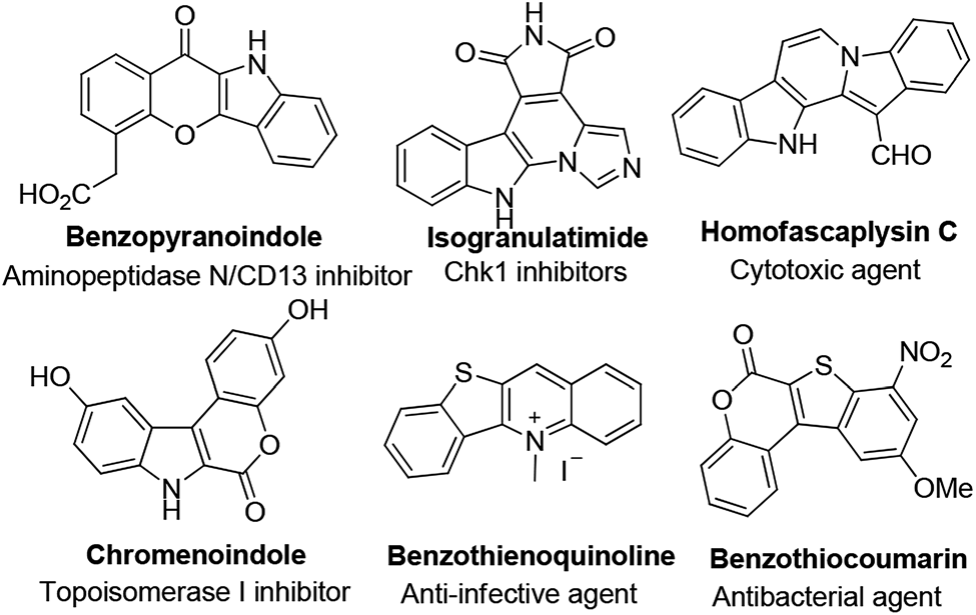
Selected examples of heterocyclic natural products and pharmaceuticals.

Polycyclic heteroarenes are important scaffolds in the construction of pharmaceuticals.^1^ Compared with polycyclic aromatic hydrocarbons, heterocycles exhibit improved solubility and bioavailability, which make them promising drug candidates.^2^ Many heterocycles are reported as kinase inhibitors, anti-infective and antibacterial agents (Fig. 1). Thus, the design and synthesis of polycyclic heteroarenes are highly demanded.

**Scheme 1:**
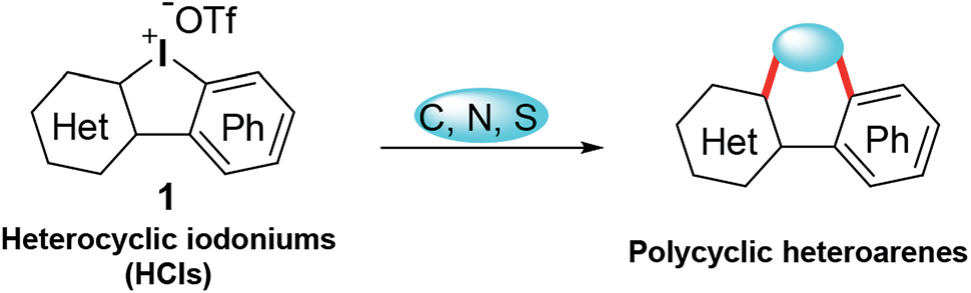
Synthesis of polycyclic heteroarenes using HCIs.

Tandem reactions enable rapid access to complex molecules, avoiding tedious purification steps and minimizing chemical waste generation.^3^ As a paradigm of tandem reactions, dual arylations with cyclic diphenyl iodoniums (CDPIs) are employed to construct various polycycles, such as acridine,^4^ carbazole,^5^ fluorene,^6^ phenanthrene,^7^ and dibenzothiophene.^8^ These obtained polycycles are often heavily hydrocarbon oriented. In the drug discovery field, heterocyclic frameworks are crucial to gain druggability. Heterocyclic iodoniums (HCIs) could be promising alternative reagents to replace CDPIs for the potential construction of heterocycles (Scheme 1). However, HCIs are under-explored and only few of them have been reported.^9^ Very recently, we have developed a series of new HCIs,^10^ and now we wish to fully investigate their synthetic application potentials to obtain diverse heterocycles *via* tandem transformations.

Indole-fused polyheterocycles are privileged structural motifs.^11^ Despite various strategies to generate these complex molecules, there still lacks a general approach to rapidly build libraries of indole-fused heterocycles with a skeleton diversity. In our previous work, dual aminations of CDPIs led to the construction of functionalized *N*-substituted carbazoles.^5*b*^ Inspired by this work, we hypothesized the amination strategy could be extended to construct indole-fused heterocycles if HCIs were used as starting materials to replace CDPIs. In this current work, we thoroughly investigated tandem dual aminations of HCIs with various amines to produce indole-fused polycyclic scaffolds. In addition, the annulations of HCIs with triethylammonium *N*-benzyldithiocarbamate and 2-chlorobenzoic acid were also fully investigated. These transformations will provide efficient pathways for rapid generation of complex heterocycles with a structural diversity.

## Results and discussion

**Scheme 2:**
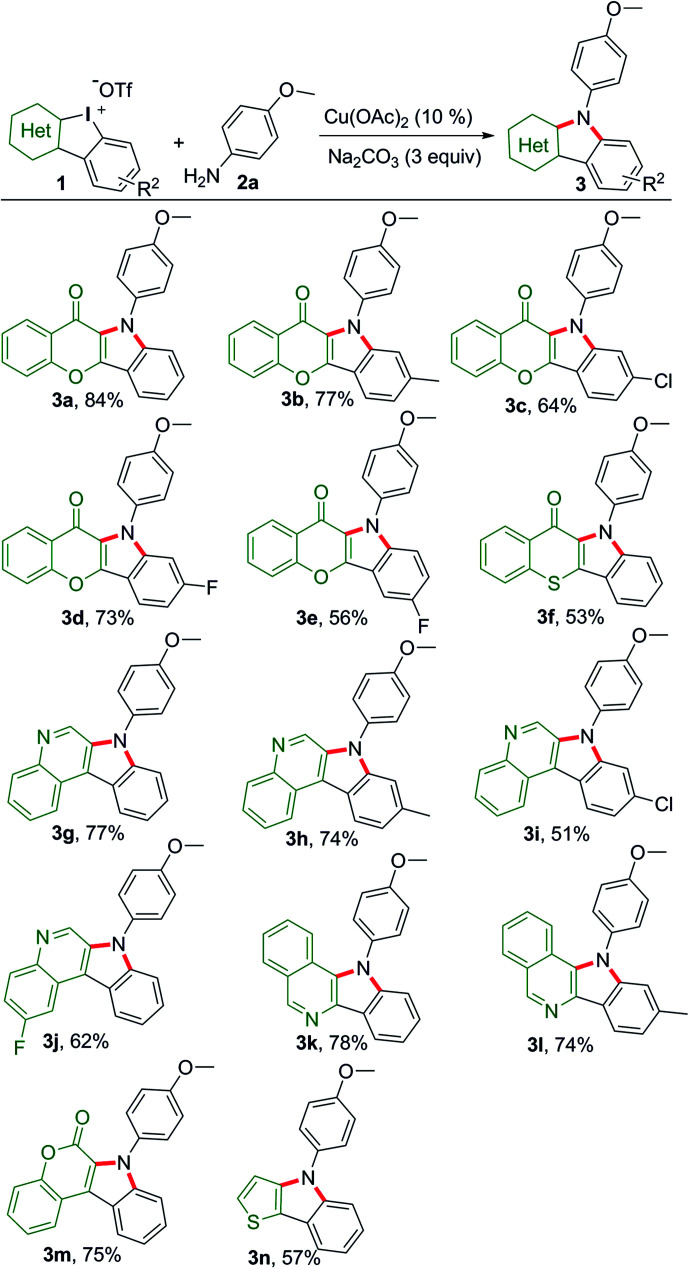
Substrate scope of HCIs to synthesize heterocycle-fused indoles. Reaction conditions: 1 (0.1 mmol), *p*-anisidine (2.5 equiv.), *i*-PrOH/(CH_2_OH)_2_ (0.9/0.1 mL), refluxing, Ar, 16 h.

Chromone is a privileged motif for drug design and discovery in the field of medicinal chemistry.^12^ Thus, we commenced the dual-amination transformation using chromone embedded HCIs 1a–1e as building blocks (Scheme 2). Under catalytic mediation of Cu(OAc)_2_, *p*-anisidine underwent dual arylations to complete the amination.

As a result, the desired chromone-fused indoles were obtained at modest to good yields (3a–3e). Then, we explored the substrate scope and generality of other HCIs with different heterocyclic motifs. Thiochromone-fused indole was obtained at a moderate yield (3f). Quinoline, isoquinoline and coumarin are important building blocks for naturally occurring products and pharmaceuticals. These unique heterocyclic fused indoles could be also assembled efficiently (3g–3m). The HCIs bearing chlorine atom usually gave low yields (3a*vs.*3c, 3g*vs.*3i). Meanwhile, the construction of thieno[3,2-*b*]indole (3n) was realized, providing a concise method to obtain thiophene-containing materials.

**Scheme 3:**
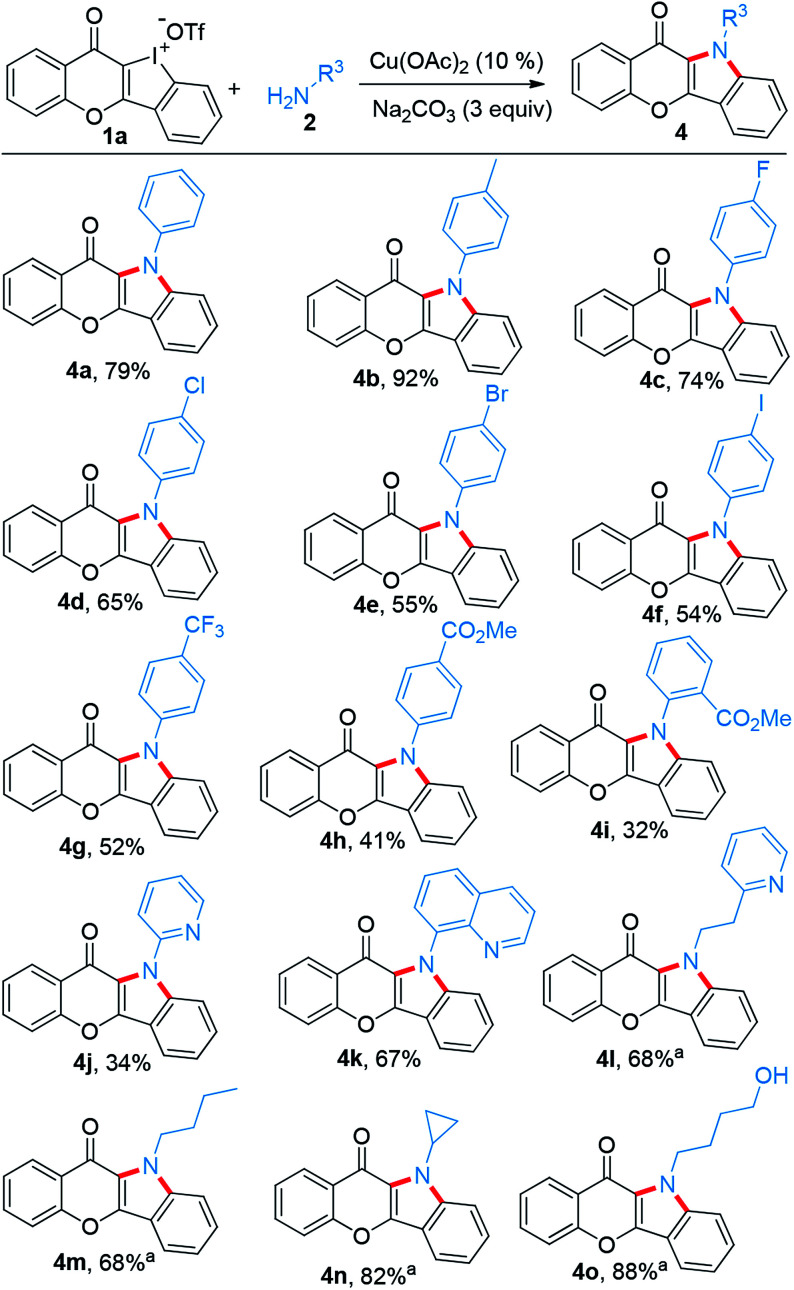
Scope of amines reacting with 1a to construct chromone-fused indoles. Reaction conditions: 1a (0.1 mmol), amine (2.5 equiv.), *i*-PrOH/(CH_2_OH)_2_ (0.9/0.1 mL), refluxing, Ar, 16 h. ^a^Additional CuI (0.1 equiv.) added.

Subsequently, the substrate scope of this reaction was further examined on variation of the amines (Scheme 3). Like *p*-anisidine, other arylamines bearing different functional groups also enabled their incorporation into chromone-containing HCI 1a to provide diverse chromone-fused indoles (4a–4i). It should be noted that the intact bromo or iodo in products 4e and 4f could serve as a potential transformation platform for late-stage diversification. Meanwhile, arylamines with electron-deficient groups disfavored these reactions and provided the products in low yields (4h–4j). Amines tethering on pyridine and quinoline also performed smoothly (4j–4k). However, additional CuI (0.1 equiv.) was required while alkyl amines were used. Under the modified condition, the desirable products were successfully obtained (4l–4o). Again, pyridine motif in the alkylamine did not disrupt the reaction (4l).

**Scheme 4:**
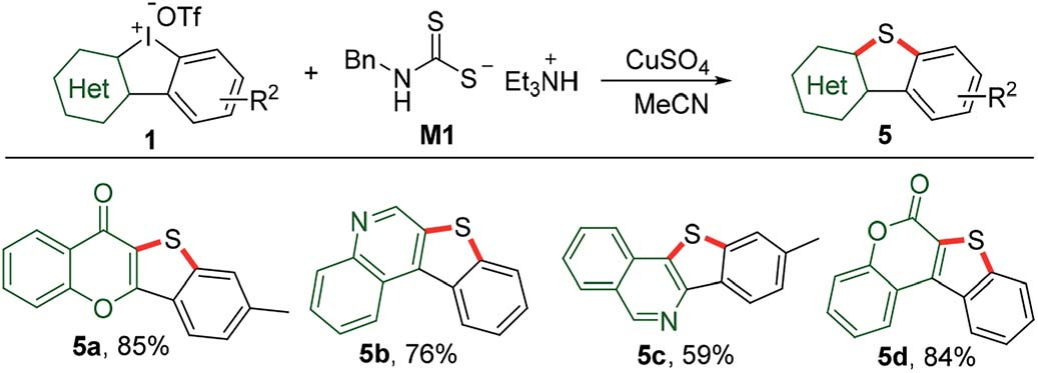
Sulfur insertion of HCIs with triethylammonium *N*-benzyldithiocarbamate M1. Reaction conditions: 1 (1 equiv.), M1 (2 equiv.), CuSO_4_ (0.1 equiv.), MeCN, 60 °C, Ar, 6 h.

Sulfur containing heterocycles have found considerable utility particularly in material science because of high resonance energy of sulfur atom.^13^ Traditional methods for the introduction of sulfur suffer from several disadvantages such as catalyst poisoning, over-oxidization, and stinky smell. Using our recently discovered odor-free triethylammonium *N*-benzyldithiocarbamate (M1) as the sulfur source donor,^8*a*^ reactions of HCIs and M1 under mediation of copper sulfate could smoothly furnish benzothiophene-fused heterocyclic frameworks, including chromone (5a), quinoline (5b), isoquinoline (5c) and coumarin (5d), as shown in Scheme 4.

**Scheme 5:**
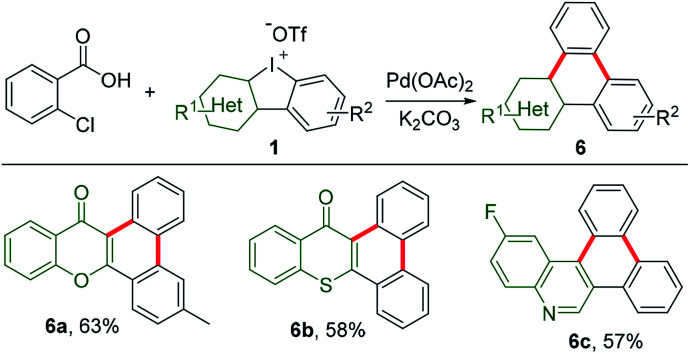
Annulation of 2-chlorobenzoic acid with HCIs. Reaction conditions: 2-chorobenzoic acid (1 equiv.), 1 (1.2 equiv.), K_2_CO_3_ (2.2 equiv.), Pd(OAc)_2_ (0.1 equiv.), 1-methyl-2-pyrrolidinone, 140 °C, 16 h.

Decarboxylation of commercially available carboxylic acids is emerging as a novel strategy for aromatic functionalization.^14^ A pioneering work has recently been extended to decarboxylation of 2-chlorobenzoic acid for *in situ* generation of benzyne to construct triphenylenes.^15^ As counterparts of these hydrocarbons, the heteroatom-containing triphenylene analogues exhibit distinct chemical and physical properties.^16^ However, they have been so far less touched due to limited synthetic protocols. Thus, HCIs could be very promising synthons to construct such unique triphenylene. In our study, HCIs reacted with 2-chlorobenzoic acid to effectively afford the desirable annulated heterocycles containing chromone (6a), thiochromone (6b), or quinoline (6c), as shown in Scheme 5.

**Scheme 6:**
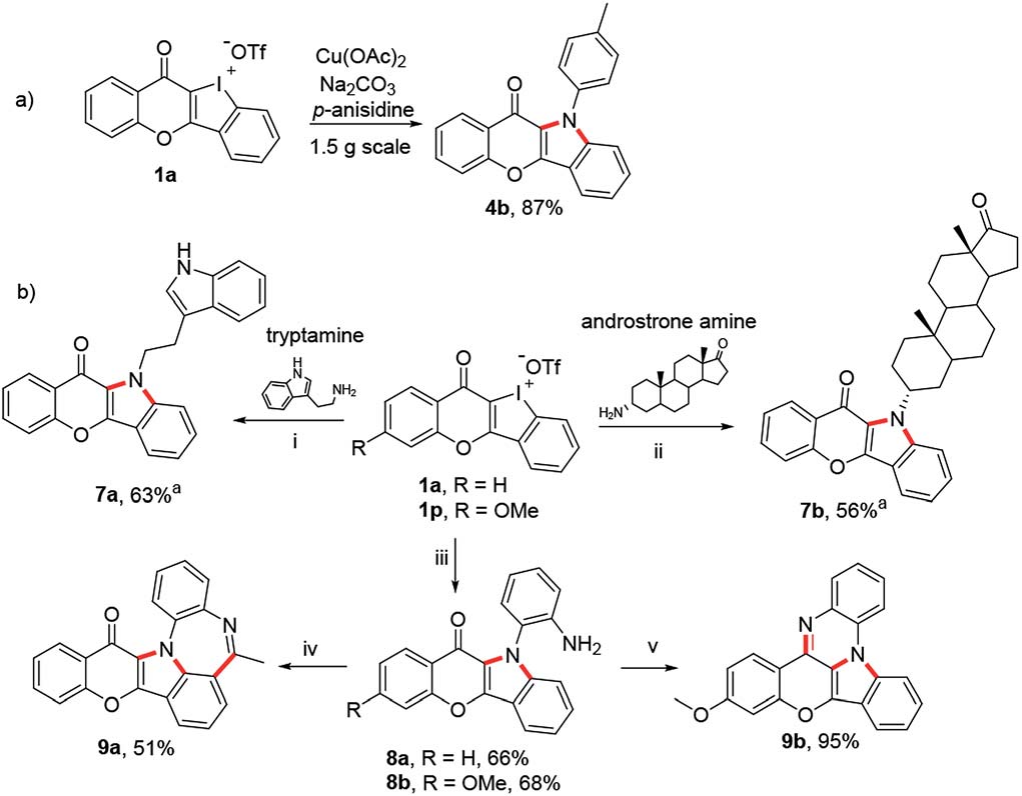
(a) Scale-up synthesis of 4b under the standard condition. (b) Synthesis of drug-like hybrids and late-stage diversification with 1a and 1p. Reaction conditions: (i)–(iii) 1a or 1p (0.2 mmol), amine (2.5 equiv.), Na_2_CO_3_ (3 equiv.), Cu(OAc)_2_ (0.1 equiv.), *i*-PrOH/(CH_2_OH)_2_ (1.8/0.2 mL), Ar, refluxing, 16 h. (iv) AcCl (1.2 equiv.), Et_3_N (2.0 equiv.), CH_2_Cl_2_, rt; PPA (0.2 mL), POCl_3_ (10 equiv.), 120 °C. (v) TsOH-H_2_O (0.1 equiv.), EtOH, reflux. ^*a*^Additional CuI (0.1 equiv.) added.

Finally, we have taken several applications to further demonstrate the robustness of these unique heterocyclic iodoniums as synthons (Scheme 6). Firstly, the copper-catalyzed dual aminations of HCIs performed well in a gram-scale reaction without a compromised yield (Scheme 6a). The increasing emergence of drug resistance in treating diseases demands an urgent need to develop new drugs. One effective strategy has been pursued combining two different drugs to form a new hybrid molecule.^17^ Herein, tryptamine, a serotonin receptor agonist, and an amine derived from androstrone (an endogenous steroid hormone) were employed to react with chromone-fused HCI 1a under the standard conditions. Both transformations successfully provided the expected fused hybrids 7a and 7b. In a final venture to establish the generality of this strategy, benzene-1,2-diamine was also used to prepare 8a and 8b which were readily for further transformation to obtain more complex heteropolycycles 9a and 9b with potential drug-likeness (Scheme 6b).

## Conclusions

In conclusion, we have fully explored the synthetic application of our recently reported heterocyclic iodoniums (HCIs). These unique iodoniums may gain more attention to build complex polycyclic heteroarenes which are widely present in naturally occurring products and pharmaceuticals. All the transformations with HCIs underwent a cyclization to build structurally diverse indole-fused, thiophene-fused, and triphenylene-fused heterocycles. Our current thorough investigation of HCIs highlights their value as versatile building blocks in synthetic chemistry, which may provide novel structures for drug development. These particular heterocycles are currently under our anticancer drug screening.

## Experimental

The ^1^H and ^13^C nuclear magnetic resonance (NMR) spectra were recorded on a Bruker Avance spectrometer 400 at 400 MHz and 100 MHz, respectively. Chemical shifts are given in ppm (*δ*) referenced to CDCl_3_ with 7.26 for ^1^H and 77.10 for ^13^C, and to *d*_6_-DMSO with 2.50 for ^1^H and 39.5 for ^13^C. In the case of multiplet, signals are reported as intervals and abbreviated as follows: s, singlet; d, doublet; t, triplet; q, quartet; m, multiplet. Coupling constants are expressed in hertz. High-resolution mass spectra (HRMS) were recorded on a BRUKER VPEXII spectrometer with ESI mode unless otherwise stated. Melting point was measured by BUCHI Melting Point B-540. The progress of the reactions was monitored by thin-layer chromatography on a glass plate coated with silica gel with fluorescent indicator (GF254). Column chromatography was performed on silica gel (200–300 mesh).

### General procedure A for the synthesis of 3a–4k

The synthesis of 10-(4-methoxyphenyl)chromeno[3,2-*b*]indol-11(10*H*)-one (3a) is exemplified. To a stirred solution of iodonium 1a (0.1 mmol) in *i*-PrOH (0.9 mL), was added ethylene glycol (0.1 mL), *p*-anisidine (2.5 equiv.), Na_2_CO_3_ (3 equiv.), and Cu(OAc)_2_ (0.1 equiv.). The reaction proceeded at reflux for 16 h under argon atmosphere before *i*-PrOH was removed by a rotary evaporation. The remained mixture was extracted with EtOAc. The combined organic layers were washed with H_2_O and brine, dried over anhydrous Na_2_SO_4_, and evaporated in a *vacuo*. The residue was purified by column chromatography (PE/EtOAc = 15/1–5/1) to provide 3a as a white solid (29 mg, 84% yield), mp 199.1–200.6 °C. ^1^H NMR (400 MHz, CDCl_3_) *δ* 8.37 (d, *J* = 8.0 Hz, 1H), 8.09 (d, *J* = 8.0 Hz, 1H), 7.74–7.68 (m, 2H), 7.51–7.45 (m, 1H), 7.44–7.37 (m, 3H), 7.32 (dd, *J* = 13.2, 7.9 Hz, 2H), 7.11–7.04 (m, 2H), 3.91 (s, 3H) ppm. ^13^C NMR (100 MHz, CDCl_3_) *δ* 169.2, 159.3, 155.5, 146.0, 139.4, 132.8, 130.0, 129.1, 128.6, 126.4, 124.6, 124.0, 121.1, 120.9, 120.0, 118.1, 115.6, 114.3, 111.6, 55.6 ppm. HRMS (ESI) *m*/*z* calcd for C_22_H_16_NO_3_ [M + H]^+^: 342.1125, found: 342.1112.

#### 10-(4-Methoxyphenyl)-8-methylchromeno[3,2-*b*]indol-11(10*H*)-one (3b)

3b (27 mg, 77% yield) was generated following a procedure for the synthesis of 3a as a white solid, mp 195.6–196.3 °C. ^1^H NMR (400 MHz, CDCl_3_) *δ* 8.36 (d, *J* = 8.2 Hz, 1H), 7.95 (d, *J* = 8.2 Hz, 1H), 7.69 (d, *J* = 2.2 Hz, 2H), 7.40 (dd, *J* = 10.9, 5.9 Hz, 3H), 7.14 (d, *J* = 8.3 Hz, 1H), 7.11–7.04 (m, 3H), 3.91 (s, 3H), 2.48 (s, 3H) ppm. ^13^C NMR (100 MHz, CDCl_3_) *δ* 168.9, 159.3, 155.5, 146.3, 140.0, 139.5, 132.6, 130.2, 129.2, 126.4, 124.7, 123.9, 123.2, 120.7, 119.7, 118.0, 114.3, 113.5, 111.1, 55.7, 22.5 ppm. HRMS (ESI) *m*/*z* calcd for C_23_H_18_NO_3_ [M + H]^+^: 356.1281, found: 356.1284.

#### 8-Chloro-10-(4-methoxyphenyl)chromeno[3,2-*b*]indol-11(10*H*)-one (3c)

3c (24 mg, 74% yield) was generated following a procedure for the synthesis of 3a as a white solid, mp 215.1–216.4 °C. ^1^H NMR (400 MHz, CDCl_3_) *δ* 8.34 (d, *J* = 7.8 Hz, 1H), 7.99 (d, *J* = 8.5 Hz, 1H), 7.75–7.64 (m, 2H), 7.41 (dd, *J* = 11.2, 8.1 Hz, 3H), 7.30 (s, 1H), 7.27 (d, *J* = 7.0 Hz, 1H), 7.08 (d, *J* = 8.7 Hz, 2H), 3.91 (s, 3H) ppm. ^13^C NMR (100 MHz, CDCl_3_) *δ* 169.1, 159.6, 155.6, 145.6, 139.6, 134.8, 133.1, 129.5, 129.0, 126.4, 124.5, 124.3, 122.2, 121.4, 121.1, 118.1, 114.5, 114.2, 111.6, 55.7 ppm. HRMS (ESI) *m*/*z* calcd for C_22_H_15_ClNO_3_ [M + H]^+^: 376.0735, found: 376.0742.

#### 8-Fluoro-10-(4-methoxyphenyl)chromeno[3,2-*b*]indol-11(10*H*)-one (3d)

3d (26 mg, 73% yield) was generated following a procedure for the synthesis of 3a as a white solid, mp 169.3–170.1 °C. ^1^H NMR (400 MHz, CDCl_3_) *δ* 8.35 (d, *J* = 7.5 Hz, 1H), 8.02 (dd, *J* = 8.8, 5.3 Hz, 1H), 7.69 (dd, *J* = 12.3, 4.9 Hz, 2H), 7.41 (t, *J* = 7.8 Hz, 3H), 7.05 (dd, *J* = 14.9, 5.4 Hz, 3H), 6.97 (dd, *J* = 9.7, 1.8 Hz, 1H), 3.91 (s, 3H) ppm. ^13^C NMR (100 MHz, CDCl_3_) *δ* 168.7, 165.0, 162.6, 159.6, 155.5, 145.9, 140.1, 140.0, 132.9, 129.7, 128.9, 126.5, 124.5, 124.2, 121.7, 121.6, 121.5, 118.1, 114.5, 112.4, 111.0, 110.8, 98.0, 97.7, 55.7 ppm. ^19^F NMR (376 MHz, CDCl_3_) *δ* −109.8, −109.8, −109.8, −109.8, −109.8, −109.9 ppm. HRMS (ESI) *m*/*z* calcd for C_22_H_15_FNO_3_ [M + H]^+^: 360.1030, found: 360.1014.

#### 7-Fluoro-10-(4-methoxyphenyl)chromeno[3,2-*b*]indol-11(10*H*)-one (3e)

3e (20 mg, 56% yield) was generated following a procedure for the synthesis of 3a as a white solid, mp 208.9–209.6 °C. ^1^H NMR (400 MHz, CDCl_3_) *δ* 8.40–8.30 (m, 2H), 7.53–7.47 (m, 2H), 7.46–7.41 (m, 2H), 7.40–7.35 (m, 2H), 7.32 (d, *J* = 8.4 Hz, 1H), 7.08 (d, *J* = 8.9 Hz, 2H), 3.92 (s, 3H) ppm. ^13^C NMR (100 MHz, CDCl_3_) *δ* 159.7, 155.0, 151.2, 142.5, 129.5, 129.2, 127.7, 124.6, 123.6, 122.7, 122.4, 122.3, 121.8, 121.6, 118.8, 117.3, 114.5, 112.5, 55.6 ppm. HRMS (ESI) *m*/*z* calcd for C_22_H_15_FNO_3_ [M + H]^+^: 360.1030, found: 360.1036.

#### 10-(4-Methoxyphenyl)thiochromeno[3,2-*b*]indol-11(10*H*)-one (3f)

3f (19 mg, 53% yield) was generated following a procedure for the synthesis of 3a as a white solid, mp 224.9–225.5 °C. ^1^H NMR (400 MHz, CDCl_3_) *δ* 8.64 (d, *J* = 8.1 Hz, 1H), 7.91 (d, *J* = 8.0 Hz, 1H), 7.77 (d, *J* = 8.0 Hz, 1H), 7.65–7.56 (m, 1H), 7.48 (dd, *J* = 18.4, 7.5 Hz, 2H), 7.35 (d, *J* = 8.8 Hz, 2H), 7.33–7.29 (m, 1H), 7.25 (d, *J* = 9.7 Hz, 1H), 7.08 (d, *J* = 8.8 Hz, 2H), 3.92 (s, 3H) ppm. ^13^C NMR (100 MHz, CDCl_3_) *δ* 172.3, 159.4, 141.2, 135.9, 132.8, 131.4, 130.8, 129.2, 129.2, 128.6, 128.2, 126.9, 126.2, 123.2, 121.2, 120.6, 120.3, 114.3, 112.2, 55.6 ppm. HRMS (ESI) *m*/*z* calcd for C_22_H_16_NO_2_S [M + H]^+^: 358.0896, found: 358.0889.

#### 7-(4-Methoxyphenyl)-7*H*-indolo[2,3-*c*]quinolone (3g)

3g (25 mg, 77% yield) was generated following a procedure for the synthesis of 3a as a white solid, mp 130.4–131.2 °C. ^1^H NMR (400 MHz, CDCl_3_) *δ* 9.08 (s, 1H), 8.77 (d, *J* = 8.2 Hz, 1H), 8.64 (d, *J* = 8.0 Hz, 1H), 8.31 (d, *J* = 8.3 Hz, 1H), 7.76 (t, *J* = 7.5 Hz, 1H), 7.69 (t, *J* = 7.6 Hz, 1H), 7.59–7.54 (m, 1H), 7.48 (dd, *J* = 18.5, 7.9 Hz, 4H), 7.17 (d, *J* = 8.7 Hz, 2H), 3.95 (s, 3H) ppm. ^13^C NMR (100 MHz, CDCl_3_) *δ* 159.7, 143.5, 141.4, 137.4, 134.1, 130.5, 129.1, 128.9, 127.4, 127.2, 125.9, 124.7, 123.5, 123.4, 122.2, 121.4, 121.3, 115.5, 111.3, 55.8 ppm. HRMS (ESI) *m*/*z* calcd for C_22_H_17_N_2_O [M + H]^+^: 325.1335, found: 325.1330.

#### 7-(4-Methoxyphenyl)-9-methyl-7*H*-indolo[2,3-*c*]quinolone (3h)

3h (25 mg, 74% yield) was generated following a procedure for the synthesis of 3a as a white solid, mp 177.9–179.1 °C. ^1^H NMR (400 MHz, CDCl_3_) *δ* 9.02 (s, 1H), 8.71 (d, *J* = 8.1 Hz, 1H), 8.47 (d, *J* = 8.7 Hz, 1H), 8.28 (d, *J* = 8.2 Hz, 1H), 7.72 (t, *J* = 7.4 Hz, 1H), 7.65 (t, *J* = 7.5 Hz, 1H), 7.47 (d, *J* = 8.8 Hz, 2H), 7.26 (t, *J* = 7.6 Hz, 2H), 7.15 (d, *J* = 8.8 Hz, 2H), 3.93 (s, 3H), 2.53 (s, 3H) ppm. ^13^C NMR (100 MHz, CDCl_3_) *δ* 159.6, 143.5, 141.9, 137.8, 137.3, 134.1, 130.5, 129.2, 128.9, 127.1, 125.8, 124.6, 123.5, 123.1, 123.1, 121.5, 120.0, 115.4, 111.1, 55.8, 22.3 ppm. HRMS (ESI) *m*/*z* calcd for C_23_H_19_N_2_O [M + H]^+^: 339.1492, found: 339.1487.

#### 9-Chloro-7-(4-methoxyphenyl)-7*H*-indolo[2,3-*c*]quinolone (3i)

3i (18 mg, 51% yield) was generated following a procedure for the synthesis of 3a as a white solid, mp 204.7–205.6 °C. ^1^H NMR (400 MHz, CDCl_3_) *δ* 9.02 (s, 1H), 8.65 (d, *J* = 8.0 Hz, 1H), 8.48 (d, *J* = 8.5 Hz, 1H), 8.30 (d, *J* = 8.1 Hz, 1H), 7.74 (t, *J* = 7.4 Hz, 1H), 7.68 (t, *J* = 7.4 Hz, 1H), 7.46 (d, *J* = 8.5 Hz, 3H), 7.40 (d, *J* = 8.6 Hz, 1H), 7.17 (d, *J* = 8.7 Hz, 2H), 3.95 (s, 3H) ppm. ^13^C NMR (100 MHz, CDCl_3_) *δ* 160.0, 143.3, 141.9, 137.1, 134.4, 133.4, 130.4, 128.8, 128.4, 127.6, 126.4, 124.3, 123.3, 122.1, 121.2, 120.7, 115.6, 111.3, 55.8 ppm. HRMS (ESI) *m*/*z* calcd for C_22_H_16_ClN_2_O [M + H]^+^: 359.0946, found: 359.0940.

#### 2-Fluoro-7-(4-methoxyphenyl)-7*H*-indolo[2,3-*c*]quinolone (3j)

3j (21 mg, 62% yield) was generated following a procedure for the synthesis of 3a as a white solid, mp 161.1–161.8 °C. ^1^H NMR (400 MHz, CDCl_3_) *δ* 9.01 (s, 1H), 8.52 (d, *J* = 7.9 Hz, 1H), 8.28 (ddd, *J* = 15.1, 9.5, 4.2 Hz, 2H), 7.60–7.53 (m, 1H), 7.52–7.45 (m, 4H), 7.41 (td, *J* = 8.8, 2.7 Hz, 1H), 7.17 (d, *J* = 8.8 Hz, 2H), 3.95 (s, 3H) ppm. ^13^C NMR (100 MHz, CDCl_3_) *δ* 162.7, 160.3, 159.8, 141.3, 140.4, 136.7, 134.2, 132.8, 132.7, 128.9, 128.8, 127.3, 125.4, 125.3, 122.9, 122.0, 121.6, 121.0, 115.6, 115.5, 115.3, 111.4, 107.6, 107.4, 55.8 ppm. ^19^F NMR (376 MHz, CDCl_3_) *δ* −112.2, −112.2, −112.2, −112.3 ppm. HRMS (ESI) *m*/*z* calcd for C_22_H_16_FN_2_O [M + H]^+^: 343.1241, found: 343.1229.

#### 11-(4-Methoxyphenyl)-11*H*-indolo[3,2-*c*]isoquinoline (3k)

3k (25 mg, 78% yield) was generated following a procedure for the synthesis of 3a as a white solid, mp 185.8–186.9 °C. ^1^H NMR (400 MHz, CDCl_3_) *δ* 9.17 (s, 1H), 8.49 (d, *J* = 7.4 Hz, 1H), 8.12 (d, *J* = 8.0 Hz, 1H), 7.53 (t, *J* = 7.3 Hz, 1H), 7.50–7.39 (m, 6H), 7.21 (d, *J* = 8.0 Hz, 1H), 7.16 (d, *J* = 8.7 Hz, 2H), 3.97 (s, 3H) ppm. ^13^C NMR (100 MHz, CDCl_3_) *δ* 160.2, 146.2, 142.0, 135.1, 131.6, 130.2, 129.4, 129.1, 128.4, 127.7, 126.1, 125.7, 124.5, 122.9, 121.2, 121.0, 119.9, 115.4, 110.4, 55.8 ppm. HRMS (ESI) *m*/*z* calcd for C_22_H_17_N_2_O [M + H]^+^: 325.1335, found: 325.1327.

#### 11-(4-Methoxyphenyl)-9-methyl-11*H*-indolo[3,2-*c*]isoquinoline (3l)

3l (25 mg, 74% yield) was generated following a procedure for the synthesis of 3a as a white solid, mp 173.8–175.2 °C. ^1^H NMR (400 MHz, CDCl_3_) *δ* 9.15 (s, 1H), 8.37 (d, *J* = 8.0 Hz, 1H), 8.11 (d, *J* = 8.1 Hz, 1H), 7.54–7.49 (m, 1H), 7.48–7.42 (m, 3H), 7.39 (d, *J* = 8.4 Hz, 1H), 7.26–7.22 (m, 1H), 7.17 (d, *J* = 8.8 Hz, 2H), 6.99 (s, 1H), 3.98 (s, 3H), 2.50 (s, 3H) ppm. ^13^C NMR (100 MHz, CDCl_3_) *δ* 160.1, 145.8, 142.5, 136.6, 135.1, 131.7, 130.3, 129.4, 129.2, 128.2, 127.5, 125.4, 124.6, 122.8, 121.1, 120.6, 119.7, 115.4, 110.4, 55.8, 22.3 ppm. HRMS (ESI) *m*/*z* calcd for C_23_H_19_N_2_O [M + H]^+^: 339.1492, found: 339.1489.

#### 7-(4-Methoxyphenyl)chromeno[3,4-*b*]indol-6(7*H*)-one (3m)

3m (26 mg, 75% yield) was generated following a procedure for the synthesis of 3a as a white solid, mp 231.1–232.5 °C. ^1^H NMR (400 MHz, CDCl_3_) *δ* 8.35 (dd, *J* = 8.0, 1.3 Hz, 1H), 7.76–7.64 (m, 3H), 7.44–7.35 (m, 3H), 7.29–7.18 (m, 3H), 7.07 (d, *J* = 8.9 Hz, 2H), 3.91 (s, 3H) ppm. ^13^C NMR (100 MHz, CDCl_3_) *δ* 169.4, 159.5, 155.6, 145.5, 136.0, 133.1, 129.8, 129.1, 126.4, 124.5, 124.1, 122.1, 118.1, 117.8, 117.5, 115.5, 115.4, 114.4, 113.1, 113.0, 104.7, 104.4, 55.7 ppm. HRMS (ESI) *m*/*z* calcd for C_22_H_16_NO_3_[M + H]^+^: 342.1125, found: 342.1124.

#### 4-(4-Methoxyphenyl)-4*H*-thieno[3,2-*b*]indole (3n)

3n (16 mg, 57% yield) was generated following a procedure for the synthesis of 3a as a white solid, mp 125.1–126.9 °C. ^1^H NMR (400 MHz, CDCl_3_) *δ* 7.80 (d, *J* = 7.4 Hz, 1H), 7.48 (t, *J* = 8.6 Hz, 3H), 7.36 (d, *J* = 5.2 Hz, 1H), 7.29–7.19 (m, 2H), 7.08 (d, *J* = 8.8 Hz, 2H), 7.04 (d, *J* = 5.2 Hz, 1H), 3.91 (s, 3H) ppm. ^13^C NMR (100 MHz, CDCl_3_) *δ* 158.5, 145.6, 141.8, 131.8, 126.9, 126.8, 124.4, 123.0, 122.2, 120.1, 119.0, 117.4, 115.0, 114.7, 111.4, 111.0, 55.7 ppm. HRMS (ESI) *m*/*z* calcd for C_17_H_14_NOS [M + H]^+^: 280.0791, found: 280.0787.

#### 10-Phenylchromeno[3,2-*b*]indol-11(10*H*)-one (4a)

4a (24 mg, 79% yield) was generated following a procedure for the synthesis of 3a as a white solid, mp 190.7–191.6 °C. ^1^H NMR (400 MHz, CDCl_3_) *δ* 8.37 (d, *J* = 7.8 Hz, 1H), 8.10 (d, *J* = 8.1 Hz, 1H), 7.72 (d, *J* = 3.0 Hz, 2H), 7.60–7.54 (m, 2H), 7.53–7.46 (m, 4H), 7.45–7.40 (m, 1H), 7.38 (d, *J* = 8.5 Hz, 1H), 7.33 (t, *J* = 7.5 Hz, 1H) ppm. ^13^C NMR (100 MHz, CDCl_3_) *δ* 169.1, 155.5, 146.3, 139.0, 137.1, 132.8, 129.0, 128.7, 128.1, 128.0, 126.4, 124.5, 124.0, 121.2, 120.6, 120.0, 118.1, 115.7, 111.5 ppm. HRMS (ESI) *m*/*z* calcd for C_21_H_14_NO_2_ [M + H]^+^: 312.1019, found: 312.1017.

#### 10-(*p*-Tolyl)chromeno[3,2-*b*]indol-11(10*H*)-one (4b)

4b (30 mg, 92% yield) was generated following a procedure for the synthesis of 3a as a white solid, mp 205.2–206.6 °C. ^1^H NMR (400 MHz, CDCl_3_) *δ* 8.37 (d, *J* = 7.9 Hz, 1H), 8.09 (d, *J* = 8.1 Hz, 1H), 7.71 (d, *J* = 3.0 Hz, 2H), 7.51–7.45 (m, 1H), 7.44–7.40 (m, 1H), 7.41–7.34 (m, 5H), 7.31 (t, *J* = 7.5 Hz, 1H), 2.48 (s, 3H) ppm. ^13^C NMR (100 MHz, CDCl_3_) *δ* 169.1, 155.5, 146.1, 139.1, 138.0, 134.5, 132.8, 129.7, 128.6, 127.7, 126.4, 124.5, 124.0, 121.1, 120.7, 120.0, 118.0, 115.6, 111.6, 21.4 ppm. HRMS (ESI) *m*/*z* calcd for C_22_H_16_NO_2_ [M + H]^+^: 326.1176, found: 326.1171.

#### 10-(4-Fluorophenyl)chromeno[3,2-*b*]indol-11(10*H*)-one (4c)

4c (24 mg, 74% yield) was generated following a procedure for the synthesis of 3a as a white solid, mp 227.5–228.6 °C. ^1^H NMR (400 MHz, CDCl_3_) *δ* 8.37 (d, *J* = 7.9 Hz, 1H), 8.09 (d, *J* = 8.1 Hz, 1H), 7.72 (t, *J* = 5.8 Hz, 2H), 7.57–7.40 (m, 4H), 7.38–7.30 (m, 2H), 7.26 (dd, *J* = 11.0, 6.0 Hz, 2H) ppm. ^13^C NMR (100 MHz, CDCl_3_) *δ* 169.2, 163.4, 161.0, 155.6, 146.3, 139.2, 133.2, 133.0, 129.8, 129.7, 128.9, 126.3, 124.5, 124.2, 121.4, 120.7, 120.2, 118.2, 116.1, 115.9, 115.8, 111.4 ppm. ^19^F NMR (376 MHz, CDCl_3_) *δ* −113.4, −113.5, −113.5, −113.5, −113.5, −113.5, −113.5 ppm. HRMS (ESI) *m*/*z* calcd for C_21_H_13_FNO_2_ [M + H]^+^: 330.0925, found: 330.0934.

#### 10-(4-Chlorophenyl)chromeno[3,2-*b*]indol-11(10*H*)-one (4d)

4d (23 mg, 65% yield) was generated following a procedure for the synthesis of 3a as a white solid, mp 265.6–266.2 °C. ^1^H NMR (400 MHz, CDCl_3_) *δ* 8.40–8.30 (m, 1H), 8.09 (d, *J* = 8.0 Hz, 1H), 7.72 (dd, *J* = 5.7, 1.4 Hz, 2H), 7.53 (d, *J* = 8.6 Hz, 2H), 7.51–7.48 (m, 1H), 7.47–7.40 (m, 3H), 7.39–7.31 (m, 2H) ppm. ^13^C NMR (100 MHz, CDCl_3_) *δ* 169.2, 155.6, 146.5, 139.0, 135.7, 133.9, 133.1, 129.3, 129.0, 126.4, 124.5, 124.3, 121.6, 120.6, 120.2, 118.2, 116.0, 111.4 ppm. HRMS (ESI) *m*/*z* calcd for C_21_H_13_ClNO_2_ [M + H]^+^: 346.0629, found: 346.0621.

#### 10-(4-Bromophenyl)chromeno[3,2-*b*]indol-11(10*H*)-one (4e)

4e (22 mg, 65% yield) was generated following a procedure for the synthesis of 3a as a white solid, mp 283.5–284.7 °C. ^1^H NMR (400 MHz, CDCl_3_) *δ* 8.36 (d, *J* = 8.0 Hz, 1H), 8.09 (d, *J* = 8.0 Hz, 1H), 7.78–7.64 (m, 4H), 7.50 (t, *J* = 7.7 Hz, 1H), 7.46–7.29 (m, 5H) ppm. ^13^C NMR (100 MHz, CDCl_3_) *δ* 169.2, 155.6, 146.5, 138.9, 136.2, 133.1, 132.3, 129.6, 129.1, 126.4, 124.5, 124.3, 121.9, 121.6, 120.5, 120.3, 118.2, 116.1, 111.4 ppm. HRMS (ESI) *m*/*z* calcd for C_21_H_13_BrNO_2_ [M + H]^+^: 390.0124, found: 390.0127.

#### 10-(4-Iodophenyl)chromeno[3,2-*b*]indol-11(10*H*)-one (4f)

4f (24 mg, 54% yield) was generated following a procedure for the synthesis of 3a as a white solid, mp 241.1–242.4 °C. ^1^H NMR (400 MHz, CDCl_3_) *δ* 8.36 (d, *J* = 7.7 Hz, 1H), 8.10 (d, *J* = 8.0 Hz, 1H), 7.88 (d, *J* = 8.4 Hz, 2H), 7.72 (t, *J* = 6.1 Hz, 2H), 7.54–7.48 (m, 1H), 7.46–7.41 (m, 1H), 7.35 (dd, *J* = 17.3, 8.1 Hz, 2H), 7.27 (d, *J* = 6.7 Hz, 2H) ppm. ^13^C NMR (100 MHz, CDCl_3_) *δ* 169.1, 155.5, 146.6, 138.8, 138.2, 136.9, 133.0, 129.8, 129.0, 126.4, 124.5, 124.3, 121.6, 120.5, 120.3, 118.2, 116.1, 111.4, 93.3 ppm. HRMS (ESI) *m*/*z* calcd for C_21_H_13_INO_2_ [M + H]^+^: 437.9986, found: 437.9986.

#### 10-(4-(Trifluoromethyl)phenyl)chromeno[3,2-*b*]indol-11(10*H*)-one (4g)

4g (20 mg, 52% yield) was generated following a procedure for the synthesis of 3a as a white solid, mp 247.8–248.5 °C. ^1^H NMR (400 MHz, CDCl_3_) *δ* 8.40–8.33 (m, 1H), 8.12 (d, *J* = 8.1 Hz, 1H), 7.83 (d, *J* = 8.4 Hz, 2H), 7.74 (dt, *J* = 8.0, 4.0 Hz, 2H), 7.65 (d, *J* = 8.3 Hz, 2H), 7.52 (dd, *J* = 11.4, 4.1 Hz, 1H), 7.47–7.43 (m, 1H), 7.43–7.34 (m, 2H) ppm. ^13^C NMR (100 MHz, CDCl_3_) *δ* 169.2, 155.5, 147.0, 140.2, 138.8, 133.2, 129.3, 128.3, 126.4, 126.2, 124.4, 121.9, 120.4, 118.2, 116.3, 111.3 ppm. ^19^F NMR (376 MHz, CDCl_3_) *δ* −62.4 ppm. HRMS (ESI) *m*/*z* calcd for C_22_H_13_F_3_NO_2_ [M + H]^+^: 380.0893, found: 380.0908.

#### Methyl 4-(11-oxochromeno[3,2-*b*]indol-10(11*H*)-yl)benzoate (4h)

4h (15 mg, 41% yield) was generated following a procedure for the synthesis of 3a as a white solid, mp 232.4–233.1 °C. ^1^H NMR (400 MHz, CDCl_3_) *δ* 8.37 (d, *J* = 7.5 Hz, 1H), 8.24 (d, *J* = 8.5 Hz, 2H), 8.11 (d, *J* = 8.0 Hz, 1H), 7.78–7.70 (m, 2H), 7.60 (d, *J* = 8.5 Hz, 2H), 7.55–7.49 (m, 1H), 7.48–7.40 (m, 2H), 7.36 (t, *J* = 7.5 Hz, 1H), 3.98 (s, 3H) ppm. ^13^C NMR (100 MHz, CDCl_3_) *δ* 169.1, 166.6, 155.6, 147.0, 141.2, 138.8, 133.1, 130.5, 129.5, 129.2, 127.8, 126.5, 124.5, 124.3, 121.8, 120.5, 120.3, 118.2, 116.3, 111.5, 52.4 ppm. HRMS (ESI) *m*/*z* calcd for C_23_H_16_NO_4_ [M + H]^+^: 370.1074, found: 370.1074.

#### Methyl 2-(11-oxochromeno[3,2-*b*]indol-10(11*H*)-yl)benzoate (4i)

4i (12 mg, 32% yield) was generated following a procedure for the synthesis of 3a as a white solid, mp 219.9–221.1 °C. ^1^H NMR (400 MHz, CDCl_3_) *δ* 8.33 (d, *J* = 7.9 Hz, 1H), 8.20 (dd, *J* = 7.8, 1.4 Hz, 1H), 8.10 (d, *J* = 8.0 Hz, 1H), 7.78–7.66 (m, 3H), 7.61 (td, *J* = 7.7, 1.1 Hz, 1H), 7.57 (d, *J* = 7.8 Hz, 1H), 7.50–7.43 (m, 1H), 7.43–7.37 (m, 1H), 7.31 (t, *J* = 7.5 Hz, 1H), 7.15 (d, *J* = 8.5 Hz, 1H), 3.46 (s, 3H) ppm. ^13^C NMR (100 MHz, CDCl_3_) *δ* 169.3, 165.6, 155.7, 146.0, 139.2, 137.3, 132.9, 132.8, 131.6, 130.5, 129.5, 128.8, 128.7, 126.3, 124.4, 124.0, 121.5, 121.2, 120.1, 118.2, 115.8, 111.1, 52.2 ppm. HRMS (ESI) *m*/*z* calcd for C_23_H_16_NO_4_ [M + H]^+^: 370.1074, found: 370.1078.

#### 10-(Pyridin-2-yl)chromeno[3,2-*b*]indol-11(10*H*)-one (4j)

4j (11 mg, 34% yield) was generated following a procedure for the synthesis of 3a as a white solid, mp 191.3–192.5 °C. ^1^H NMR (400 MHz, CDCl_3_) *δ* 8.67 (s, 1H), 8.39 (d, *J* = 7.9 Hz, 1H), 8.09 (d, *J* = 8.0 Hz, 1H), 7.93 (t, *J* = 7.2 Hz, 1H), 7.84 (d, *J* = 8.4 Hz, 1H), 7.72 (s, 2H), 7.54 (t, *J* = 7.6 Hz, 2H), 7.45 (dd, *J* = 14.0, 6.4 Hz, 1H), 7.42–7.33 (m, 2H) ppm. ^13^C NMR (100 MHz, CDCl_3_) *δ* 169.0, 155.5, 150.6, 148.4, 147.7, 138.8, 137.9, 133.0, 129.5, 126.5, 124.6, 124.3, 122.6, 122.5, 122.1, 120.1, 120.0, 118.2, 116.7, 113.0 ppm. HRMS (ESI) *m*/*z* calcd for C_20_H_13_N_2_O_2_ [M + H]^+^: 313.0972, found: 313.0963.

#### 10-(Quinolin-8-yl)chromeno[3,2-*b*]indol-11(10*H*)-one (4k)

4k (24 mg, 67% yield) was generated following a procedure for the synthesis of 3a as a white solid, mp 213.5–214.5 °C. ^1^H NMR (400 MHz, CDCl_3_) *δ* 8.76 (d, *J* = 3.0 Hz, 1H), 8.28 (dd, *J* = 18.7, 7.9 Hz, 2H), 8.15 (d, *J* = 8.0 Hz, 1H), 8.03 (d, *J* = 8.2 Hz, 1H), 7.91 (d, *J* = 7.1 Hz, 1H), 7.73 (dd, *J* = 15.9, 8.4 Hz, 3H), 7.48–7.30 (m, 4H), 7.08 (d, *J* = 8.4 Hz, 1H) ppm. ^13^C NMR (100 MHz, CDCl_3_) *δ* 169.3, 155.8, 150.9, 146.2, 145.2, 139.9, 136.7, 135.1, 132.7, 129.8, 129.3, 129.0, 128.5, 126.3, 126.3, 124.7, 123.9, 122.6, 121.9, 121.2, 120.2, 118.2, 116.2, 111.8 ppm. HRMS (ESI) *m*/*z* calcd for C_24_H_15_N_2_O_2_ [M + H]^+^: 363.1128, found: 363.1113.

### General procedure B for the synthesis of 4l–4o

The synthesis of 10-(2-(pyridin-2-yl)ethyl)chromeno[3,2-*b*]indol-11(10*H*)-one (4l) is exemplified. To a stirred solution of iodonium 1a (0.1 mmol) in *i*-PrOH (0.9 mL), was added ethylene glycol (0.1 mL), 2-(pyridin-2-yl)ethan-1-amine (2.5 equiv.), Na_2_CO_3_ (3 equiv.), Cu(OAc)_2_ (0.1 equiv.), and CuI (0.1 equiv.). The reaction proceeded at reflux for 16 h under argon atmosphere before *i*-PrOH was removed by a rotary evaporation. The remained mixture was extracted with EtOAc. The combined organic layers were washed with H_2_O and brine, dried over anhydrous Na_2_SO_4_, and evaporated in a *vacuo*. The residue was purified by column chromatography (PE/EtOAc = 15/1–5/1) to provide 4l as a white solid (23 mg, 68% yield), mp 139.7–140.8 °C. ^1^H NMR (400 MHz, CDCl_3_) *δ* 8.55 (d, *J* = 4.3 Hz, 1H), 8.45 (d, *J* = 7.9 Hz, 1H), 7.95 (d, *J* = 8.0 Hz, 1H), 7.75–7.59 (m, 2H), 7.43 (t, *J* = 7.5 Hz, 2H), 7.34 (dd, *J* = 20.0, 7.8 Hz, 2H), 7.17 (t, *J* = 7.4 Hz, 1H), 7.06 (t, *J* = 8.4 Hz, 2H), 5.08 (t, *J* = 7.3 Hz, 2H), 3.37 (t, *J* = 7.2 Hz, 2H) ppm. ^13^C NMR (100 MHz, CDCl3) *δ* 170.1, 158.8, 155.6, 149.5, 145.2, 138.0, 136.5, 132.7, 128.1, 126.1, 124.4, 123.9, 123.9, 121.7, 120.2, 120.0, 119.9, 118.1, 114.7, 110.5, 45.0, 39.9 ppm. HRMS (ESI) *m*/*z* calcd for C_22_H_17_N_2_O_2_ [M + H]^+^: 341.1285, found: 341.1278.

#### 10-Butylchromeno[3,2-*b*]indol-11(10*H*)-one (4m)

4m (20 mg, 68% yield) was generated following a procedure for the synthesis of 4l as a white solid, mp 87.4–88.6 °C. ^1^H NMR (400 MHz, CDCl_3_) *δ* 8.45 (dd, *J* = 8.0, 1.3 Hz, 1H), 8.02 (d, *J* = 8.1 Hz, 1H), 7.74–7.61 (m, 2H), 7.57–7.46 (m, 2H), 7.45–7.40 (m, 1H), 7.25 (ddd, *J* = 7.9, 5.3, 1.1 Hz, 1H), 4.76 (t, *J* = 7.3 Hz, 2H), 1.94–1.76 (m, 2H), 1.41 (dd, *J* = 15.3, 7.5 Hz, 2H), 0.95 (t, *J* = 7.4 Hz, 3H) ppm. ^13^C NMR (100 MHz, CDCl_3_) *δ* 170.3, 155.6, 145.0, 137.9, 132.7, 128.1, 126.3, 124.5, 123.9, 120.4, 120.2, 118.2, 115.0, 110.6, 44.8, 33.1, 20.2, 14.0 ppm. HRMS (ESI) *m*/*z* calcd for C_19_H_18_NO_2_ [M + H]^+^: 292.1332, found: 292.1337.

#### 10-Cyclopropylchromeno[3,2-*b*]indol-11(10*H*)-one (4n)

4n (23 mg, 82% yield) was generated following a procedure for the synthesis of 4l as a white solid, mp 167.9–168.4 °C. ^1^H NMR (400 MHz, CDCl_3_) *δ* 8.46 (dd, *J* = 8.0, 1.4 Hz, 1H), 8.03–7.98 (m, 1H), 7.73 (d, *J* = 8.5 Hz, 1H), 7.71–7.63 (m, 2H), 7.52 (ddd, *J* = 8.4, 5.4, 1.2 Hz, 1H), 7.43 (ddd, *J* = 8.1, 6.8, 1.4 Hz, 1H), 7.30–7.23 (m, 1H), 3.63–3.54 (m, 1H), 1.40–1.33 (m, 2H), 1.17 (qd, *J* = 5.6, 4.5 Hz, 2H) ppm. ^13^C NMR (100 MHz, CDCl_3_) *δ* 169.5, 155.4, 145.5, 139.1, 132.7, 128.1, 126.4, 124.8, 124.0, 121.6, 120.6, 120.1, 118.0, 115.2, 112.3, 26.6, 9.5 ppm. HRMS (ESI) *m*/*z* calcd for C_18_H_14_NO_2_ [M + H]^+^: 276.1019, found: 276.1011.

#### 10-(4-Hydroxybutyl)chromeno[3,2-*b*]indol-11(10*H*)-one (4o)

4o (27 mg, 88% yield) was generated following a procedure for the synthesis of 4l as a white solid, mp 110.7–111.3 °C. ^1^H NMR (400 MHz, CDCl_3_) *δ* 8.44 (d, *J* = 7.8 Hz, 1H), 8.03 (d, *J* = 8.1 Hz, 1H), 7.76–7.62 (m, 2H), 7.57–7.46 (m, 2H), 7.44 (t, *J* = 7.2 Hz, 1H), 7.30–7.18 (m, 1H), 4.81–4.73 (m, 2H), 3.75 (t, *J* = 6.2 Hz, 2H), 2.01 (dt, *J* = 14.8, 7.4 Hz, 2H), 1.74–1.55 (m, 2H) ppm. ^13^C NMR (100 MHz, CDCl_3_) *δ* 170.3, 155.5, 145.2, 137.9, 132.8, 128.4, 126.2, 124.2, 123.9, 120.3, 120.2, 118.1, 114.9, 110.4, 62.1, 44.2, 29.4, 27.2 ppm. HRMS (ESI) *m*/*z* calcd for C_19_H_18_NO_3_ [M + H]^+^: 308.1281, found: 308.1278.

#### Procedure for the synthesis of triethylammonium benzylcarbamodithioate (M1)

To a solution of benzylamine (1.11 g, 10.38 mmol, 1.05 equiv.) and Et_3_N (1.0 g, 9.88 mmol, 1 equiv.) in CH_2_Cl_2_ (25 mL), CS_2_ (0.83 g, 10.87 mmol, 1.1 equiv.) was dropped slowly. The solution was stirred at room temperature for 3 h, concentrated by a rotary evaporator, and finally dried by a high vacuum to give M1 (2.75 g, 98% yield) as a white solid.

#### The reaction procedure of heterocyclic iodoniums and M1 to provide benzothiophene-fused heterocycles 5

Syntheses of 8-methyl-11*H*-benzo[4,5]thieno[3,2-*b*]chromen-11-one (5a) is exemplified. To a stirred solution of iodonium 1b (0.1 mmol) in MeCN (2.0 mL), was added M1 (2 equiv.) and CuSO_4_ (0.1 equiv.). The reaction proceeded at 70 °C for 6 h under argon atmosphere before MeCN was removed by a rotary evaporation. The remained mixture was extracted with EtOAc. The combined organic layers were washed with H_2_O and brine, dried over anhydrous Na_2_SO_4_, and evaporated in a *vacuo*. The residue was purified by column chromatography (PE/EtOAc = 20/1–5/1) to provide 5a as a white solid (22 mg, 85% yield), mp 191.2–192.1 °C. ^1^H NMR (400 MHz, CDCl_3_) *δ* 8.39 (dd, *J* = 8.0, 1.5 Hz, 1H), 8.07 (d, *J* = 8.2 Hz, 1H), 7.79–7.72 (m, 1H), 7.72–7.64 (m, 2H), 7.48 (dd, *J* = 11.0, 3.9 Hz, 1H), 7.35 (d, *J* = 8.2 Hz, 1H), 2.54 (s, 3H) ppm. ^13^C NMR (100 MHz, CDCl_3_) *δ* 173.3, 156.0, 153.9, 140.4, 140.2, 133.7, 127.0, 126.0, 124.9, 123.6, 122.7, 122.1, 119.9, 118.1, 22.1 ppm. HRMS (ESI) *m*/*z* calcd for C_16_H_11_O_2_S[M + H]^+^: 267.0474, found: 267.0481.

#### Benzo[4,5]thieno[2,3-*c*]quinolone (5b)

5b (18 mg, 76% yield) was generated following a procedure for the synthesis of 5a as a white solid, mp 129.7–131.2 °C. ^1^H NMR (400 MHz, CDCl_3_) *δ* 9.40 (s, 1H), 8.98–8.93 (m, 1H), 8.91 (dd, *J* = 5.8, 2.9 Hz, 1H), 8.49–8.38 (m, 1H), 8.10 (dd, *J* = 5.5, 3.6 Hz, 1H), 7.82 (dd, *J* = 5.7, 3.9 Hz, 2H), 7.75–7.63 (m, 2H) ppm. ^13^C NMR (100 MHz, CDCl_3_) *δ* 145.5, 145.4, 141.5, 135.6, 135.2, 133.4, 130.7, 127.9, 127.7, 126.1, 125.5, 123.9, 123.0 ppm. HRMS (ESI) *m*/*z* calcd for C_15_H_10_NS [M + H]^+^: 236.0528, found: 236.0520.

#### 9-Methylbenzo[4,5]thieno[3,2-*c*]isoquinoline (5c)

5c (15 mg, 59% yield) was generated following a procedure for the synthesis of 5a as a white solid, mp 108.8–110.3 °C. ^1^H NMR (400 MHz, CDCl_3_) *δ* 9.31 (s, 1H), 8.53 (d, *J* = 8.1 Hz, 1H), 8.16 (d, *J* = 8.1 Hz, 1H), 8.09 (d, *J* = 8.3 Hz, 1H), 7.85 (t, *J* = 7.6 Hz, 1H), 7.76 (s, 1H), 7.69 (t, *J* = 7.6 Hz, 1H), 7.42 (d, *J* = 8.2 Hz, 1H), 2.57 (s, 3H) ppm. ^13^C NMR (100 MHz, CDCl_3_) *δ* 150.2, 138.7, 137.9, 133.5, 132.0, 131.2, 129.5, 129.1, 127.9, 127.3, 127.2, 126.9, 126.8, 123.7, 123.6, 123.0, 122.8, 122.7, 122.4, 22.0 ppm. HRMS (ESI) *m*/*z* calcd for C_16_H_12_NS [M + H]^+^: 250.0685, found: 250.0677.

#### 6*H*-Benzo[4,5]thieno[2,3-*c*]chromen-6-one (5d)

5d (21 mg, 84% yield) was generated following a procedure for the synthesis of 5a as a white solid, mp 205.0–206.8 °C. ^1^H NMR (400 MHz, CDCl_3_) *δ* 8.69–8.59 (m, 1H), 8.50 (d, *J* = 8.0 Hz, 1H), 8.06–7.97 (m, 1H), 7.67–7.60 (m, 2H), 7.56 (q, *J* = 8.3 Hz, 2H), 7.46 (t, *J* = 7.2 Hz, 1H) ppm. ^13^C NMR (100 MHz, CDCl_3_) *δ* 158.0, 152.6, 143.6, 138.6, 134.9, 130.0, 128.4, 126.0, 125.6, 124.8, 124.0, 123.5, 118.3, 118.0 ppm. HRMS (ESI) *m*/*z* calcd for C_15_H_9_O_2_S [M + H]^+^: 253.0318, found: 253.0313.

### General procedure for synthesis of 6

The synthesis of 6-methyl-14*H*-dibenzo[*a*,*c*]xanthen-14-one (6a) is exemplified. To a stirred solution of 2-chlorobenzoic acid (0.1 mmol) in 1-methyl-2-pyrrolidinone (1.5 mL), was added heterocyclic iodonium (1.2 equiv.), Pd(OAc)_2_ (0.1 equiv.), and K_2_CO_3_ (2.2 equiv.). The reaction mixture was sealed in a tube. The reaction proceeded at 140 °C for 16 h before it was cooled to rt. The reaction mixture was extracted with EtOAc. The combined organic layers were washed with H_2_O and brine, dried over anhydrous Na_2_SO_4_, and evaporated in a *vacuo*. The residue was purified by column chromatography (PE/EtOAc = 20/1–5/1) to provide 6a as a white solid (20 mg, 63% yield), mp 207.5–209.2 °C. ^1^H NMR (400 MHz, CDCl_3_) *δ* 10.08 (dd, *J* = 8.4, 0.9 Hz, 1H), 8.49 (d, *J* = 8.1 Hz, 1H), 8.39 (dd, *J* = 8.0, 1.8 Hz, 2H), 8.26 (s, 1H), 7.69 (dddd, *J* = 12.7, 8.3, 7.1, 1.4 Hz, 2H), 7.62–7.56 (m, 1H), 7.54 (d, *J* = 8.2 Hz, 1H), 7.45–7.40 (m, 1H), 7.38 (d, *J* = 8.3 Hz, 1H), 2.55 (s, 3H) ppm. ^13^C NMR (100 MHz, CDCl_3_) *δ* 178.2, 155.3, 154.3, 141.0, 133.9, 133.6, 129.2, 128.9, 128.4, 127.7, 127.2, 126.7, 126.5, 124.6, 124.0, 123.9, 122.7, 122.3, 121.6, 117.5, 112.0, 22.4 ppm. HRMS (ESI) *m*/*z* calcd for C_22_H_15_O_2_ [M + H]^+^: 311.1067, found: 311.1062.

#### 14*H*-Dibenzo[*a*,*c*]thioxanthen-14-one (6b)

6b (18 mg, 58% yield) was generated following a procedure for the synthesis of 6a as a white solid, mp 198.6–199.4 °C. ^1^H NMR (400 MHz, CDCl_3_) *δ* 9.59–9.43 (m, 1H), 8.67 (d, *J* = 8.3 Hz, 1H), 8.64–8.59 (m, 1H), 8.53 (d, *J* = 7.9 Hz, 1H), 8.46 (d, *J* = 8.2 Hz, 1H), 7.77 (t, *J* = 7.6 Hz, 1H), 7.73–7.59 (m, 5H), 7.54 (t, *J* = 7.5 Hz, 1H) ppm. ^13^C NMR (100 MHz, CDCl_3_) *δ* 182.9, 139.2, 134.1, 132.3, 131.7, 131.6, 130.1, 129.9, 129.5, 129.4, 128.0, 127.7, 127.5, 127.4, 127.3, 127.2, 125.6, 124.9, 124.7, 123.5, 122.6 ppm. HRMS (ESI) *m*/*z* calcd for C_21_H_13_OS [M + H]^+^: 313.0682, found: 313.0671.

#### 9-Fluorodibenzo[*i*,*k*]phenanthridine (6c)

6c (17 mg, 57% yield) was generated following a procedure for the synthesis of 6a as a white solid, mp 151.9–152.5 °C. ^1^H NMR (400 MHz, CDCl_3_) *δ* 10.04 (s, 1H), 8.88 (d, *J* = 8.2 Hz, 1H), 8.82–8.64 (m, 3H), 8.54 (dd, *J* = 11.2, 2.3 Hz, 1H), 8.31 (dd, *J* = 9.0, 6.0 Hz, 1H), 7.86–7.69 (m, 4H), 7.61–7.46 (m, 1H) ppm. ^13^C NMR (100 MHz, CDCl_3_) *δ* 162.6, 160.2, 146.2, 143.7, 132.6, 132.0, 131.9, 130.5, 129.1, 129.0, 128.5, 128.3, 128.1, 127.6, 127.2, 124.0, 123.5, 123.2, 122.6, 118.4, 118.1, 112.1, 111.8 ppm. ^19^F NMR (376 MHz, CDCl_3_) *δ* −112.6 ppm. HRMS (ESI) *m*/*z* calcd for C_21_H_13_FN [M + H]^+^: 298.1027, found: 298.1024.

### General procedure for synthesis of 7

#### 10-(2-(1*H*-Indol-3-yl)ethyl)chromeno[3,2-*b*]indol-11(10*H*)-one (7a)

7a (48 mg, 63% yield) was generated following a procedure for the synthesis of 4l as a white solid, mp 157.5–158.6 °C. ^1^H NMR (400 MHz, CDCl_3_) *δ* 8.55–8.45 (m, 1H), 8.01 (d, *J* = 8.1 Hz, 1H), 7.97 (s, 1H), 7.81 (d, *J* = 7.0 Hz, 1H), 7.76–7.66 (m, 2H), 7.50–7.44 (m, 1H), 7.43–7.38 (m, 1H), 7.35 (d, *J* = 7.0 Hz, 1H), 7.29 (d, *J* = 8.6 Hz, 1H), 7.25–7.16 (m, 3H), 6.97 (d, *J* = 2.0 Hz, 1H), 5.09–4.96 (m, 2H), 3.43–3.25 (m, 2H) ppm. ^13^C NMR (100 MHz, CDCl_3_) *δ* 170.3, 155.6, 145.2, 138.1, 136.4, 132.8, 128.1, 127.6, 126.3, 124.4, 124.0, 122.5, 122.2, 120.2, 120.1, 119.7, 118.9, 118.2, 114.9, 112.8, 111.3, 110.4, 100.1, 45.8, 27.1 ppm. HRMS (ESI) *m*/*z* calcd for C_25_H_19_N_2_O_2_ [M + H]^+^: 379.1441, found: 379.1442.

#### 10-((3*R*)-10,13-Dimethyl-17-oxohexadecahydro-1*H*-cyclopenta[*a*]phenanthren-3-yl)chromeno[3,2-*b*]indol-11(10*H*)-one (7b)

7b (57 mg, 56% yield) was generated following a procedure for the synthesis of 4l as a white solid, mp 167.5–168.6 °C. ^1^H NMR (400 MHz, CDCl_3_) *δ* 8.47 (d, *J* = 7.6 Hz, 1H), 8.07 (d, *J* = 7.5 Hz, 1H), 7.70 (dd, *J* = 14.6, 7.1 Hz, 2H), 7.61 (d, *J* = 8.2 Hz, 1H), 7.51 (s, 1H), 7.45 (s, 1H), 7.27 (d, *J* = 9.2 Hz, 1H), 5.99 (d, *J* = 6.3 Hz, 1H), 2.76–2.64 (m, 1H), 2.48 (dd, *J* = 19.2, 8.4 Hz, 1H), 2.27 (s, 1H), 2.10 (dd, *J* = 19.3, 9.6 Hz, 1H), 2.05–1.91 (m, 3H), 1.84 (t, *J* = 14.4 Hz, 2H), 1.71 (s, 5H), 1.51 (dt, *J* = 26.0, 13.5 Hz, 5H), 1.30 (d, *J* = 8.6 Hz, 4H), 1.12 (s, 3H), 1.07 (s, 1H), 0.93 (s, 3H), 0.89 (d, *J* = 11.4 Hz, 2H) ppm. ^13^C NMR (100 MHz, CDCl_3_) *δ* 170.2, 155.2, 145.5, 136.9, 132.8, 127.8, 126.4, 124.5, 123.9, 120.5, 120.3, 120.0, 118.0, 115.7, 112.5, 56.3, 51.6, 50.4, 48.1, 39.6, 36.9, 36.0, 35.6, 34.4, 33.0, 31.9, 30.6, 28.4, 26.3, 21.9, 20.8, 17.4, 14.0 ppm. ^13^C NMR (100 MHz, dept 90, CDCl_3_) *δ* 132.7, 127.7, 126.3, 123.8, 120.4, 120.0, 117.9, 112.4, 56.2, 51.4, 50.3, 39.5, 35.5 ppm. ^13^C NMR (100 MHz, dept 135, CDCl_3_) *δ* 132.7, 127.7, 126.3, 123.8, 120.4, 119.9, 117.9, 112.4, 56.2, 51.4, 50.3, 39.5, 36.8, 35.9, 35.5, 32.9, 31.8, 30.5, 28.3, 26.2, 21.8, 20.7, 17.3, 13.9 ppm. HRMS (ESI) *m*/*z* calcd for C_34_H_38_NO_3_ [M + H]^+^: 508.2846, found: 508.2855.

#### 10-(2-Aminophenyl)chromeno[3,2-*b*]indol-11(10*H*)-one (8a)

8a (43 mg, 66% yield) was generated following a procedure for the synthesis of 3a as a pink solid, mp 215.5–216.8 °C. ^1^H NMR (500 MHz, CDCl_3_) *δ* 8.33 (d, *J* = 7.2 Hz, 1H), 8.09 (d, *J* = 8.0 Hz, 1H), 7.77–7.66 (m, 2H), 7.49 (t, *J* = 7.6 Hz, 1H), 7.40 (dd, *J* = 14.6, 6.9 Hz, 1H), 7.34 (dd, *J* = 9.3, 5.4 Hz, 2H), 7.23 (d, *J* = 8.5 Hz, 1H), 7.19 (d, *J* = 7.6 Hz, 1H), 7.01 (d, *J* = 6.8 Hz, 1H), 6.91 (s, 1H), 2.71 (s, 2H). ppm. ^13^C NMR (100 MHz, CDCl_3_) *δ* 169.3, 155.8, 146.3, 139.0, 133.0, 130.0, 129.6, 128.9, 126.5, 124.6, 124.2, 121.5, 120.1, 119.1, 118.2, 116.9, 115.9, 112.1 ppm. HRMS (ESI) *m*/*z* calcd for C_21_H_15_N_2_O_2_ [M + H]^+^: 327.1128, found: 327.1127.

#### 10-(2-Aminophenyl)-3-methoxychromeno[3,2-*b*]indol-11(10*H*)-one (8b)

8b (48 mg, 68% yield) was generated following a procedure for the synthesis of 3a as a white solid, mp 224.2–226.1 °C. ^1^H NMR (400 MHz, CDCl_3_) *δ* 8.38 (d, *J* = 8.8 Hz, 1H), 8.20 (d, *J* = 8.0 Hz, 1H), 7.62 (t, *J* = 7.6 Hz, 1H), 7.47 (t, *J* = 7.6 Hz, 2H), 7.41 (s, 1H), 7.35 (dd, *J* = 12.6, 8.3 Hz, 2H), 7.13 (d, *J* = 8.9 Hz, 1H), 7.09 (d, *J* = 8.0 Hz, 1H), 7.04 (t, *J* = 7.5 Hz, 1H), 4.11 (s, 3H). ppm. ^13^C NMR (100 MHz, CDCl_3_) *δ* 169.1, 163.7, 157.5, 145.9, 144.2, 138.6, 130.0, 129.6, 128.5, 127.7, 123.5, 121.3, 121.0, 119.8, 118.9, 118.4, 116.7, 115.9, 113.4, 112.0, 100.6, 56.0 ppm. HRMS (ESI) *m*/*z* calcd for C_22_H_17_N_2_O_3_ [M + H]^+^: 357.1234, found: 357.1237.

### Procedure for the synthesis of 9a

To a stirred solution of 8a (40 mg) in dichloromethane (3 mL), was added acetyl chloride (1.2 equiv.) and triethylamine (2.0 equiv.). The reaction proceeded at rt for 4 h before dichloromethane was removed by a rotary evaporation. The reaction mixture was extracted with EtOAc. The combined organic layers were washed with H_2_O and brine, dried over anhydrous Na_2_SO_4_, and evaporated in a *vacuo*. The residue was purified by column chromatography (PE/EtOAc = 10/1–5/1) to provide *N*-(2-(11-oxochromeno[3,2-*b*] indol-10(11*H*)-yl)phenyl) acetamide (42 mg) as a yellow solid. Then, to this obtained solid was added polyphosphoric acid (0.2 mL) and POCl_3_ (10 equiv.). The reaction proceeded in a sealed tube at 120 °C for 3 h. The reaction mixture was neutralized with NaHCO_3_ (Sat.) and extracted with EtOAc. The combined organic layers were washed with H_2_O and brine, dried over anhydrous Na_2_SO_4_, and evaporated in a *vacuo*. The residue was purified by column chromatography (PE/EtOAc = 10/1–3/1) to provide 6-methyl-15*H*-benzo[2,3][1,4] diazepino[6,7,1-*h*,*i*]chromeno [3,2-*b*]indol-15-one 9a as a yellow solid (33 mg, 51% yield over two steps), mp 198.2–199.4 °C. ^1^H NMR (400 MHz, CDCl_3_) *δ* 8.47 (dd, *J* = 8.0, 1.5 Hz, 1H), 7.93 (d, *J* = 7.8 Hz, 1H), 7.81–7.74 (m, 1H), 7.69 (d, *J* = 8.4 Hz, 1H), 7.54 (d, *J* = 7.3 Hz, 1H), 7.53–7.48 (m, 1H), 7.33 (t, *J* = 7.8 Hz, 2H), 7.17–7.10 (m, 2H), 6.75–6.65 (m, 1H), 2.60 (s, 3H) ppm. ^13^C NMR (100 MHz, CDCl_3_) *δ* 169.4, 167.1, 155.5, 151.9, 151.0, 139.7, 135.2, 133.5, 129.5, 129.4, 127.5, 126.9, 125.9, 125.8, 124.9, 124.6, 124.4, 123.8, 122.6, 122.2, 119.8, 118.1, 28.5 ppm. HRMS (ESI) *m*/*z* calcd for C_23_H_15_N_2_O_2_ [M + H]^+^: 351.1128, found: 351.1127.

### Procedure for the synthesis of 9b

To a stirred solution of 8b (45 mg) in EtOH (4 mL), was added TsOH·H_2_O (0.1 equiv.). The reaction proceeded at a reflux overnight before EtOH was removed by a rotary evaporation. The reaction mixture was extracted with EtOAc. The combined organic layers were washed with H_2_O and brine, dried over anhydrous Na_2_SO_4_, and evaporated in a *vacuo*. The residue was purified by column chromatography (PE/EtOAc = 10/1–5/1) to provide 8-methoxy-10-oxa-5,14*b*-diazaindeno [1,2,3-*g*,*h*]tetraphene 9b as a yellow solid (41 mg, 95% yield), mp 173.5–174.4 °C. ^1^H NMR (400 MHz, CDCl_3_) *δ* 8.33 (d, *J* = 8.0 Hz, 1H), 8.24 (d, *J* = 8.6 Hz, 1H), 8.02 (dd, *J* = 17.2, 8.0 Hz, 2H), 7.75 (d, *J* = 7.3 Hz, 1H), 7.58 (t, *J* = 7.8 Hz, 1H), 7.44 (t, *J* = 7.5 Hz, 1H), 7.32 (t, *J* = 7.6 Hz, 1H), 7.27–7.21 (m, 1H), 6.92 (d, *J* = 10.1 Hz, 2H), 3.93 (s, 3H) ppm. ^13^C NMR (100 MHz, CDCl_3_) *δ* 163.3, 158.1, 148.0, 139.5, 132.8, 131.2, 130.3, 129.1, 126.3, 125.8, 125.1, 124.6, 121.6, 119.2, 118.1, 116.7, 114.8, 114.0, 113.6, 112.4, 102.2, 55.8 ppm. HRMS (ESI) *m*/*z* calcd for C_22_H_15_N_2_O_2_ [M + H]^+^: 339.1128, found: 339.1122.

### The general synthesis of heterocyclic iodoniums 1

All the synthetic heterocyclic idoniums are reported in our previous work, and they are prepared conveniently using reported procedure.^10,18^

#### 11-Oxo-11*H*-benzo[*b*]chromeno[2,3-*d*]iodol-10-ium triflate (1a)

^1^H NMR (400 MHz, DMSO) *δ* 8.50–8.35 (m, 2H), 8.22 (dd, *J* = 7.9, 1.4 Hz, 1H), 8.11–8.02 (m, 2H), 8.01–7.91 (m, 2H), 7.72 (t, *J* = 7.5 Hz, 1H) ppm. ^13^C NMR (100 MHz, DMSO) *δ* 172.3, 164.1, 155.2, 135.9, 135.2, 134.7, 131.4, 131.3, 128.9, 127.0, 125.2, 122.5, 119.6, 118.8, 108.4 ppm.

#### 8-Methyl-11-oxo-10λ^3^-benzo[*b*]chromeno[2,3-*d*]iodol-10(11*H*)-yl trifluoromethanesulfonate (1b)

^1^H NMR (400 MHz, DMSO) *δ* 8.31 (d, *J* = 8.0 Hz, 1H), 8.23 (s, 1H), 8.21 (dd, *J* = 8.0, 1.5 Hz, 1H), 8.03 (ddd, *J* = 8.6, 7.1, 1.7 Hz, 1H), 7.95 (d, *J* = 7.9 Hz, 1H), 7.88 (d, *J* = 7.7 Hz, 1H), 7.74–7.68 (m, 1H), 2.58 (s, 3H) ppm. ^13^C NMR (100 MHz, DMSO) *δ* 172.2, 164.0, 155.1, 146.2, 135.8, 132.5, 132.2, 131.2, 128.5, 126.9, 125.2, 122.4, 119.7, 118.7, 107.4, 21.8 ppm.

#### 8-Chloro-11-oxo-10λ^3^-benzo[*b*]chromeno[2,3-*d*]iodol-10(11*H*)-yl trifluoromethanesulfonate (1c)

^1^H NMR (400 MHz, DMSO) *δ* 8.42 (d, *J* = 1.8 Hz, 1H), 8.39 (d, *J* = 8.4 Hz, 1H), 8.21 (dd, *J* = 7.9, 1.2 Hz, 1H), 8.14 (dd, *J* = 8.4, 1.8 Hz, 1H), 8.07–8.00 (m, 1H), 7.95 (d, *J* = 8.3 Hz, 1H), 7.71 (t, *J* = 7.5 Hz, 1H) ppm. ^13^C NMR (100 MHz, DMSO) *δ* 172.2, 163.2, 155.1, 138.5, 136.0, 134.3, 131.9, 131.3, 130.9, 129.8, 128.5, 127.1, 125.3, 122.5, 120.1, 118.8, 109.1 ppm.

#### 8-Fluoro-11-oxo-10λ^3^-benzo[*b*]chromeno[2,3-*d*]iodol-10(11*H*)-yl trifluoromethanesulfonate (1d)

^1^H NMR (400 MHz, DMSO) *δ* 8.50–8.44 (m, 1H), 8.22 (ddd, *J* = 8.0, 5.9, 2.3 Hz, 2H), 8.09–8.00 (m, 1H), 8.01–7.91 (m, 2H), 7.71 (dd, *J* = 10.6, 4.3 Hz, 1H) ppm. ^13^C NMR (100 MHz, DMSO) *δ* 172.1, 165.2, 163.2, 162.7, 155.2, 136.0, 132.1, 130.8, 130.7, 127.1, 125.3, 122.5, 120.3, 120.2, 112.0, 119.7, 119.3, 119.0, 118.8, 108.5 ppm.

#### 7-Fluoro-11-oxo-10λ^3^-benzo[*b*]chromeno[2,3-*d*]iodol-10(11*H*)-yl trifluoromethanesulfonate (1e)

^1^H NMR (400 MHz, DMSO) *δ* 8.50–8.43 (m, 1H), 8.39 (dd, *J* = 5.6, 2.7 Hz, 1H), 8.23 (d, *J* = 7.9 Hz, 1H), 8.06 (t, *J* = 7.5 Hz, 1H), 7.95 (dd, *J* = 8.2, 2.3 Hz, 1H), 7.87 (ddd, *J* = 8.9, 6.2, 2.7 Hz, 1H), 7.73 (t, *J* = 6.2 Hz, 1H) ppm. ^13^C NMR (100 MHz, DMSO) *δ* 172.3, 164.9, 163.0, 162.4, 155.1, 137.5, 137.4, 136.1, 133.7, 133.6, 127.1, 125.3, 122.4, 122.3, 122.0, 118.8, 115.9, 115.7, 113.6, 109.7 ppm.

#### 11-Oxo-10λ^3^-benzo[*b*]thiochromeno[2,3-*d*]iodol-10(11*H*)-yl trifluoromethanesulfonate (1f)

^1^H NMR (400 MHz, DMSO) *δ* 8.46 (t, *J* = 9.1 Hz, 2H), 8.34 (d, *J* = 7.8 Hz, 1H), 8.22 (d, *J* = 8.1 Hz, 1H), 8.04 (d, *J* = 7.6 Hz, 1H), 8.01–7.96 (m, 1H), 7.92 (t, *J* = 7.9 Hz, 1H), 7.87 (t, *J* = 7.6 Hz, 1H) ppm. ^13^C NMR (100 MHz, DMSO) *δ* 174.9, 150.6, 143.4, 135.8, 134.0, 132.1, 131.5, 129.7, 128.9, 128.8, 128.1, 128.0, 123.3, 122.8 ppm.

#### 7*H*-7λ^3^-Benzo[4,5]iodolo[2,3-*c*]quinolin-7-yl trifluoromethanesulfonate (1g)

To a stirred solution of 3-iodo-4-phenylquinoline 1g–l (2.0 g, 6.04 mol) in anhydrous DCM (20 mL) was added TfOH (1.60 mL, 3.0 equiv.) and followed by the slow addition of *m*-CPBA (85%, 1.84 g, 1.5 equiv.). The solution was stirred for 2 h at rt before DCM was removed by rotary evaporation. Et_2_O (20 mL) was added to the remained solid. The mixture was stirred for 30 min, and then filtered. The obtained solid was washed with Et_2_O three times and dried in high *vacuo* to provide 1g (2.43 g, 84% yield) as a yellow solid. ^1^H NMR (400 MHz, DMSO) *δ* 9.53 (s, 1H), 9.14 (d, *J* = 7.9 Hz, 1H), 9.09 (d, *J* = 8.8 Hz, 1H), 8.45 (d, *J* = 7.6 Hz, 1H), 8.30 (d, *J* = 7.9 Hz, 1H), 8.03 (t, *J* = 7.6 Hz, 2H), 7.97–7.86 (m, 2H) ppm. ^13^C NMR (100 MHz, DMSO) *δ* 148.6, 147.9, 144.8, 141.2, 132.4, 132.3, 131.2, 131.1, 131.1, 131.0, 130.6, 129.1, 126.4, 124.2, 123.9, 117.7 ppm.

#### 9-Methyl-7*H*-7λ^3^-benzo[4,5]iodolo[2,3-*c*]quinolin-7-yl trifluoromethanesulfonate (1h)

^1^H NMR (400 MHz, DMSO) *δ* 9.48 (s, 1H), 9.04 (d, *J* = 8.4 Hz, 1H), 8.99 (d, *J* = 8.4 Hz, 1H), 8.28 (dd, *J* = 8.4, 1.0 Hz, 1H), 8.20 (s, 1H), 8.02 (dd, *J* = 11.3, 4.0 Hz, 1H), 7.95–7.88 (m, 1H), 7.81 (d, *J* = 7.5 Hz, 1H), 2.56 (s, 3H) ppm. ^13^C NMR (100 MHz, DMSO) *δ* 148.4, 147.7, 144.8, 143.2, 138.4, 132.0, 131.9, 131.1, 131.0, 130.5, 129.0, 126.2, 124.2, 124.0, 122.4, 119.1, 117.0, 21.2 ppm.

#### 9-Chloro-7*H*-7λ^3^-benzo[4,5]iodolo[2,3-*c*]quinolin-7-yl trifluoromethanesulfonate (1i)

^1^H NMR (400 MHz, DMSO) *δ* 9.46 (s, 1H), 9.03 (d, *J* = 8.9 Hz, 1H), 8.94 (d, *J* = 8.5 Hz, 1H), 8.38 (d, *J* = 2.2 Hz, 1H), 8.27 (dd, *J* = 8.4, 1.1 Hz, 1H), 8.05–7.97 (m, 2H), 7.90 (ddd, *J* = 8.4, 7.0, 1.3 Hz, 1H) ppm. ^13^C NMR (100 MHz, DMSO) *δ* 148.4, 147.9, 143.8, 140.1, 136.2, 133.2, 131.4, 130.7, 130.5, 129.3, 126.2, 124.9, 124.1, 122.4, 119.1, 118.2 ppm.

#### 2-Fluoro-7*H*-7λ^3^-benzo[4,5]iodolo[2,3-*c*]quinolin-7-yl trifluoromethanesulfonate (1j)

^1^H NMR (400 MHz, DMSO) *δ* 9.54 (d, *J* = 3.4 Hz, 1H), 9.15 (dd, *J* = 7.7, 2.7 Hz, 1H), 8.89–8.72 (m, 1H), 8.46 (dd, *J* = 8.1, 2.3 Hz, 1H), 8.40 (ddd, *J* = 9.3, 6.0, 3.4 Hz, 1H), 8.08–7.85 (m, 3H) ppm. ^13^C NMR (100 MHz, DMSO) *δ* 162.4, 159.9, 148.0, 145.3, 144.5, 144.4, 140.7, 133.7, 133.6, 132.4, 132.2, 131.2, 131.2, 127.0, 126.9, 123.7, 122.3, 121.2, 121.0, 119.1, 118.9, 108.8, 108.6 ppm.

#### Benzo[4,5]iodolo[3,2-*c*]isoquinolin-11-ium triflate (1k)

^1^H NMR (400 MHz, DMSO) *δ* 9.73 (s, 1H), 8.59–8.51 (m, 2H), 8.43 (dd, *J* = 10.6, 8.3 Hz, 2H), 8.18–8.07 (m, 1H), 7.97 (t, *J* = 7.5 Hz, 2H), 7.90–7.81 (m, 1H) ppm. ^13^C NMR (100 MHz, DMSO) *δ* 155.6, 153.4, 140.6, 134.5, 133.6, 132.6, 131.1, 130.7, 130.1, 129.5, 129.3, 126.7, 122.3, 121.4, 120.5 ppm.

#### 9-Methyl-11*H*-11λ^3^-benzo[4,5]iodolo[3,2-*c*]isoquinolin-11-yl trifluoromethanesulfonate (1l)

^1^H NMR (400 MHz, DMSO) *δ* 9.58 (s, 1H), 8.40 (d, *J* = 8.2 Hz, 1H), 8.34 (d, *J* = 8.1 Hz, 1H), 8.24 (d, *J* = 7.9 Hz, 1H), 8.07 (s, 1H), 8.06–8.01 (m, 1H), 7.89 (t, *J* = 7.3 Hz, 1H), 7.66 (d, *J* = 7.8 Hz, 1H), 2.51 (s, 3H) ppm. ^13^C NMR (100 MHz, DMSO) *δ* 155.4, 153.2, 143.2, 137.8, 134.3, 133.5, 132.0, 130.2, 129.8, 129.4, 129.2, 128.6, 126.4, 121.2, 119.5, 21.5 ppm.

#### 6-Oxo-7λ^3^-benzo[*b*]chromeno[4,3-*d*]iodol-7(6*H*)-yl trifluoromethanesulfonate (1m)

^1^H NMR (400 MHz, DMSO) *δ* 9.12–9.02 (m, 1H), 8.81 (d, *J* = 7.5 Hz, 1H), 8.53 (dd, *J* = 8.3, 1.0 Hz, 1H), 8.10–8.01 (m, 1H), 8.00–7.93 (m, 1H), 7.92–7.85 (m, 1H), 7.71 (dd, *J* = 8.3, 0.9 Hz, 1H), 7.66–7.56 (m, 1H) ppm. ^13^C NMR (100 MHz, DMSO) *δ* 157.2, 153.6, 152.7, 141.0, 133.5, 133.4, 133.0, 132.2, 131.4, 126.2, 125.6, 125.2, 118.2, 117.9, 117.3 ppm.

## Conflicts of interest

There are no conflicts to declare.

## Supplementary Material

RA-009-C9RA07288H-s001
