# Supplementary material for: Heterocyclic iodoniums as versatile synthons to approach diversified polycyclic heteroarenes
Source: RSC Adv. 2019 Oct 16;9(57):33170–9. doi: 10.1039/c9ra07288h (PMC9073335; doi:10.1039/c9ra07288h)
Supplement: RA-009-C9RA07288H-s001 [file RA-009-C9RA07288H-s001.pdf]

Electronic Supplementary Material (ESI) for RSC Advances.
This journal is © The Royal Society of Chemistry 2019

Heterocyclic Iodoniums as Versatile Synthons to Approach Diversified Polycyclic Heteroarenes

Daqian Zhu,[ab] Zhouming Wu,[a] Liyun Liang,[a] Yameng Sun,[a] Bingling Luo,[a] Peng Huang,[a] Shijun Wen*[a]

[a]State Key Laboratory of Oncology in South China, Collaborative Innovation Center for Cancer Medicine, Sun Yat-sen University Cancer Center, 651 Dongfeng East Road, Guangzhou 510060.

[b]School of pharmacy, Guangdong Pharmaceutical University, 280 Waihuan East Road, Guangzhou 510006, China

Contents

Studies on the reaction of **1a** and *p*-anisidine...S1
NMR Spectra S2

Table S1. Studies on the reaction of **1a** and *p*-anisidine

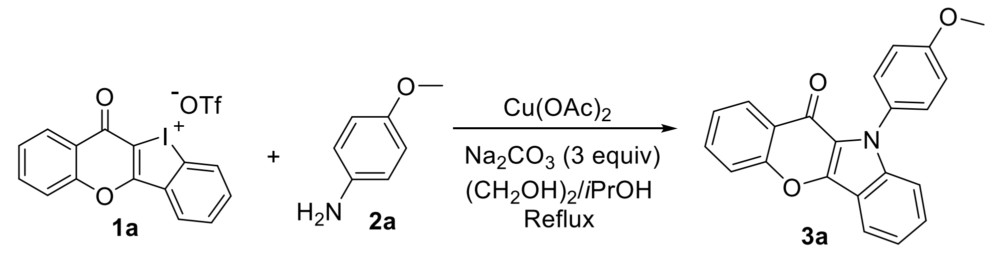

Entry	**2a** (equiv)	$Cu(OAc)_2$ (equiv)	Yield (%)
1	1.5	0.2	32
2	2.5	0.2	86
3	3	0.2	87
4	4	0.2	90
5	2.5	0.1	84

Synthesize heterocycle-fused indoles with heterocyclic iodonium **1a** and *p*-anisidine. Reaction conditions: **1a** (0.1 mmol), *p*-anisidine (2.5 equiv), *i*-PrOH/$(CH_2OH)_2$ (0.9/0.1mL), refluxing, Ar, 16 h.

NMR Spectra

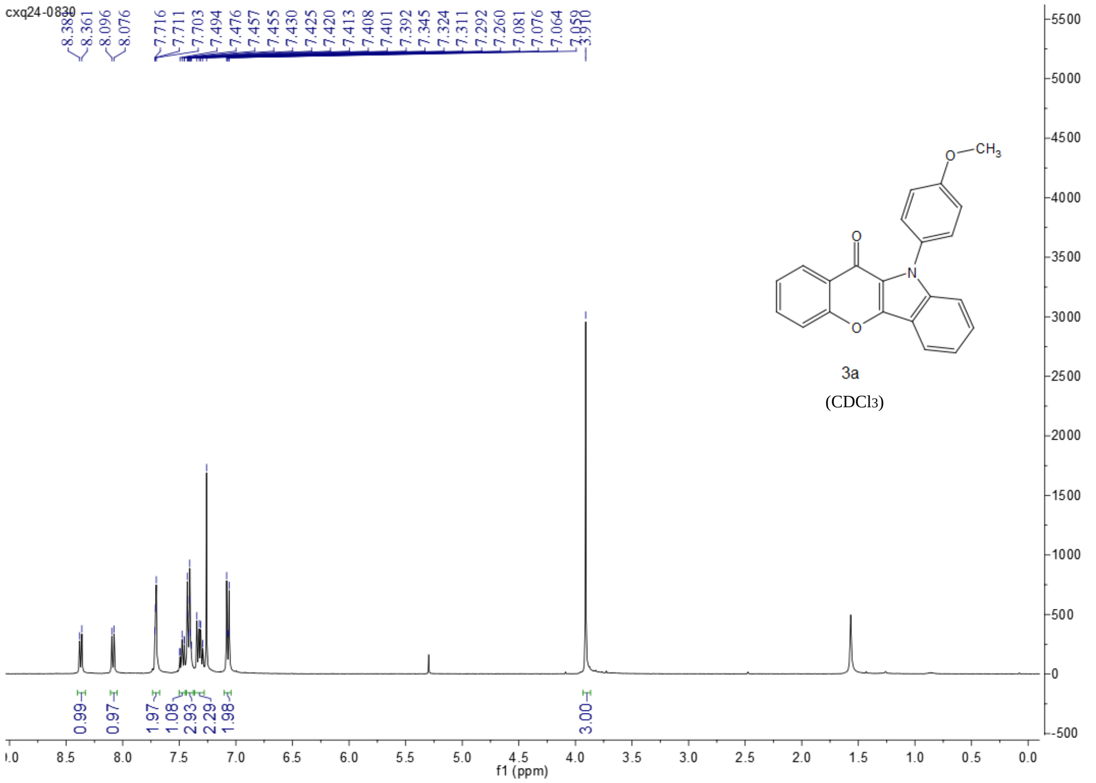

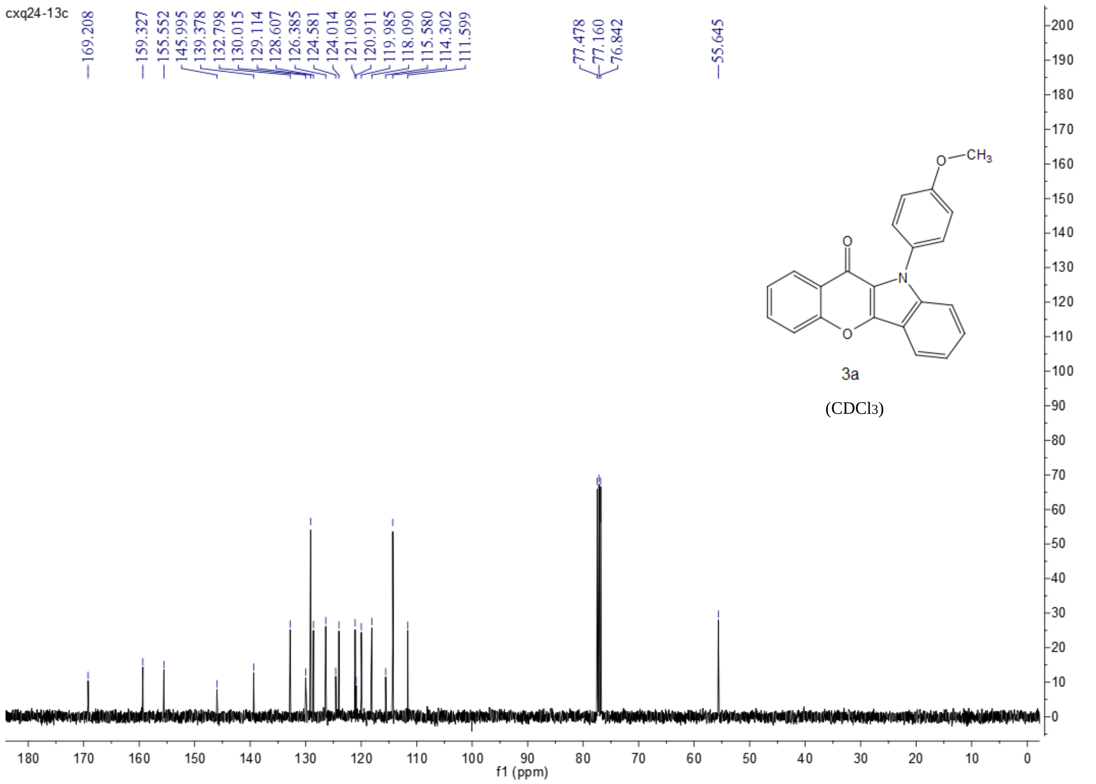

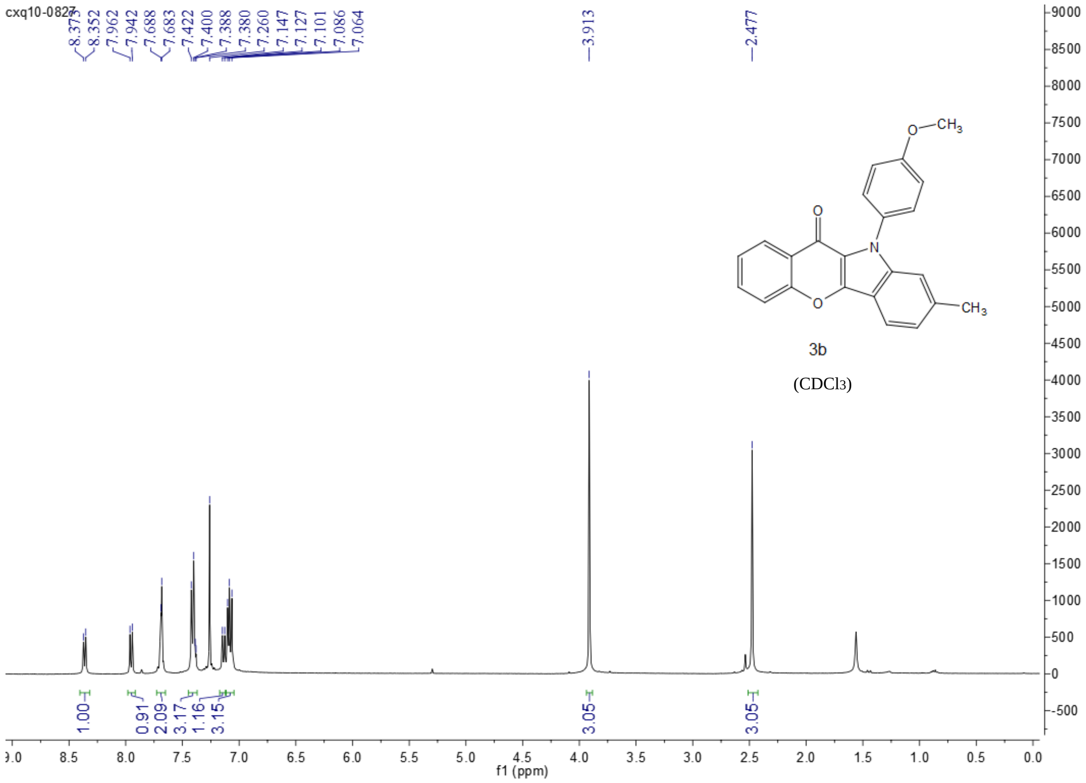
cxq10-0827
8.373
8.352
7.962
7.942
7.688
7.683
7.422
7.400
7.388
7.380
7.260
7.147
7.127
7.101
7.086
7.064
3.913
2.477
O
N
O
CH3
CH3
3b
(CDCl3)
1.00
0.91
2.09
3.17
1.16
3.15
3.05
3.05
f1 (ppm)

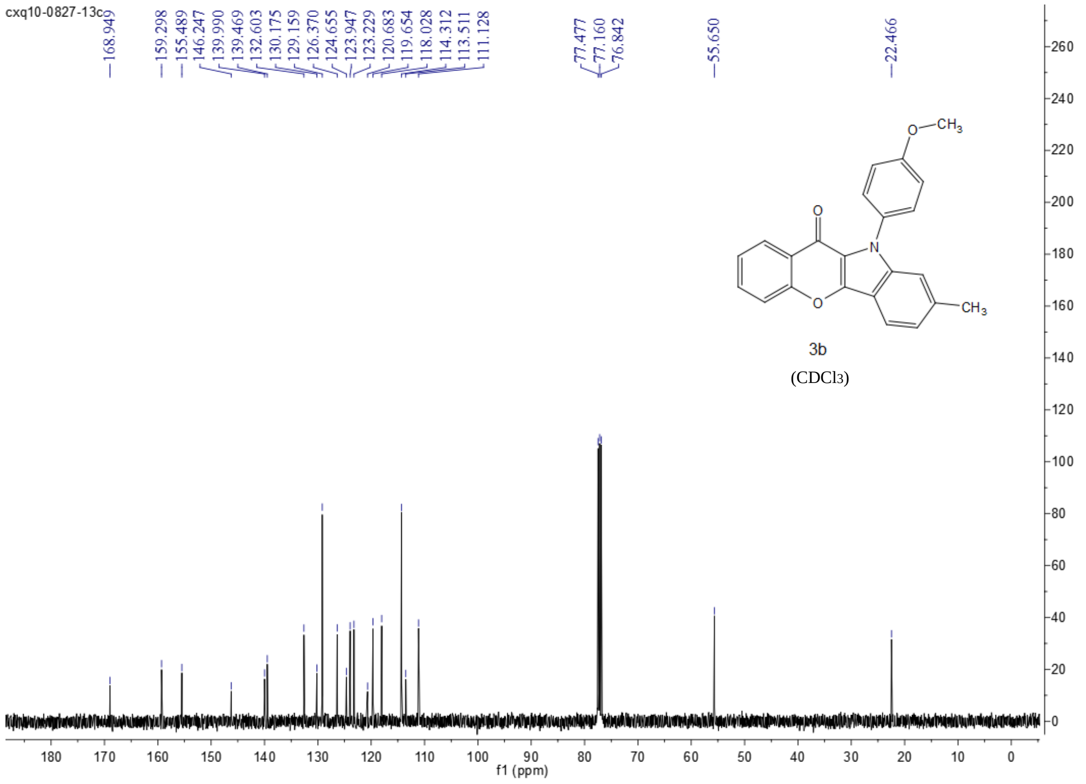
cxq10-0827-13c
168.949
159.298
155.489
146.247
139.990
139.469
132.603
130.175
129.159
126.370
124.655
123.947
123.229
120.683
119.654
118.028
114.312
113.511
111.128
77.477
77.160
76.842
55.650
22.466
O
N
O
CH3
CH3
3b
(CDCl3)
f1 (ppm)

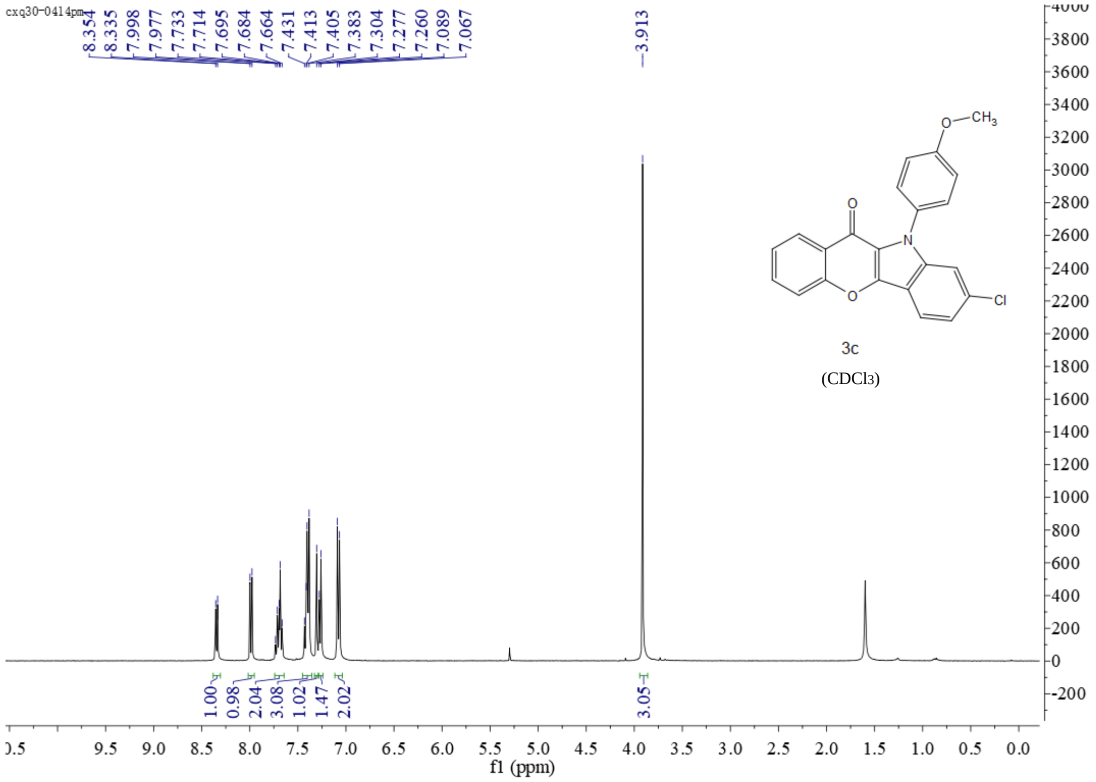
cxq30-0414pm
8.354
8.335
7.998
7.977
7.733
7.714
7.695
7.684
7.664
7.431
7.413
7.405
7.383
7.304
7.277
7.260
7.089
7.067
3.913
1.00
0.98
2.04
3.08
1.02
1.47
2.02
3.05
3c
(CDCl3)
f1 (ppm)

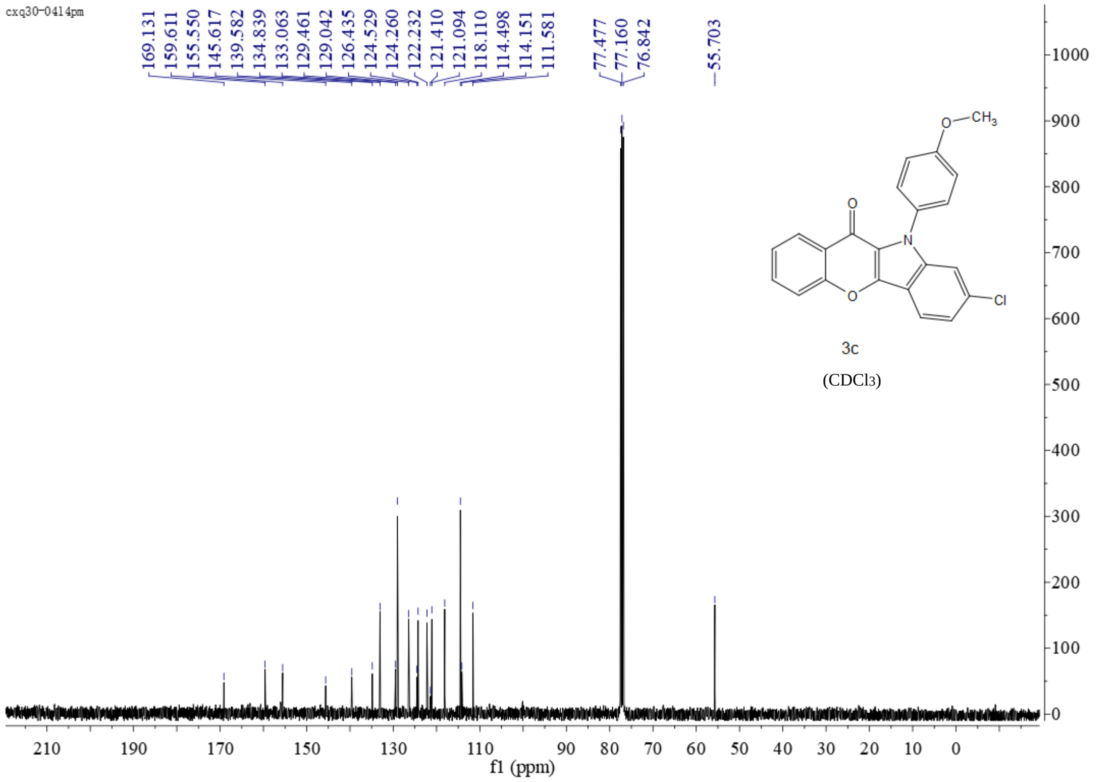
cxq30-0414pm
169.131
159.611
155.550
145.617
139.582
134.839
133.063
129.461
129.042
126.435
124.529
124.260
122.232
121.410
121.094
118.110
114.498
114.151
111.581
77.477
77.160
76.842
55.703
3c
(CDCl3)
f1 (ppm)

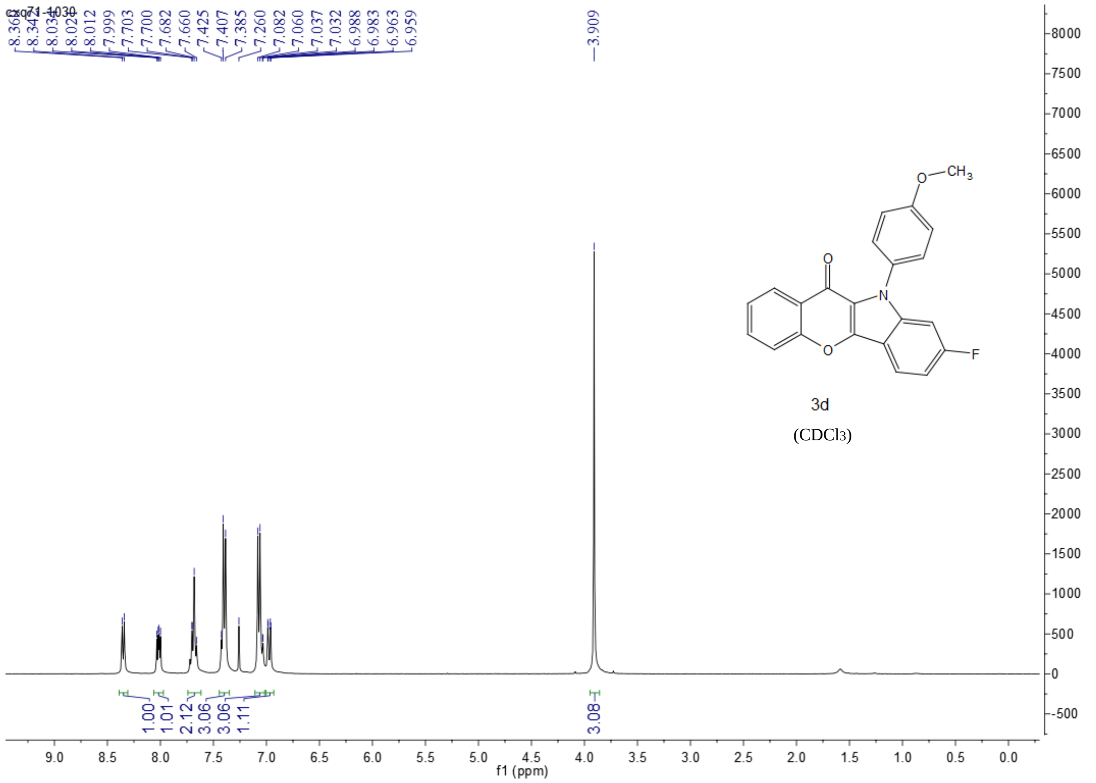
cxq71-1030
3d
(CDCl3)

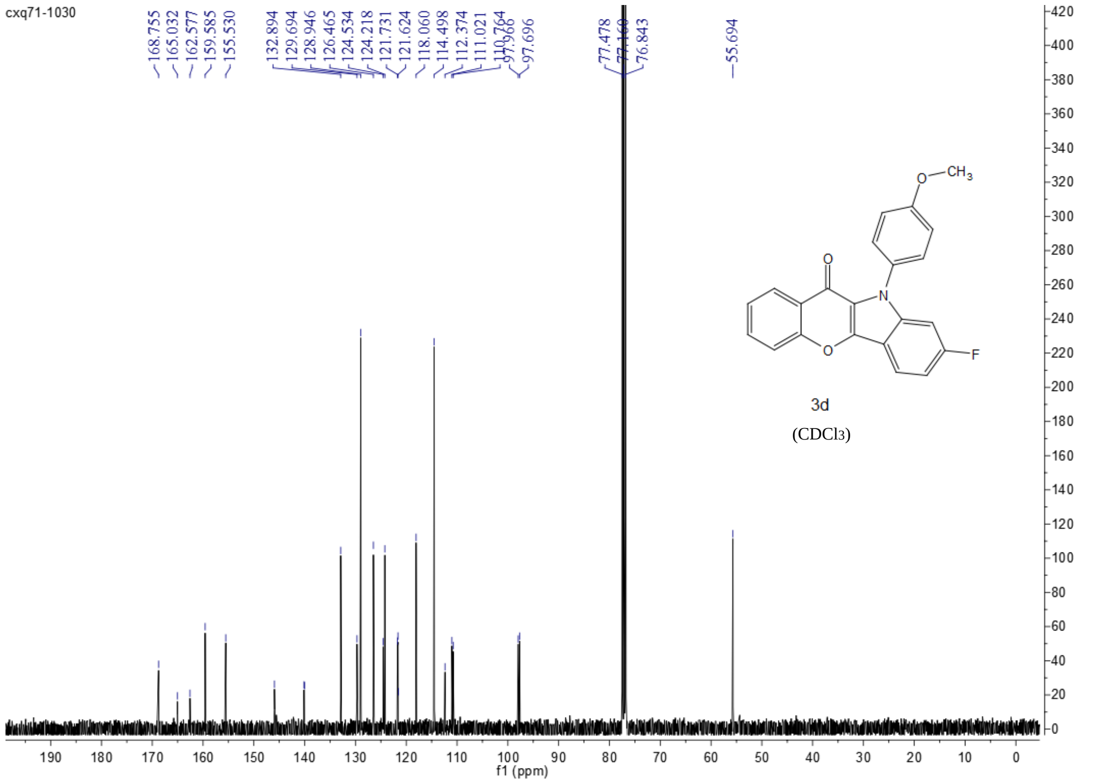
cxq71-1030
3d
(CDCl3)

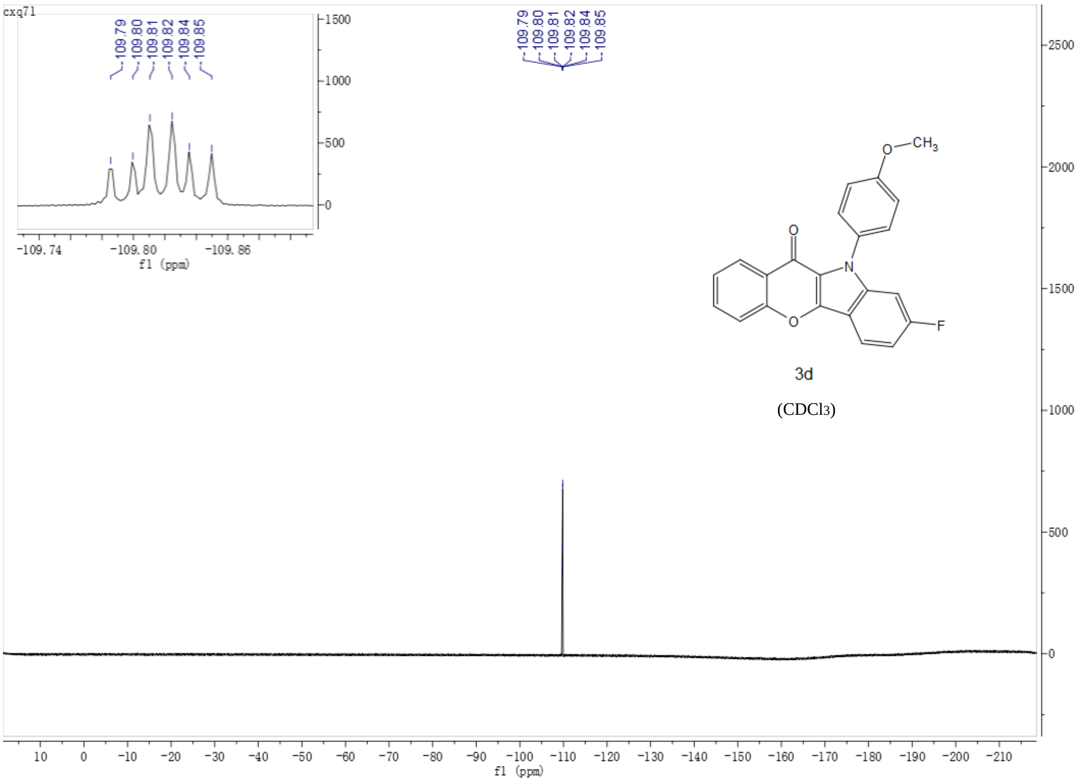

3d

(CDCl3)

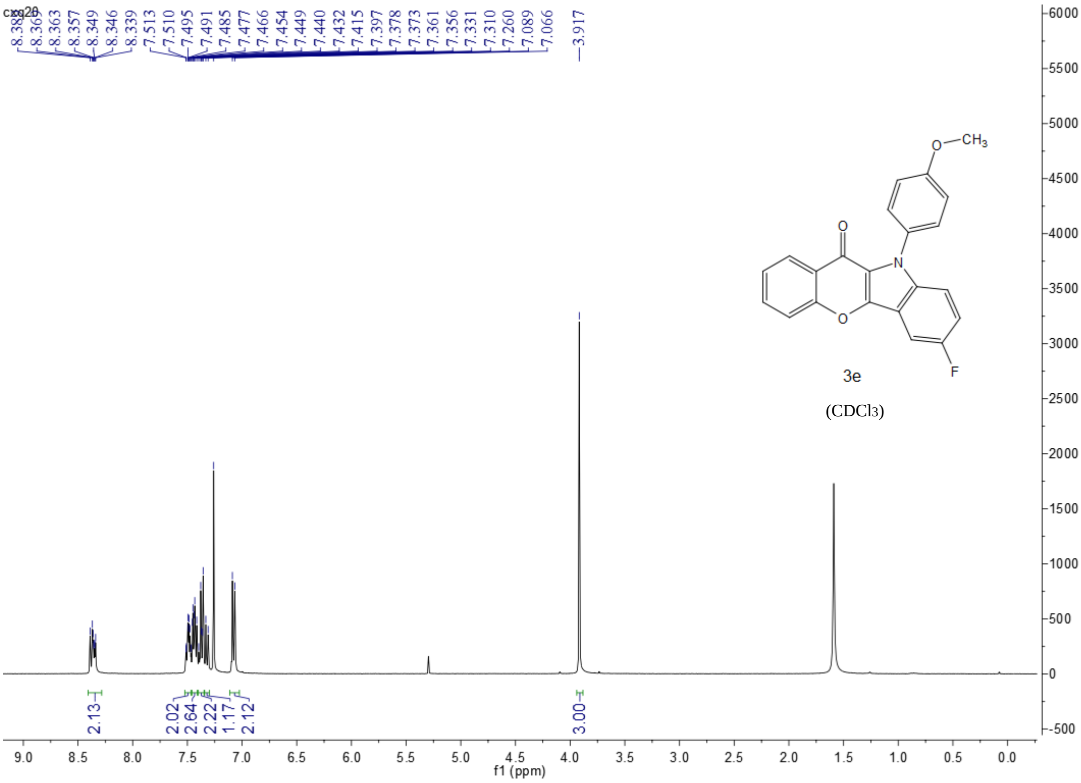

3e

(CDCl3)

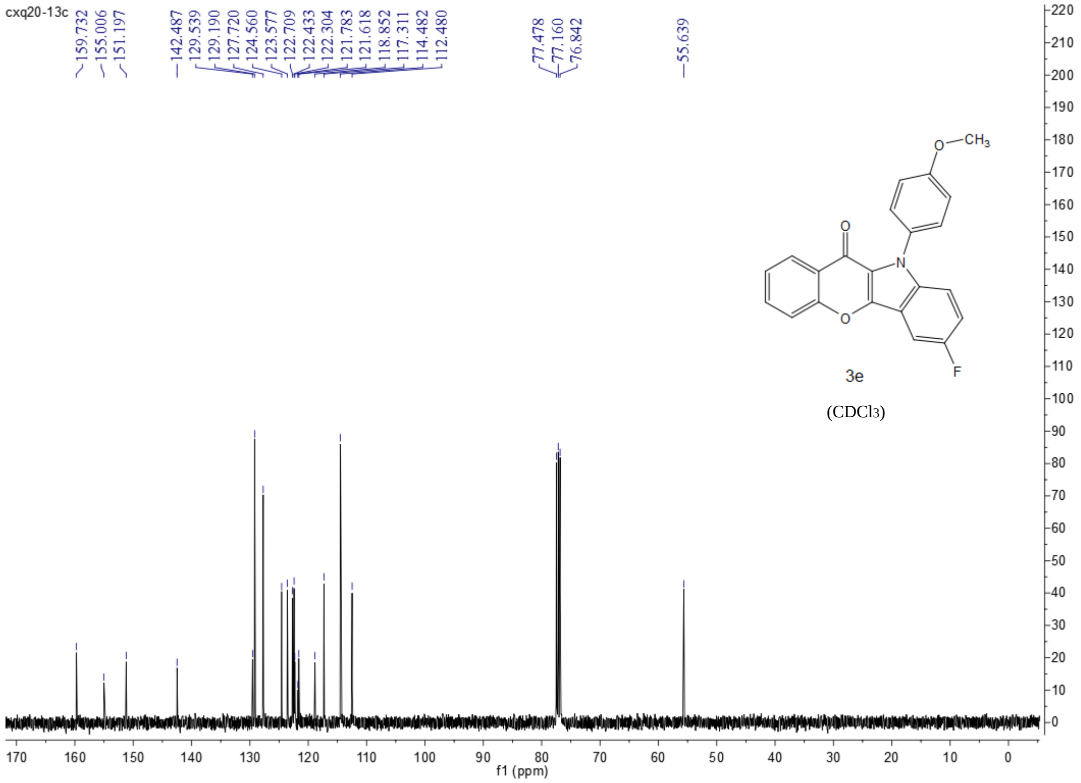

3e

(CDCl3)

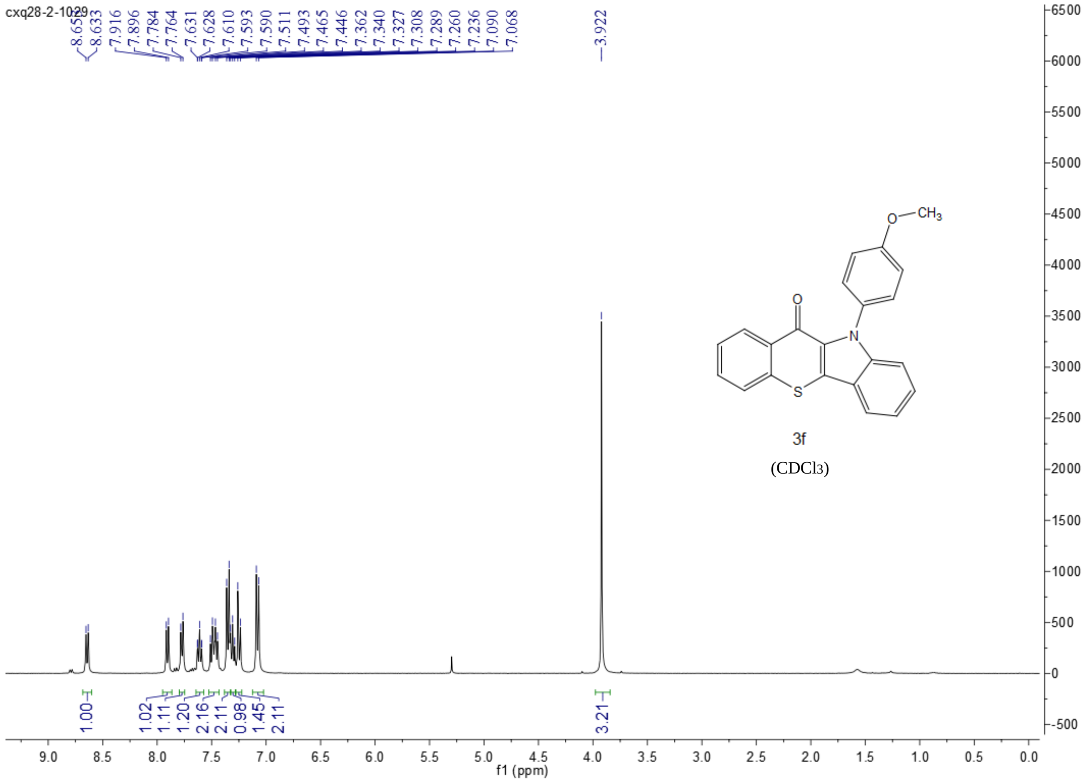

3f

(CDCl3)

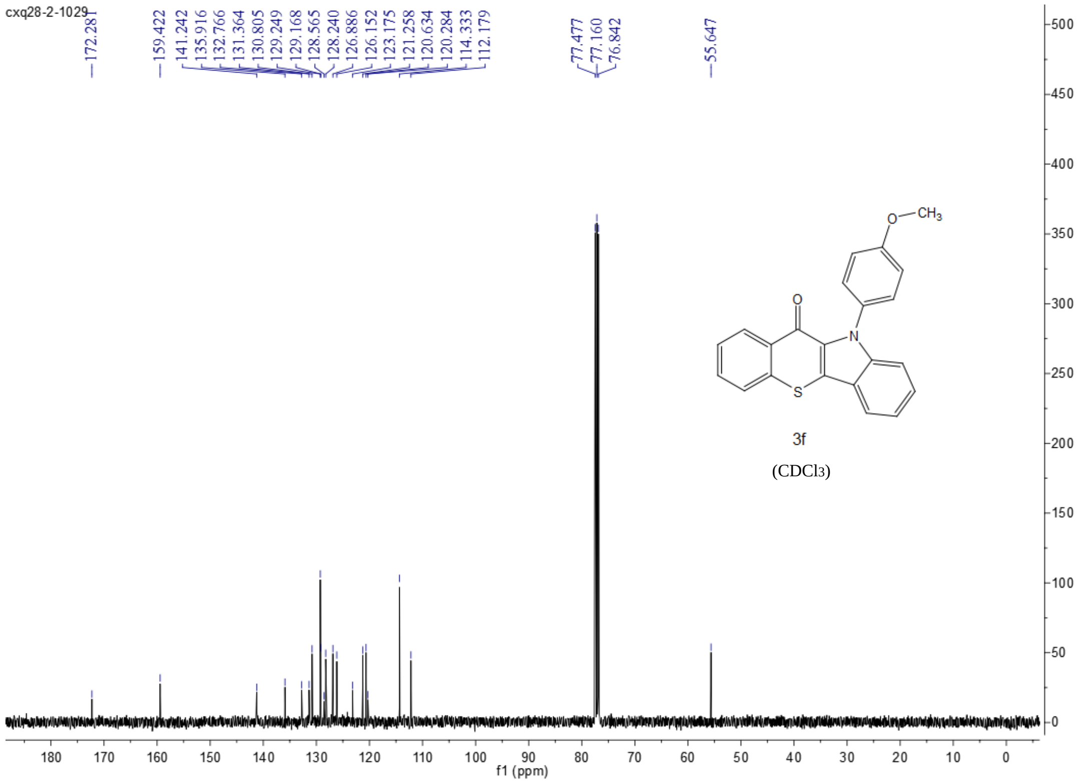
cxq28-2-1029
172.281
159.422
141.242
135.916
132.766
131.364
130.805
129.249
129.168
128.565
128.240
126.886
126.152
123.175
121.258
120.634
120.284
114.333
112.179
77.477
77.160
76.842
55.647
f1 (ppm)
3f
(CDCl3)

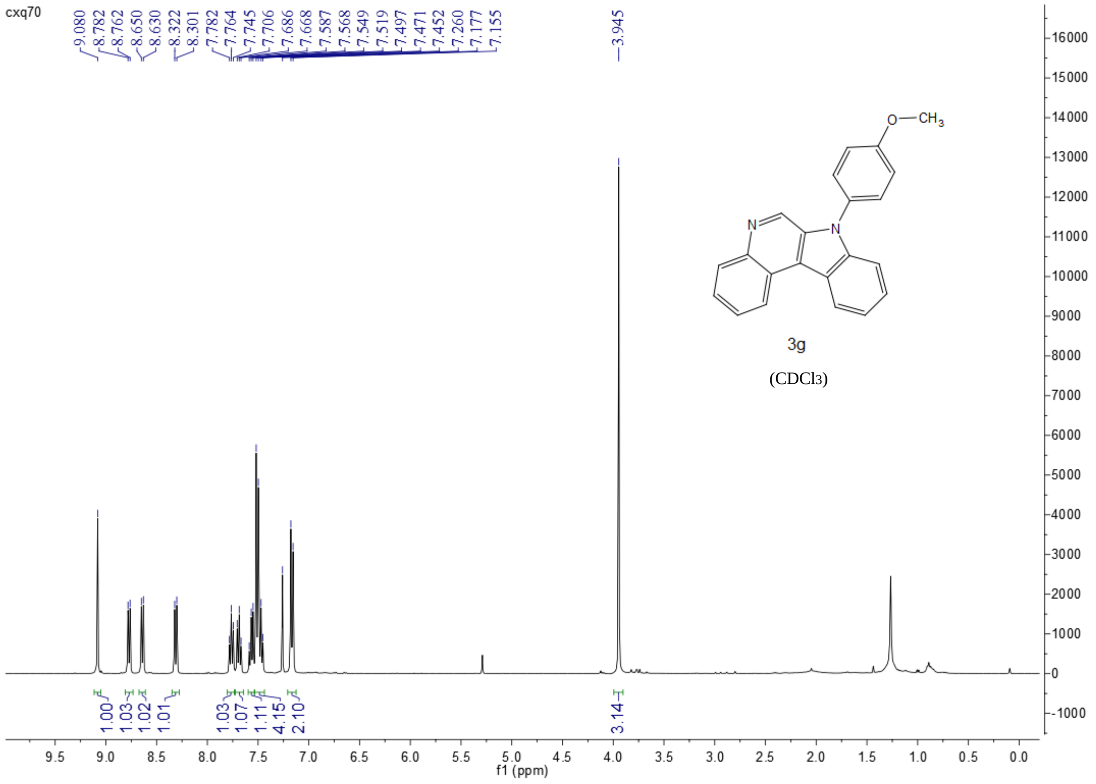
cxq70
9.080
8.782
8.762
8.650
8.630
8.322
8.301
7.782
7.764
7.745
7.706
7.686
7.668
7.587
7.568
7.549
7.519
7.497
7.471
7.452
7.260
7.177
7.155
3.945
1.00
1.03
1.02
1.01
1.03
1.07
1.11
4.15
2.10
3.14
f1 (ppm)
3g
(CDCl3)

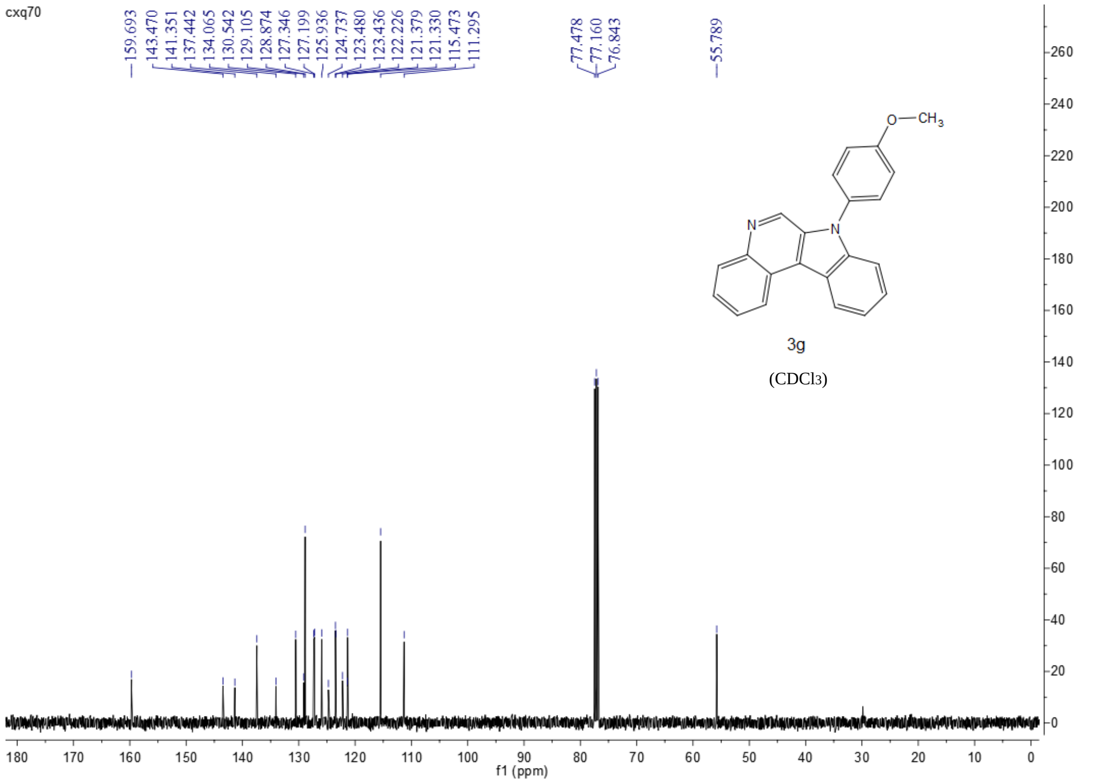
cxq70
159.693
143.470
141.351
137.442
134.065
130.542
129.105
128.874
127.346
127.199
125.936
124.737
123.480
123.436
122.226
121.379
121.330
115.473
111.295
77.478
77.160
76.843
55.789
3g
(CDCl3)
f1 (ppm)

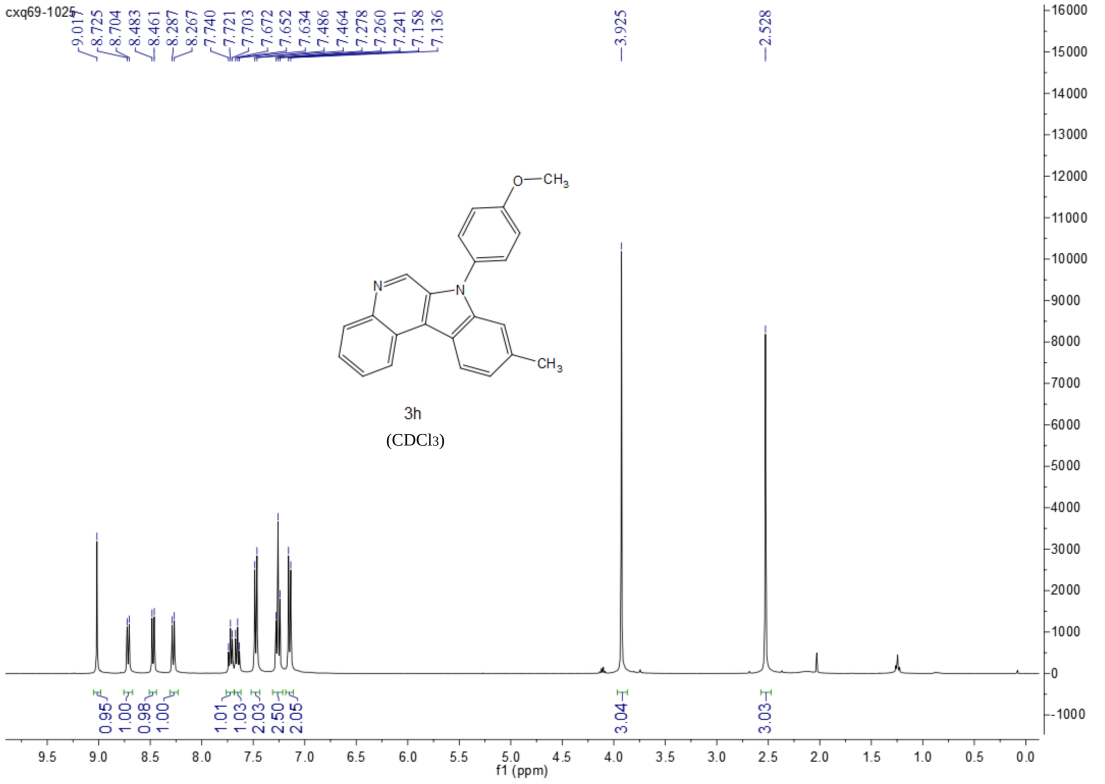
cxq69-1025
9.017
8.725
8.704
8.483
8.461
8.287
8.267
7.740
7.721
7.703
7.672
7.652
7.634
7.486
7.464
7.278
7.260
7.241
7.158
7.136
3.925
2.528
3h
(CDCl3)
0.95
1.00
0.98
1.00
1.01
1.03
2.03
2.50
2.05
3.04
3.03
f1 (ppm)

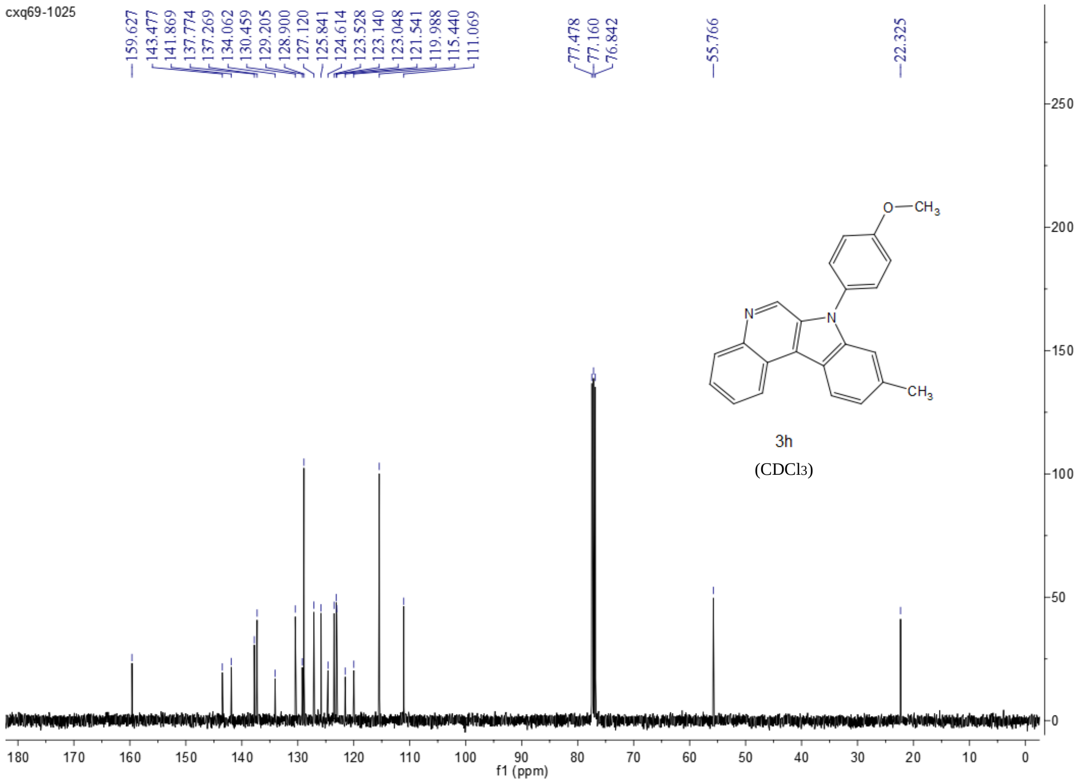
cxq69-1025
159.627
143.477
141.869
137.774
137.269
134.062
130.459
129.205
128.900
127.120
125.841
124.614
123.528
123.140
123.048
121.541
119.988
115.440
111.069
77.478
77.160
76.842
55.766
22.325
N
N
O
CH3
CH3
3h
(CDCl3)
f1 (ppm)

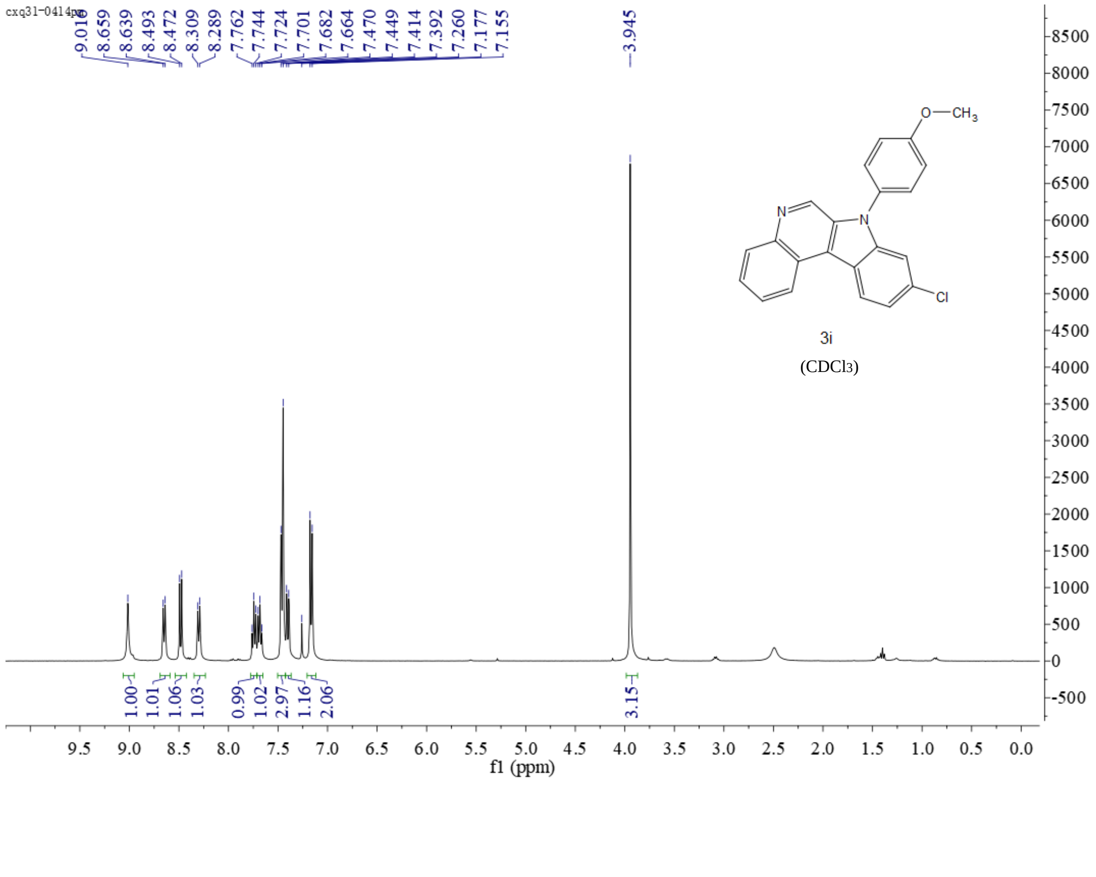
cxq31-0414pg
9.016
8.659
8.639
8.493
8.472
8.309
8.289
7.762
7.744
7.724
7.701
7.682
7.664
7.470
7.449
7.414
7.392
7.260
7.177
7.155
3.945
N
N
O
CH3
Cl
3i
(CDCl3)
1.00
1.01
1.06
1.03
0.99
1.02
2.97
1.16
2.06
3.15
f1 (ppm)

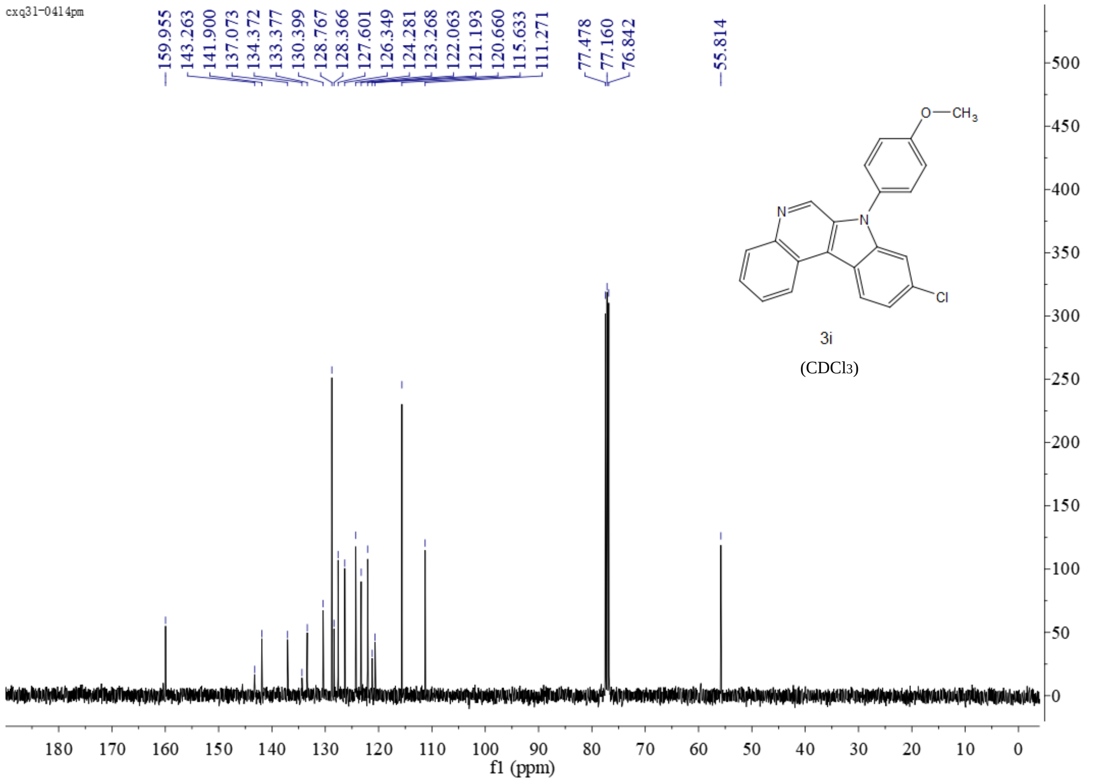
cxq31-0414pm
159.955
143.263
141.900
137.073
134.372
133.377
130.399
128.767
128.366
127.601
126.349
124.281
123.268
122.063
121.193
120.660
115.633
111.271
77.478
77.160
76.842
55.814
O—CH3
N
N
Cl
3i
(CDCl3)
f1 (ppm)

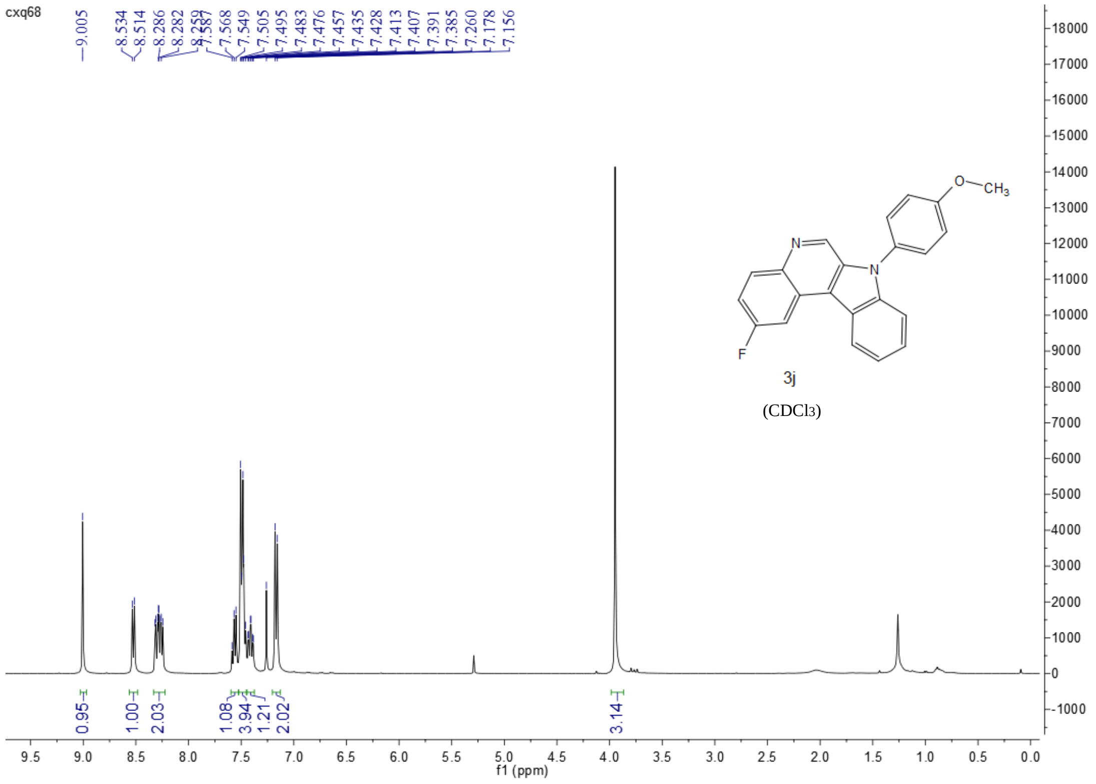
cxq68
9.005
8.534
8.514
8.286
8.282
8.259
7.587
7.568
7.549
7.505
7.495
7.483
7.476
7.457
7.435
7.428
7.413
7.407
7.391
7.385
7.260
7.178
7.156
O—CH3
N
N
F
3j
(CDCl3)
0.95
1.00
2.03
1.08
3.94
1.21
2.02
3.14
f1 (ppm)

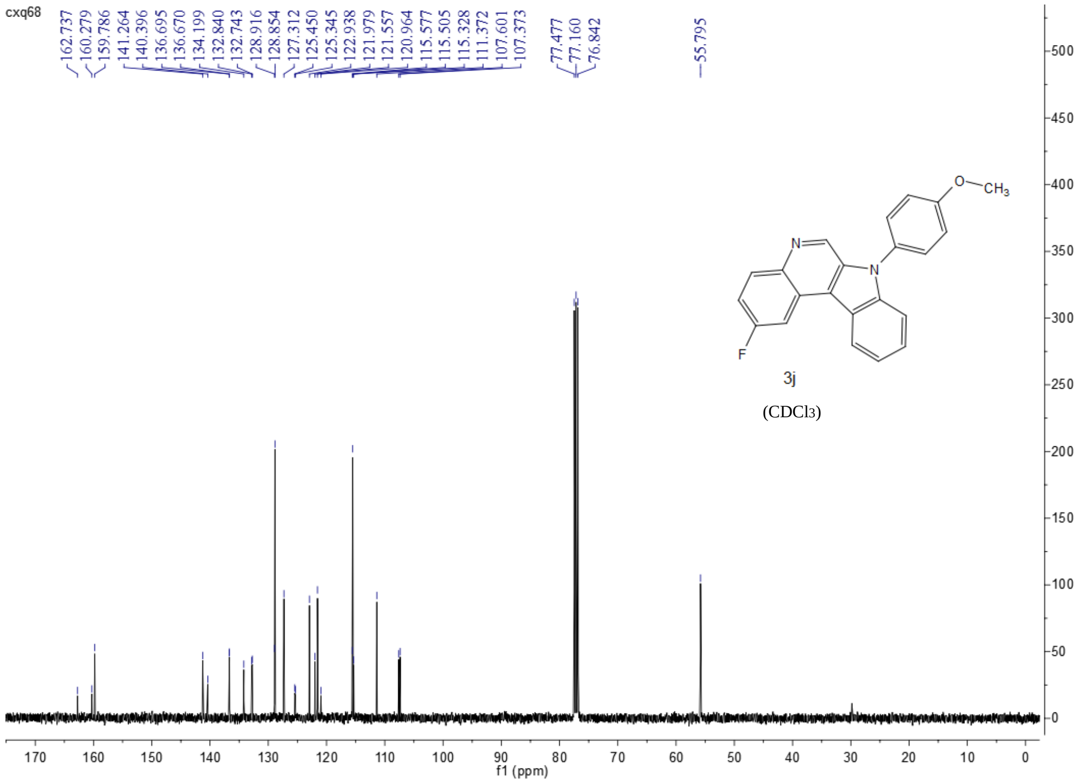
cxq68
162.737
160.279
159.786
141.264
140.396
136.695
136.670
134.199
132.840
132.743
128.916
128.854
127.312
125.450
125.345
122.938
121.979
121.557
120.964
115.577
115.505
115.328
111.372
107.601
107.373
77.477
77.160
76.842
55.795
3j
(CDCl3)
f1 (ppm)

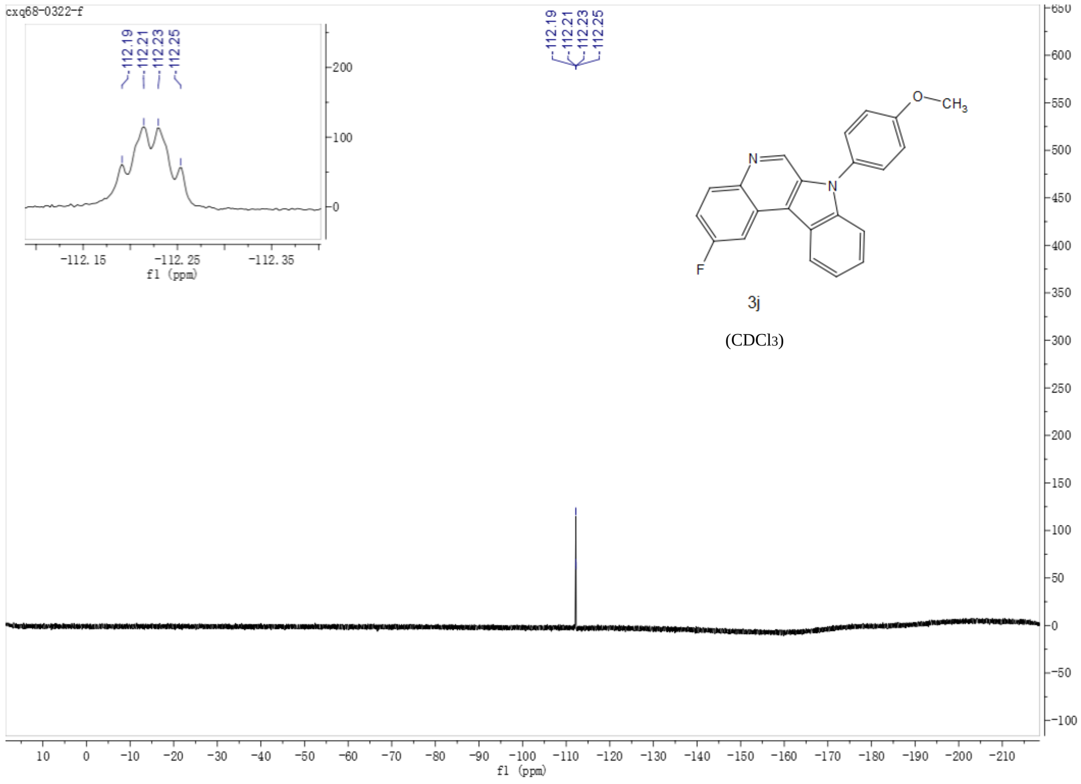
cxq68-0322-f
-112.19
-112.21
-112.23
-112.25
3j
(CDCl3)
f1 (ppm)

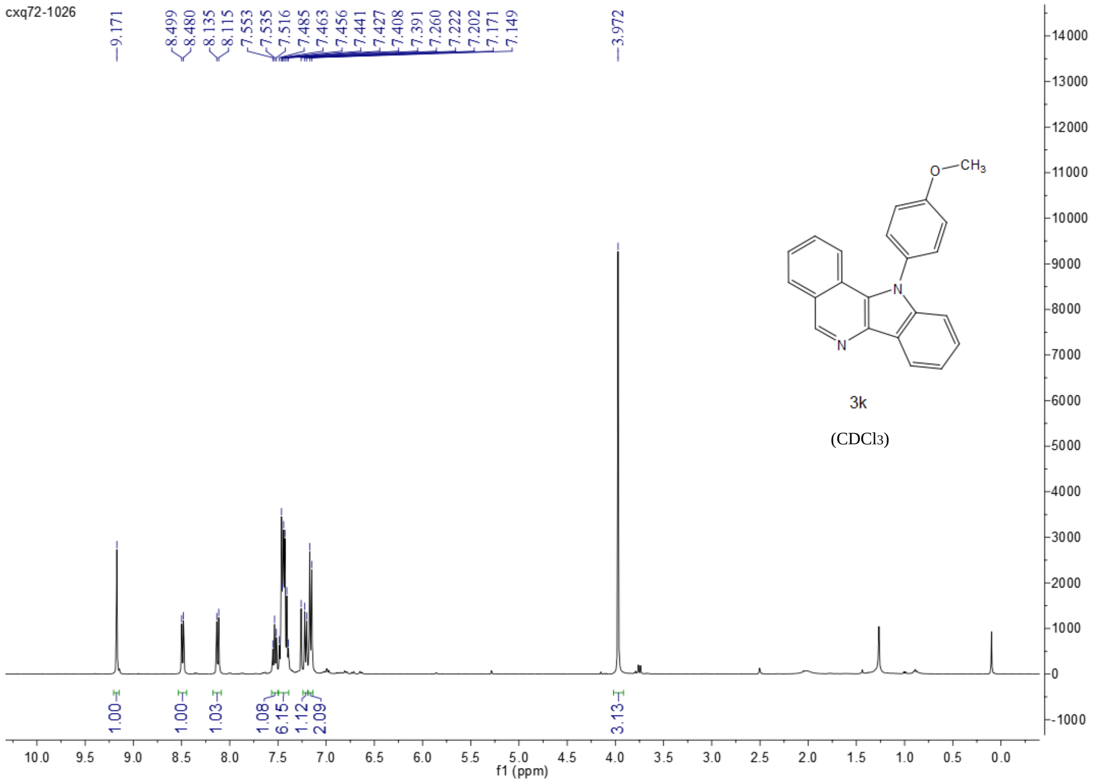
cxq72-1026
9.171
8.499
8.480
8.135
8.115
7.553
7.535
7.516
7.485
7.463
7.456
7.441
7.427
7.408
7.391
7.260
7.222
7.202
7.171
7.149
3.972
3k
(CDCl3)
1.00
1.00
1.03
1.08
6.15
1.12
2.09
3.13
f1 (ppm)

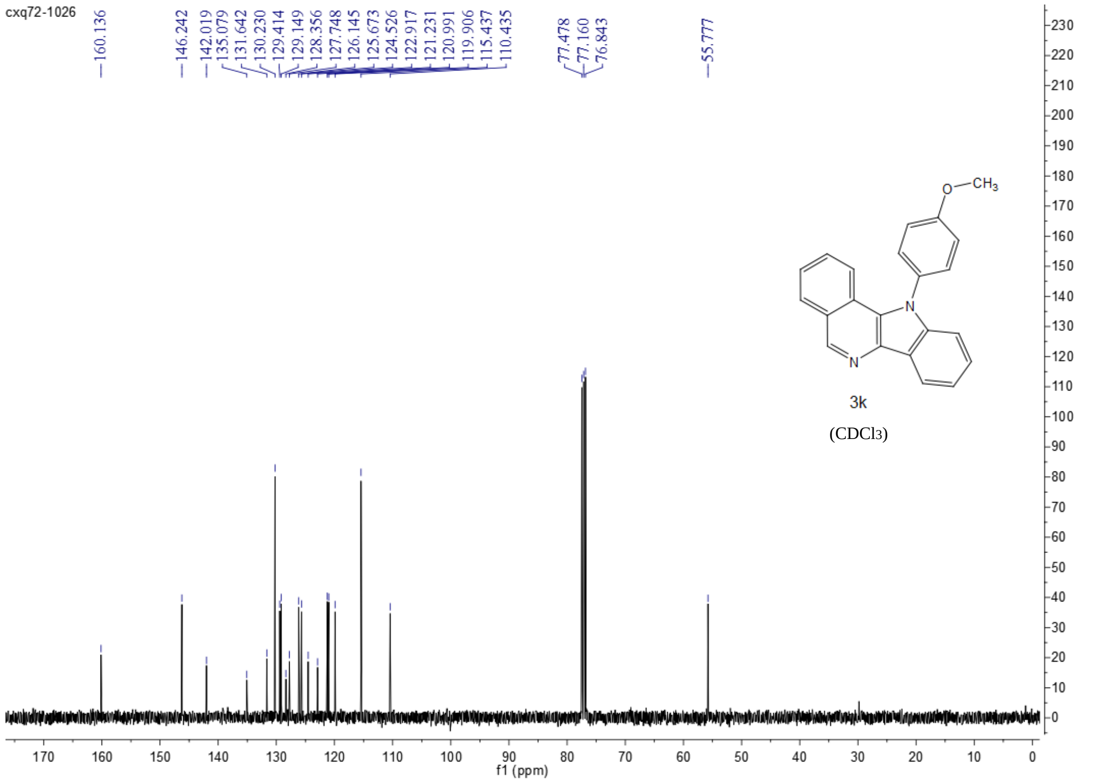
cxq72-1026
160.136
146.242
142.019
135.079
131.642
130.230
129.414
129.149
128.356
127.748
126.145
125.673
124.526
122.917
121.231
120.991
119.906
115.437
110.435
77.478
77.160
76.843
55.777
3k
(CDCl3)
f1 (ppm)

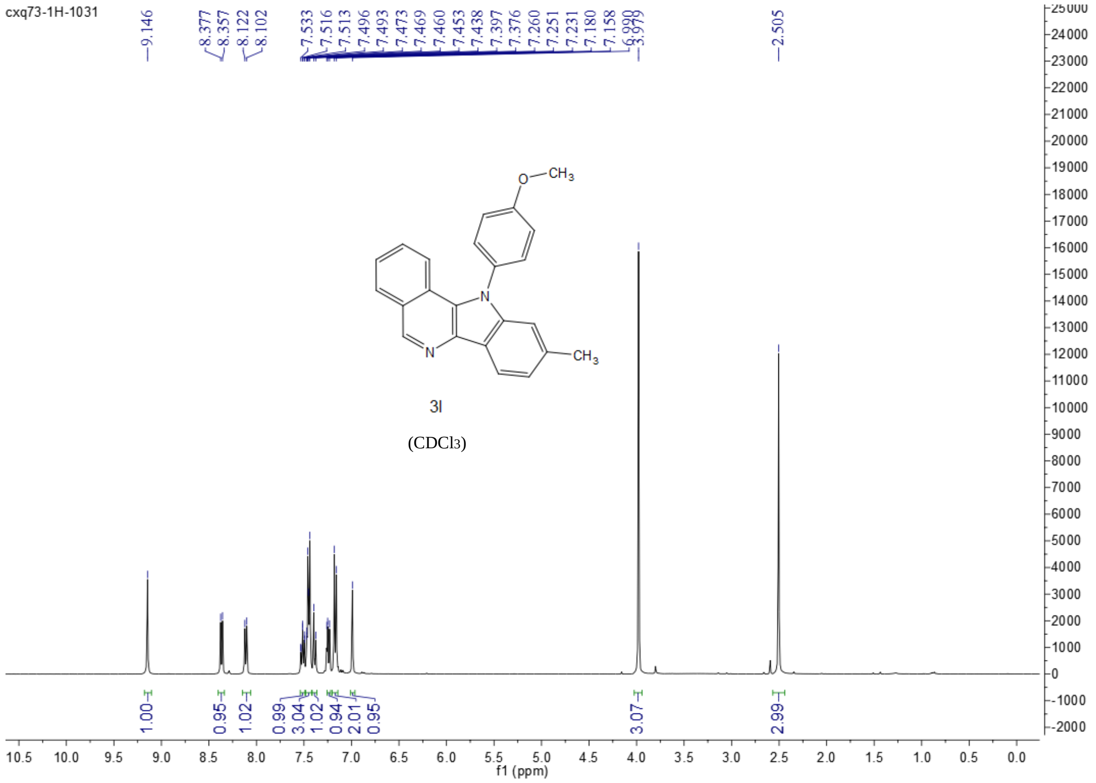
cxq73-1H-1031
9.146
8.377
8.357
8.122
8.102
7.533
7.516
7.513
7.496
7.493
7.473
7.469
7.460
7.453
7.438
7.397
7.376
7.260
7.251
7.231
7.180
7.158
6.990
3.979
2.505
O
CH3
N
N
CH3
3l
(CDCl3)
1.00
0.95
1.02
0.99
3.04
1.02
0.94
2.01
0.95
3.07
2.99
f1 (ppm)

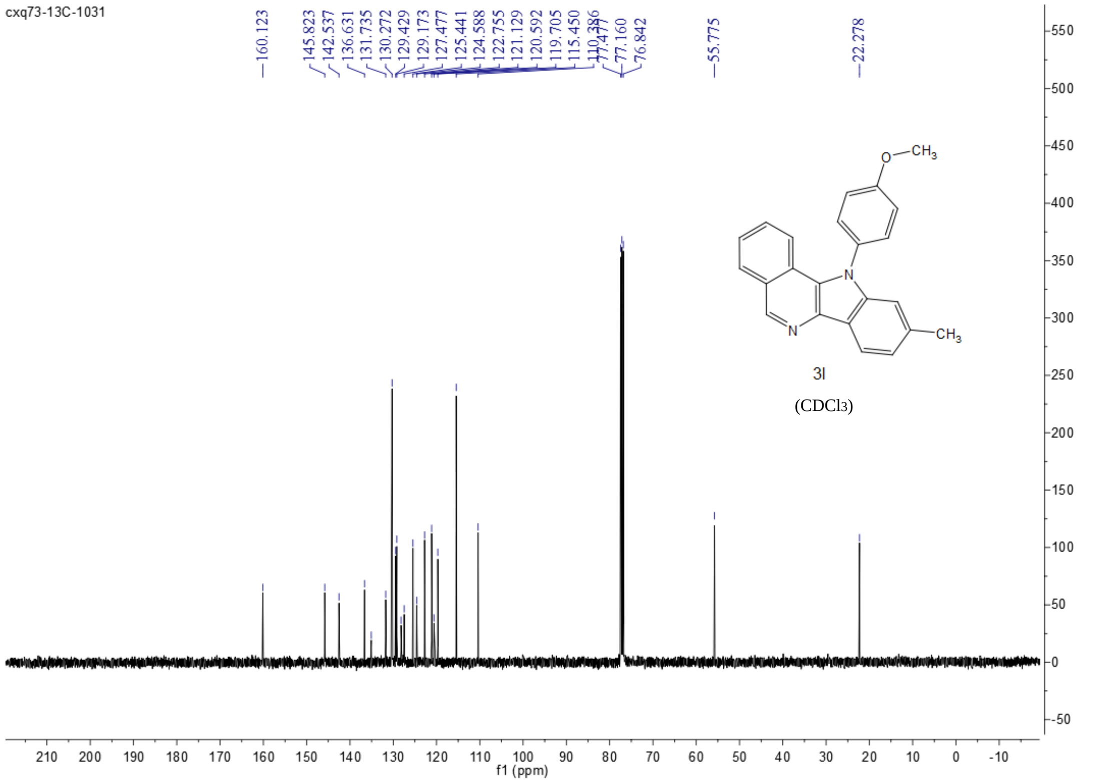
cxq73-13C-1031
160.123
145.823
142.537
136.631
131.735
130.272
129.429
129.173
127.477
125.441
124.588
122.755
121.129
120.592
119.705
115.450
110.386
77.477
77.160
76.842
55.775
22.278
O
CH3
N
N
CH3
3l
(CDCl3)
f1 (ppm)

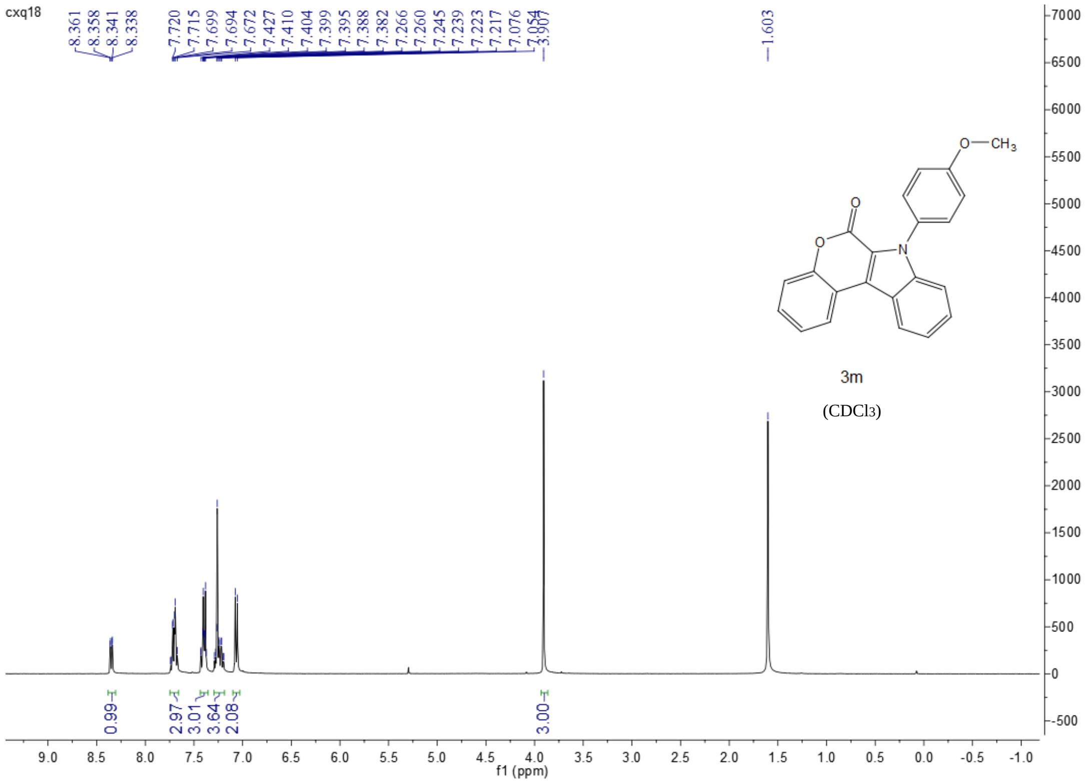
cxq18
8.361
8.358
8.341
8.338
7.720
7.715
7.699
7.694
7.672
7.427
7.410
7.404
7.399
7.395
7.388
7.382
7.266
7.260
7.245
7.239
7.223
7.217
7.076
7.054
3.907
1.603
0.99
2.97
3.01
3.64
2.08
3.00
f1 (ppm)
3m
(CDCl3)

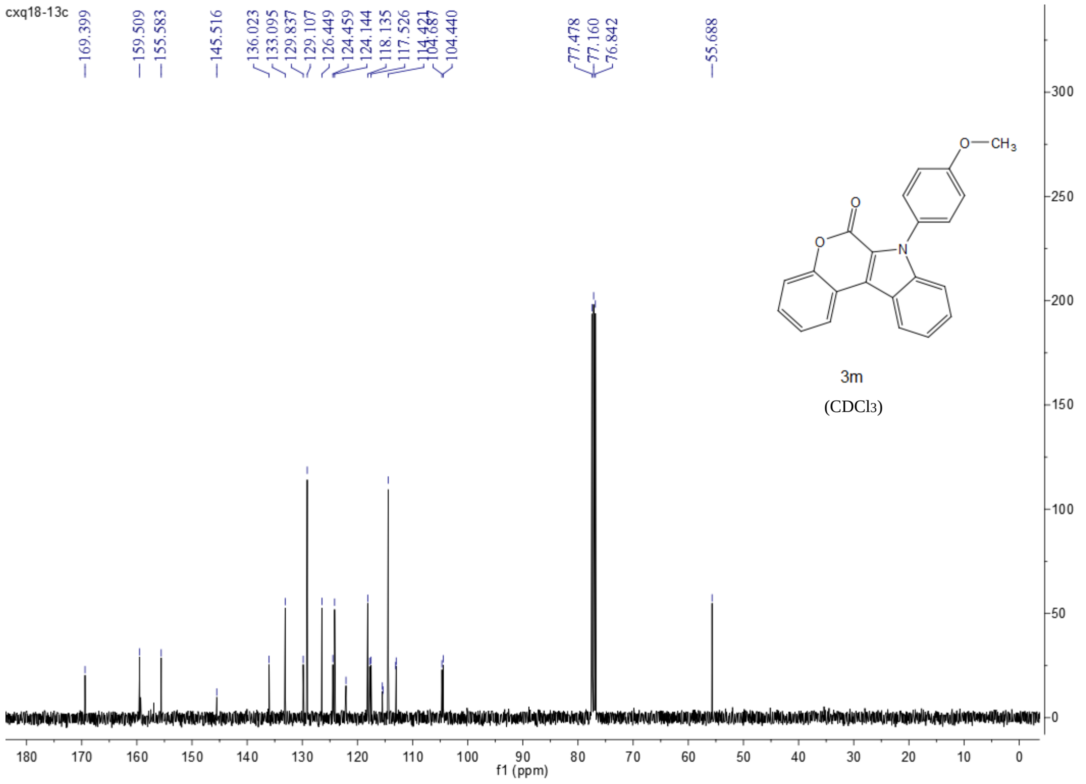
cxq18-13c
169.399
159.509
155.583
145.516
136.023
133.095
129.837
129.107
126.449
124.459
124.144
118.135
117.526
114.421
104.687
104.440
77.478
77.160
76.842
55.688
f1 (ppm)
3m
(CDCl3)

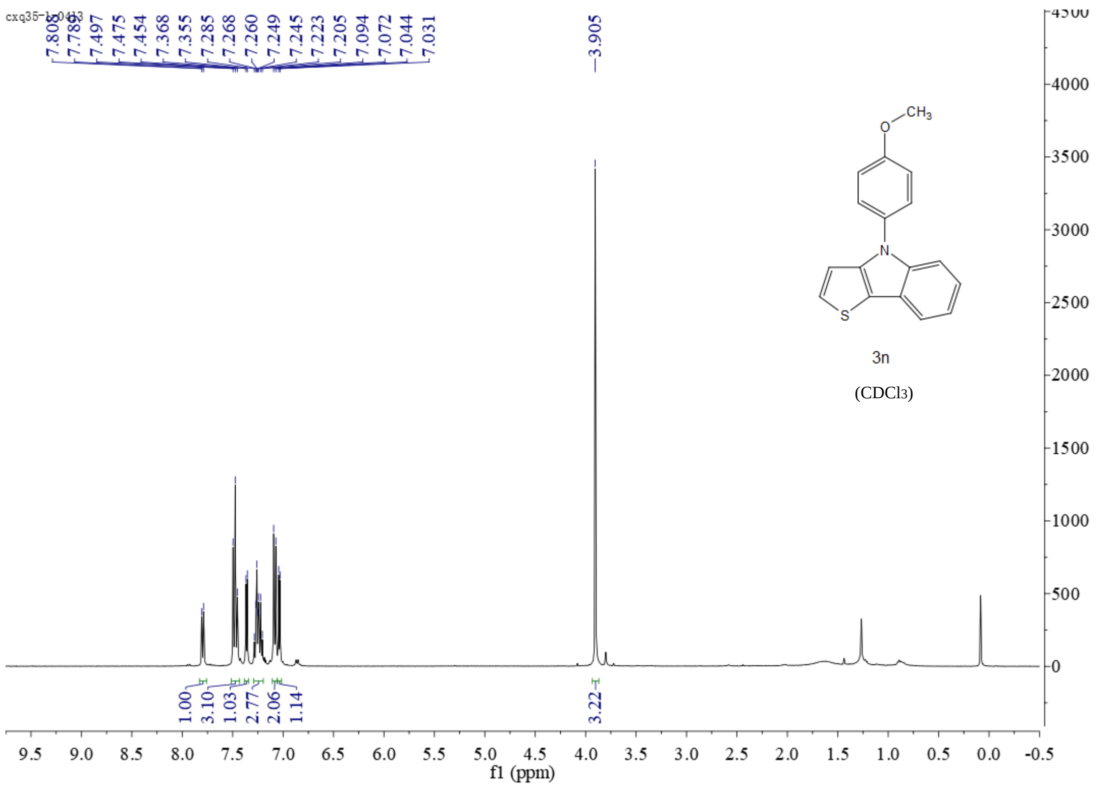
cxq35-1-0413
7.805
7.789
7.497
7.475
7.454
7.368
7.355
7.285
7.268
7.260
7.249
7.245
7.223
7.205
7.094
7.072
7.044
7.031
3.905
1.00
3.10
1.03
2.77
2.06
1.14
3.22
f1 (ppm)
CH3
O
N
S
3n
(CDCl3)

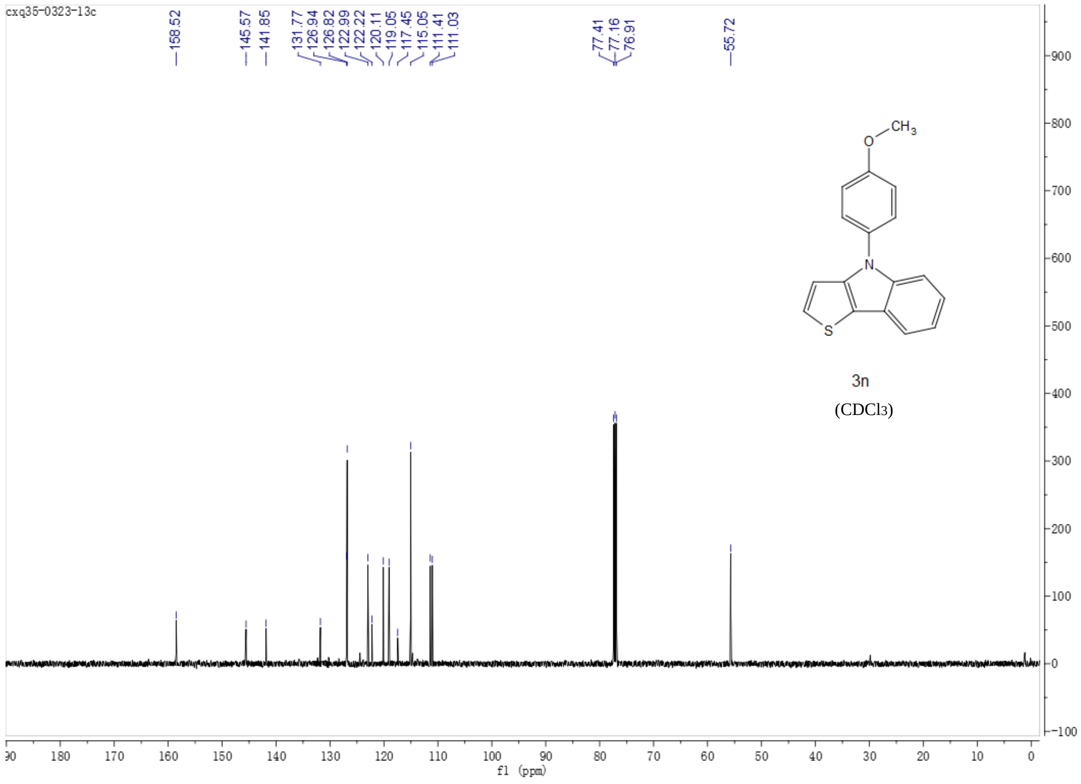
cxq35-0323-13c
158.52
145.57
141.85
131.77
126.94
126.82
122.99
122.22
120.11
119.05
117.45
115.05
111.41
111.03
77.41
77.16
76.91
55.72
f1 (ppm)
CH3
O
N
S
3n
(CDCl3)

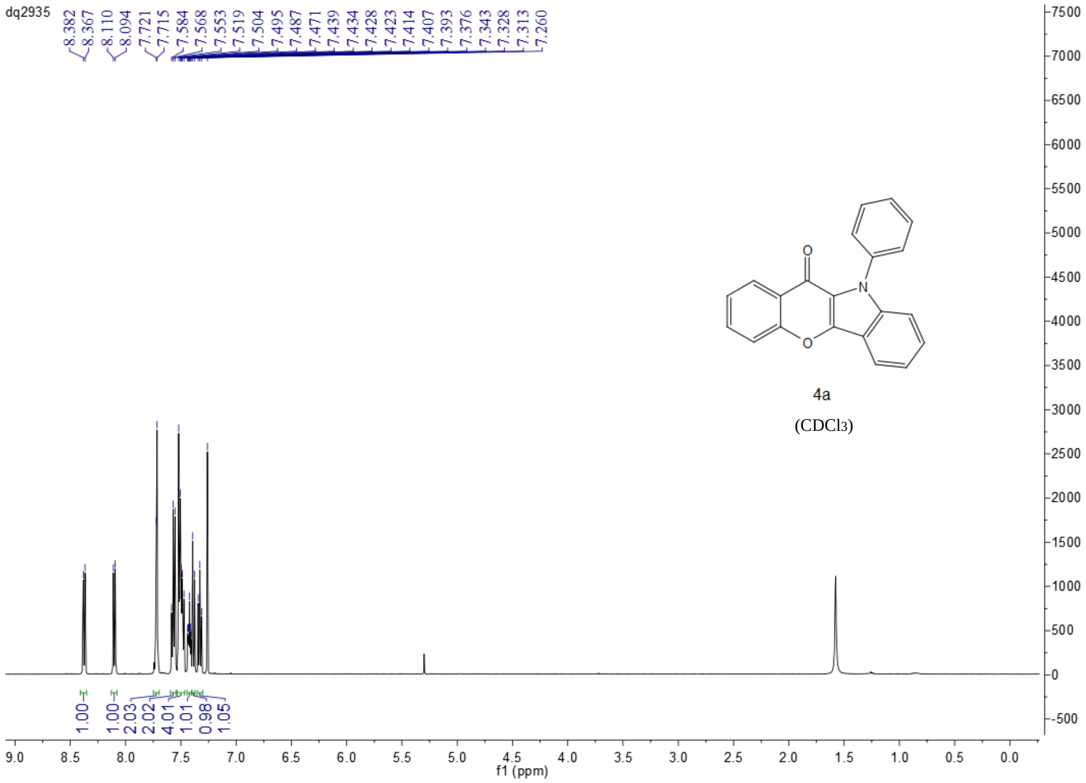
dq2935
8.382
8.367
8.110
8.094
7.721
7.715
7.584
7.568
7.553
7.519
7.504
7.495
7.487
7.471
7.439
7.434
7.428
7.423
7.414
7.407
7.393
7.376
7.343
7.328
7.313
7.260
O
N
O
4a
(CDCl3)
1.00
1.00
2.03
2.02
4.01
1.01
0.98
1.05
f1 (ppm)

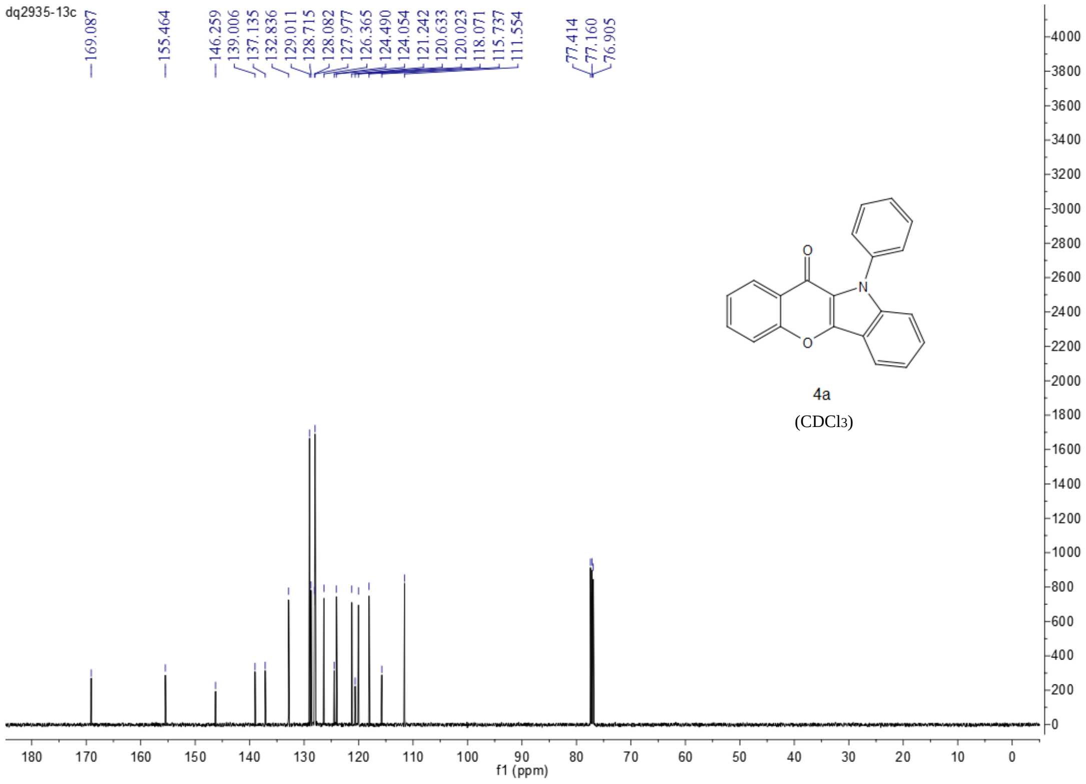
dq2935-13c
169.087
155.464
146.259
139.006
137.135
132.836
129.011
128.715
128.082
127.977
126.365
124.490
124.054
121.242
120.633
120.023
118.071
115.737
111.554
77.414
77.160
76.905
O
N
O
4a
(CDCl3)
f1 (ppm)

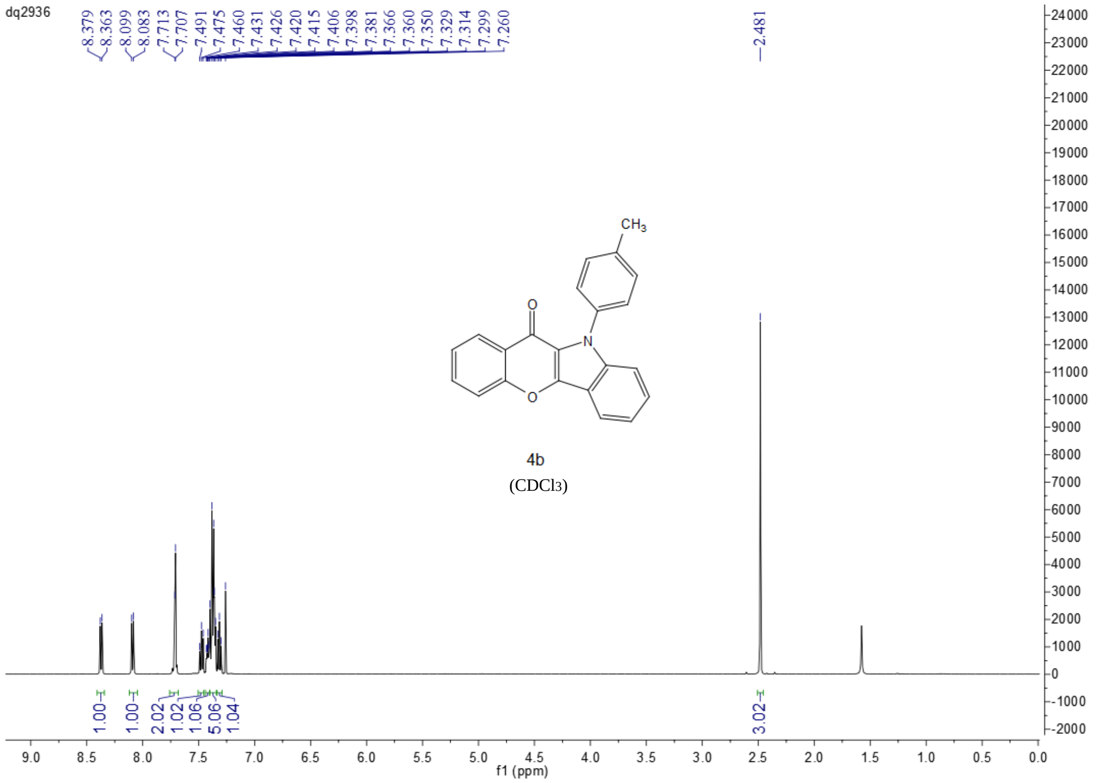
dq2936
8.379
8.363
8.099
8.083
7.713
7.707
7.491
7.475
7.460
7.431
7.426
7.420
7.415
7.406
7.398
7.381
7.366
7.360
7.350
7.329
7.314
7.299
7.260
2.481
CH3
O
N
O
4b
(CDCl3)
1.00
1.00
2.02
1.02
1.06
5.06
1.04
3.02
f1 (ppm)

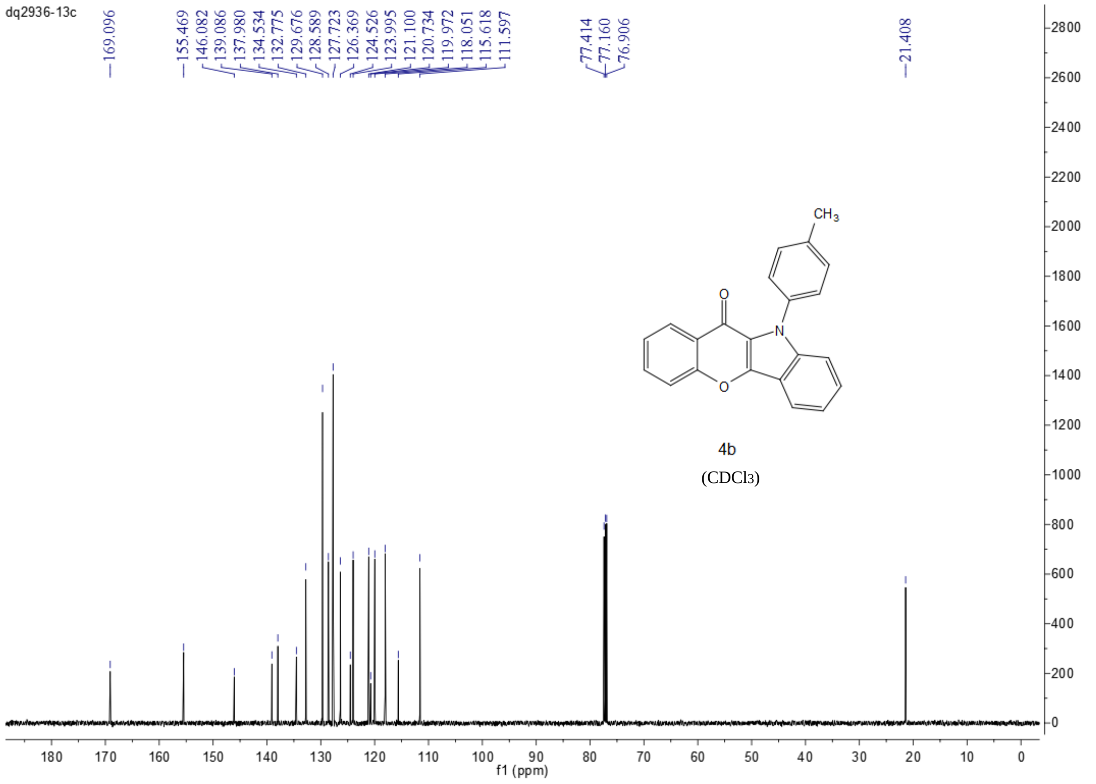
dq2936-13c
169.096
155.469
146.082
139.086
137.980
134.534
132.775
129.676
128.589
127.723
126.369
124.526
123.995
121.100
120.734
119.972
118.051
115.618
111.597
77.414
77.160
76.906
21.408
CH3
O
N
O
4b
(CDCl3)
f1 (ppm)

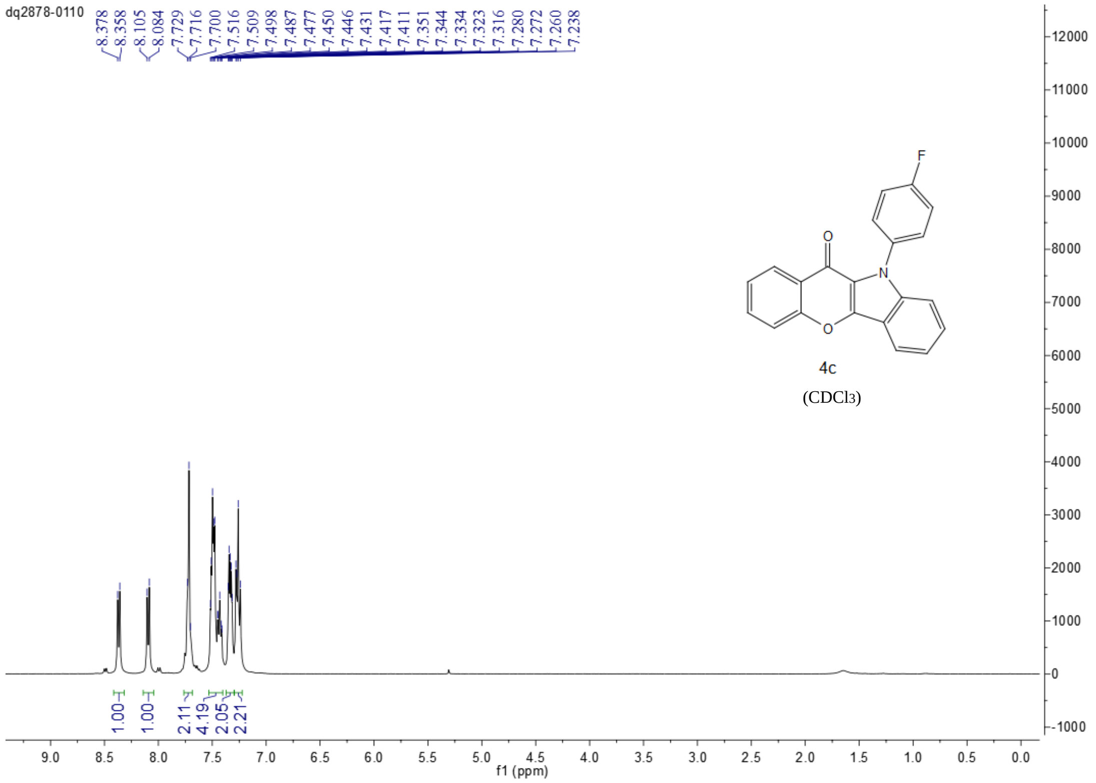
dq2878-0110
4c
(CDCl3)

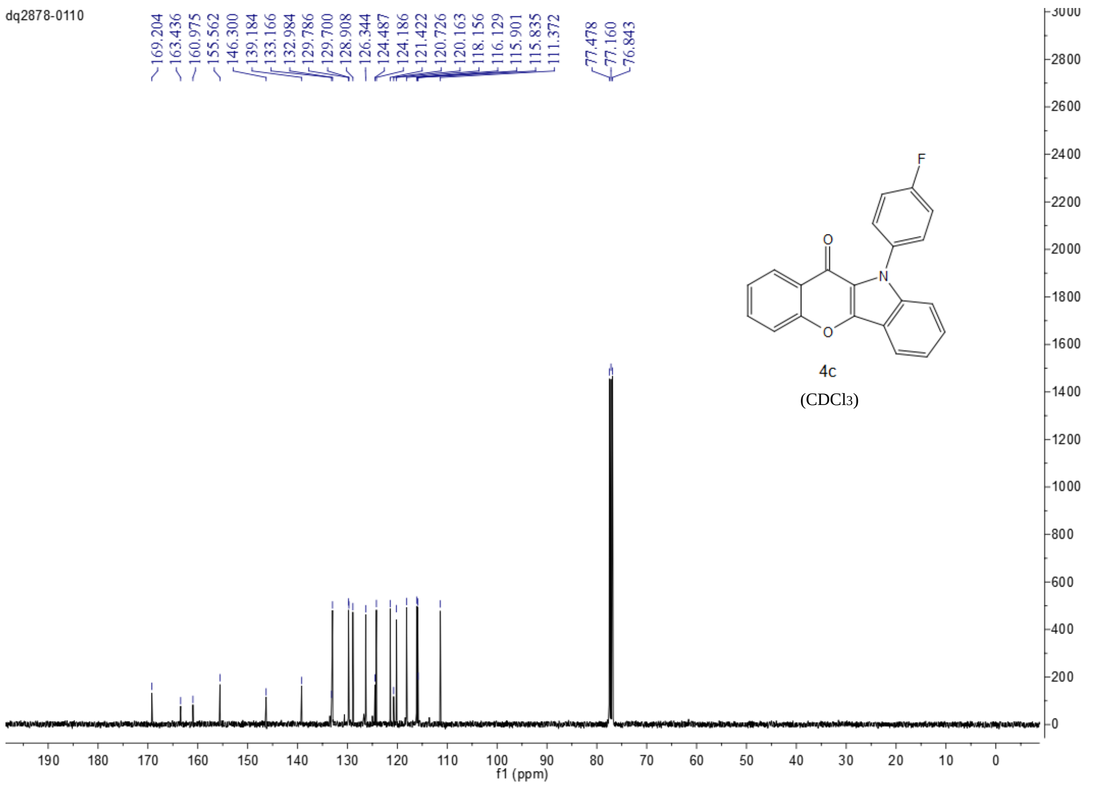
dq2878-0110
4c
(CDCl3)

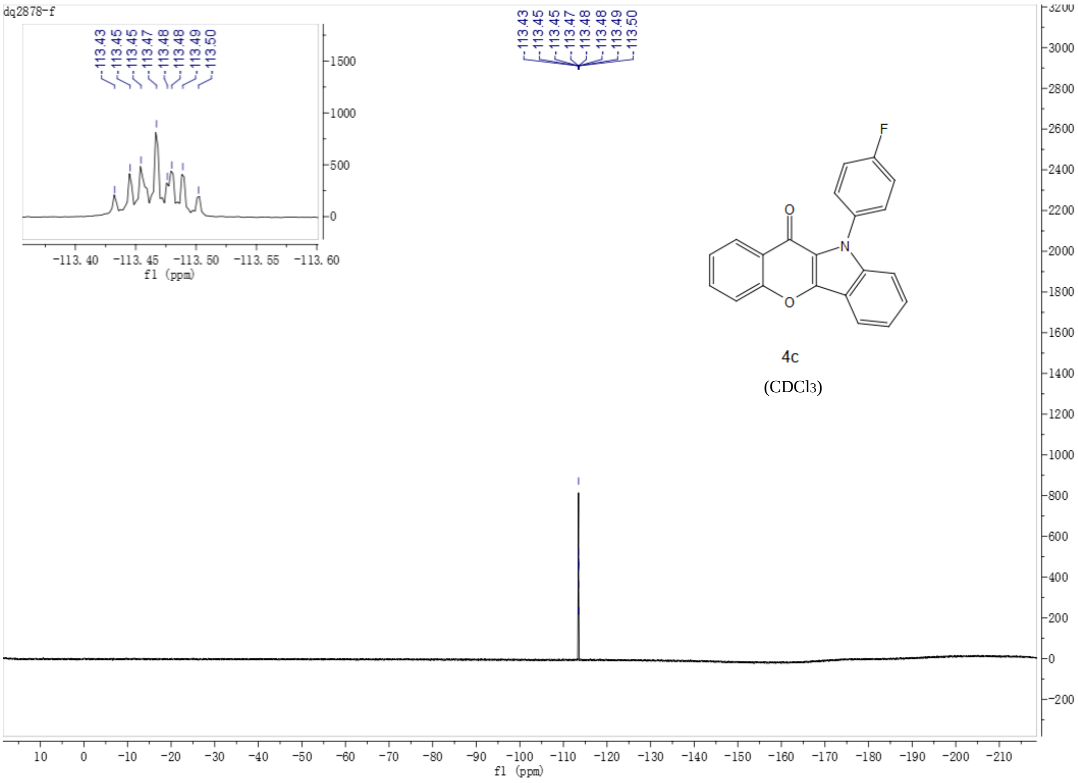
dq2878-f
-113.43
-113.45
-113.45
-113.47
-113.48
-113.48
-113.49
-113.50
f1 (ppm)
F
O
N
O
4c
(CDCl3)

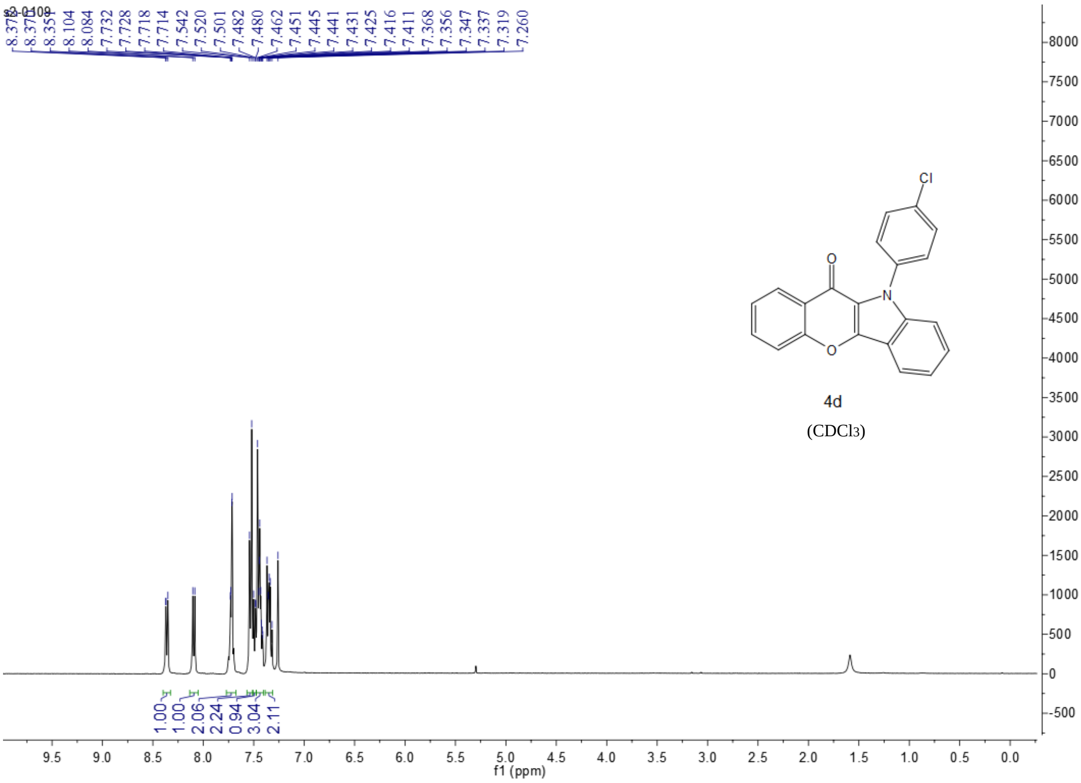
Cl
O
N
O
4d
(CDCl3)
f1 (ppm)

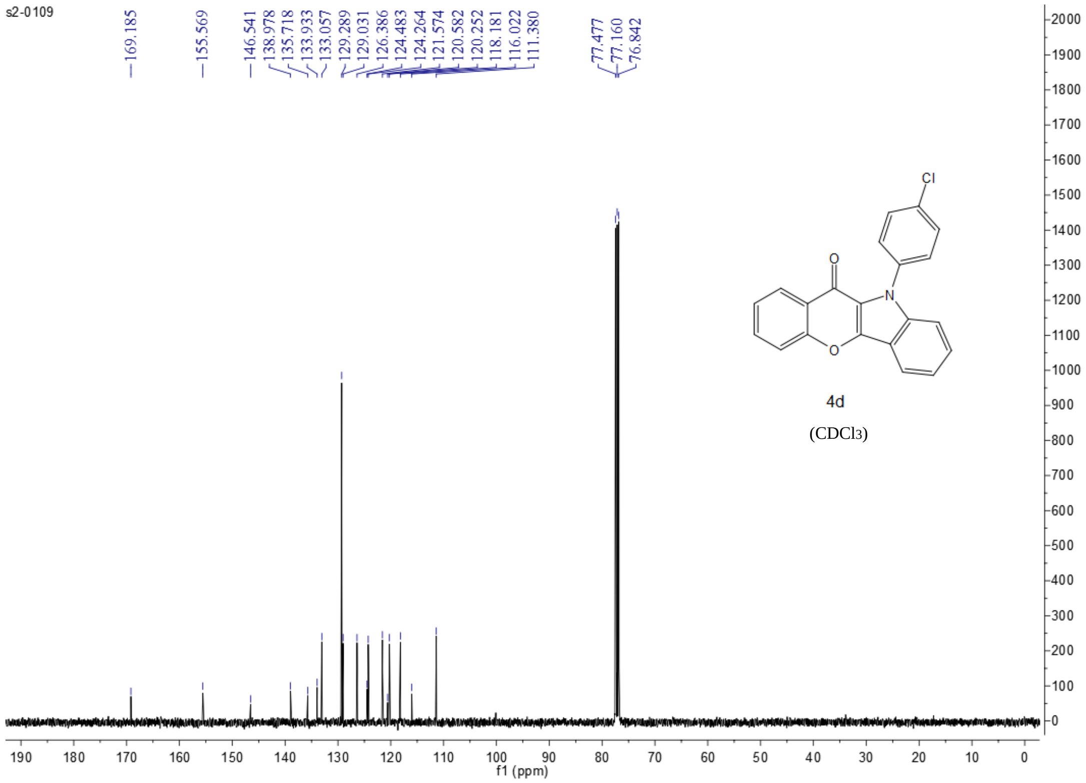
s2-0109
169.185
155.569
146.541
138.978
135.718
133.933
133.057
129.289
129.031
126.386
124.483
124.264
121.574
120.582
120.252
118.181
116.022
111.380
77.477
77.160
76.842
Cl
O
N
O
4d
(CDCl3)
f1 (ppm)

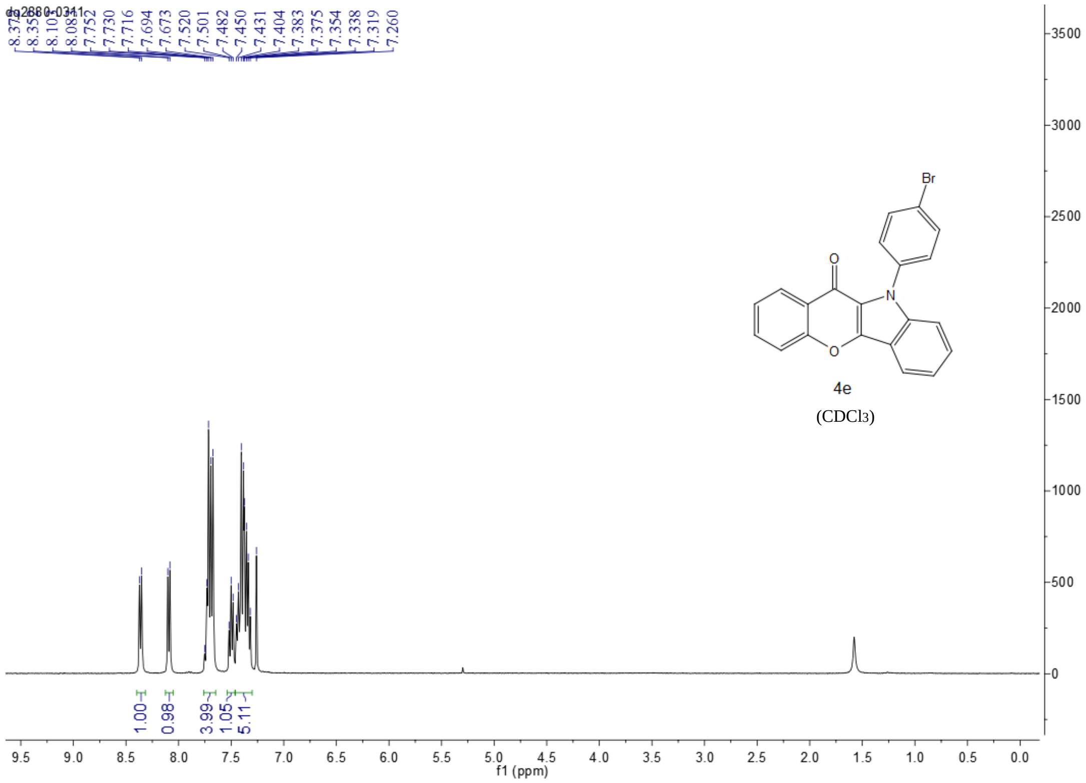
Br
O
N
O
4e
(CDCl3)
1.00
0.98
3.99
1.05
5.11
f1 (ppm)

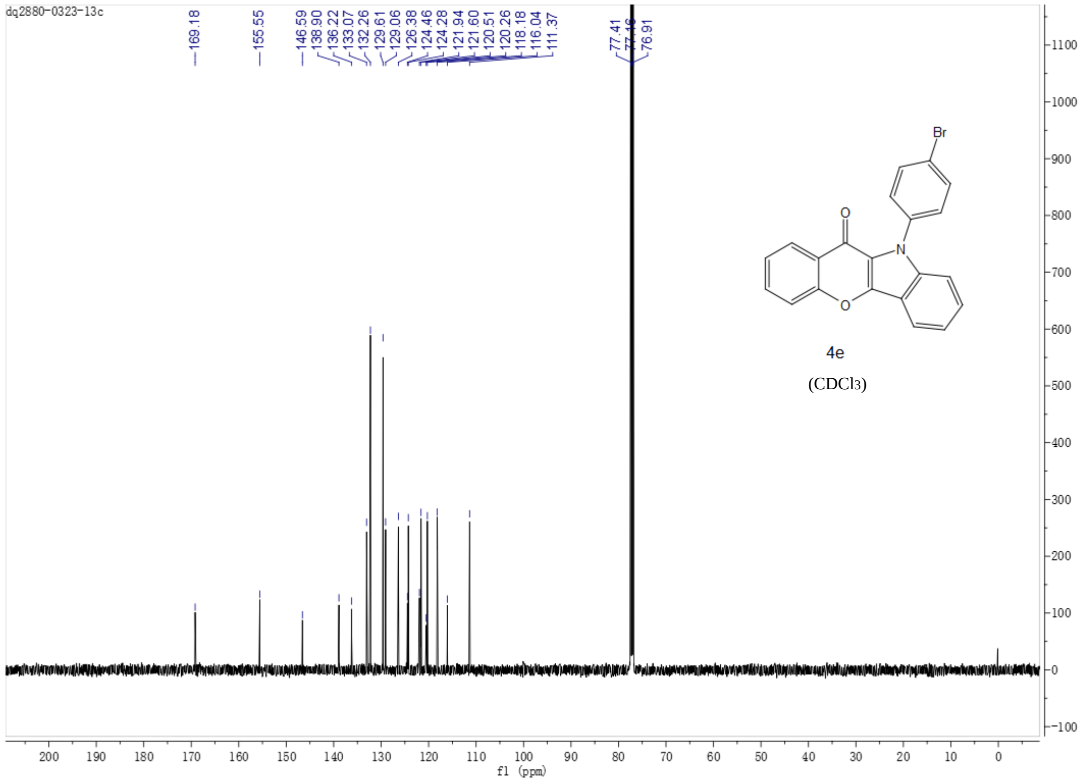

4e

(CDCl3)

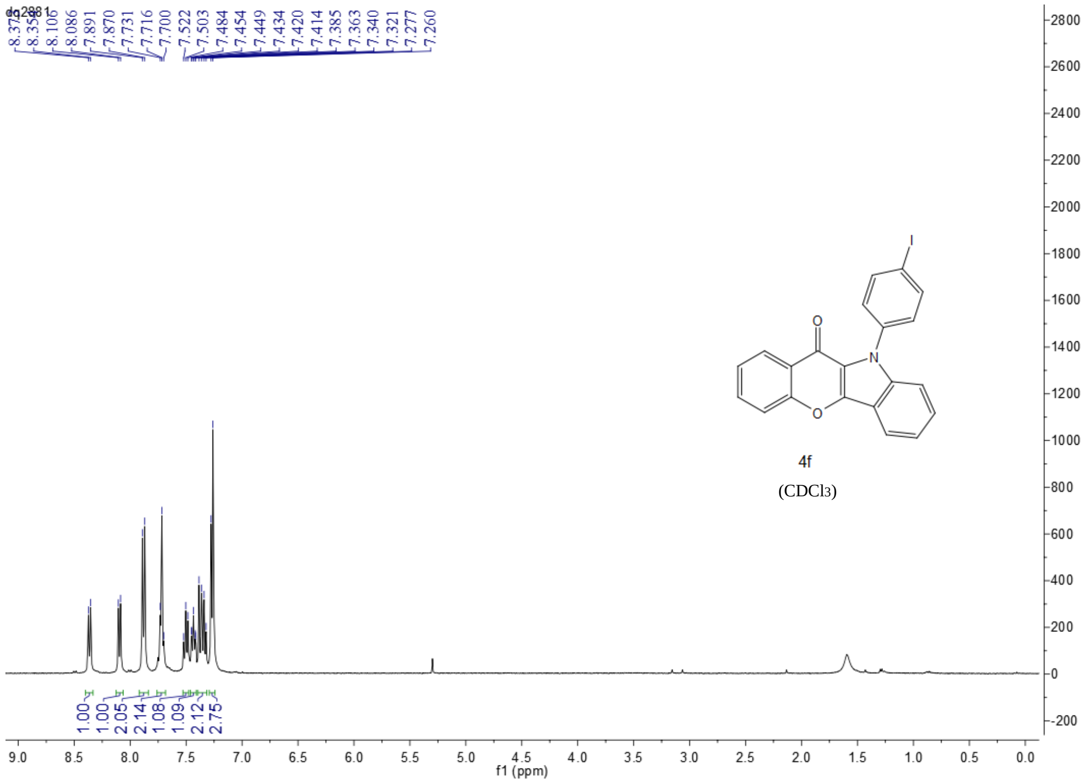

4f

(CDCl3)

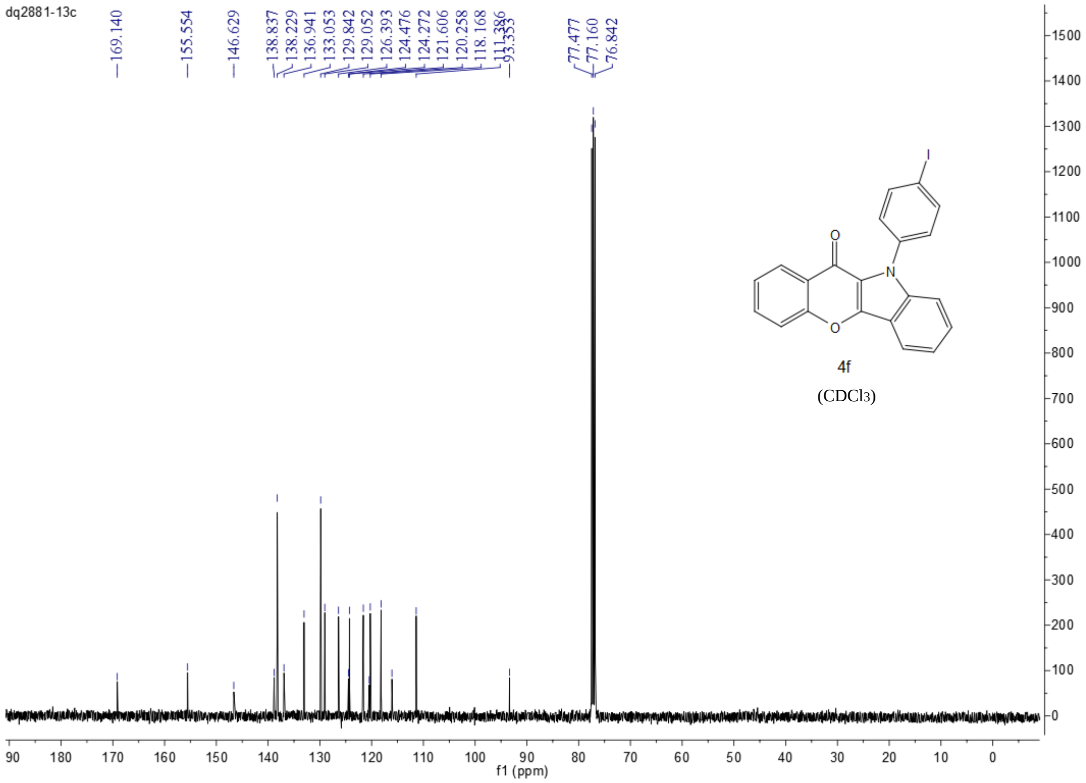
dq2881-13c
169.140
155.554
146.629
138.837
138.229
136.941
133.053
129.842
129.052
126.393
124.476
124.272
121.606
120.258
118.168
111.386
93.353
77.477
77.160
76.842
4f
(CDCl3)
f1 (ppm)

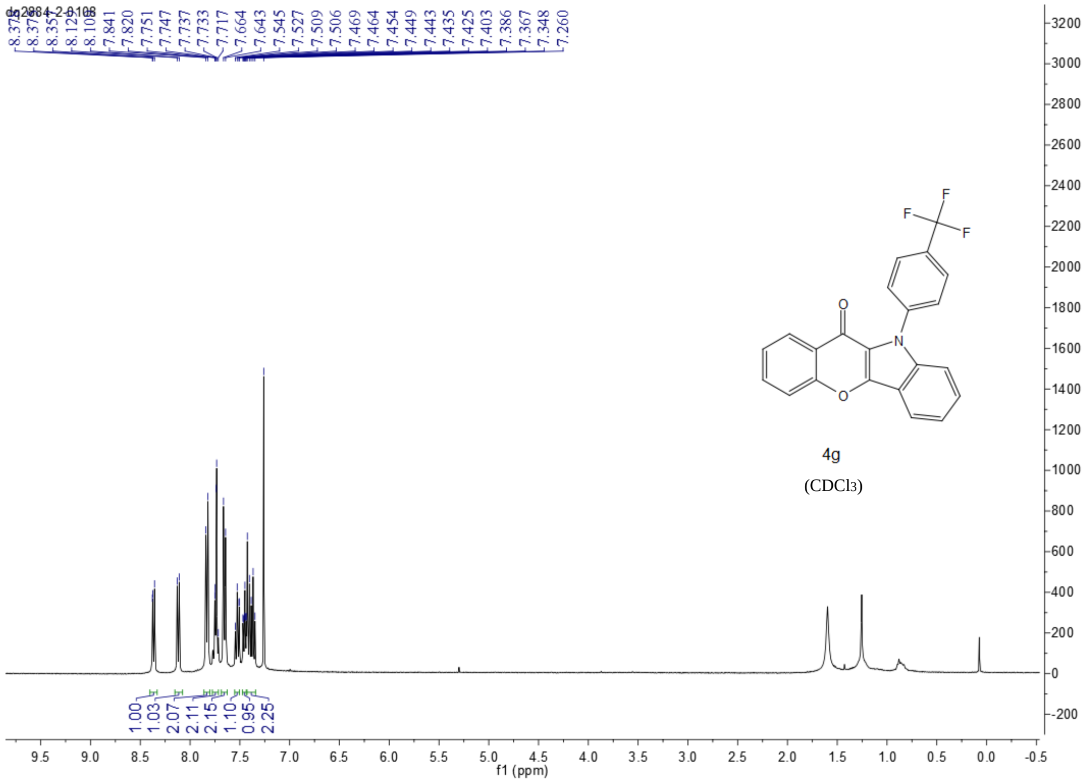
4g
(CDCl3)
f1 (ppm)

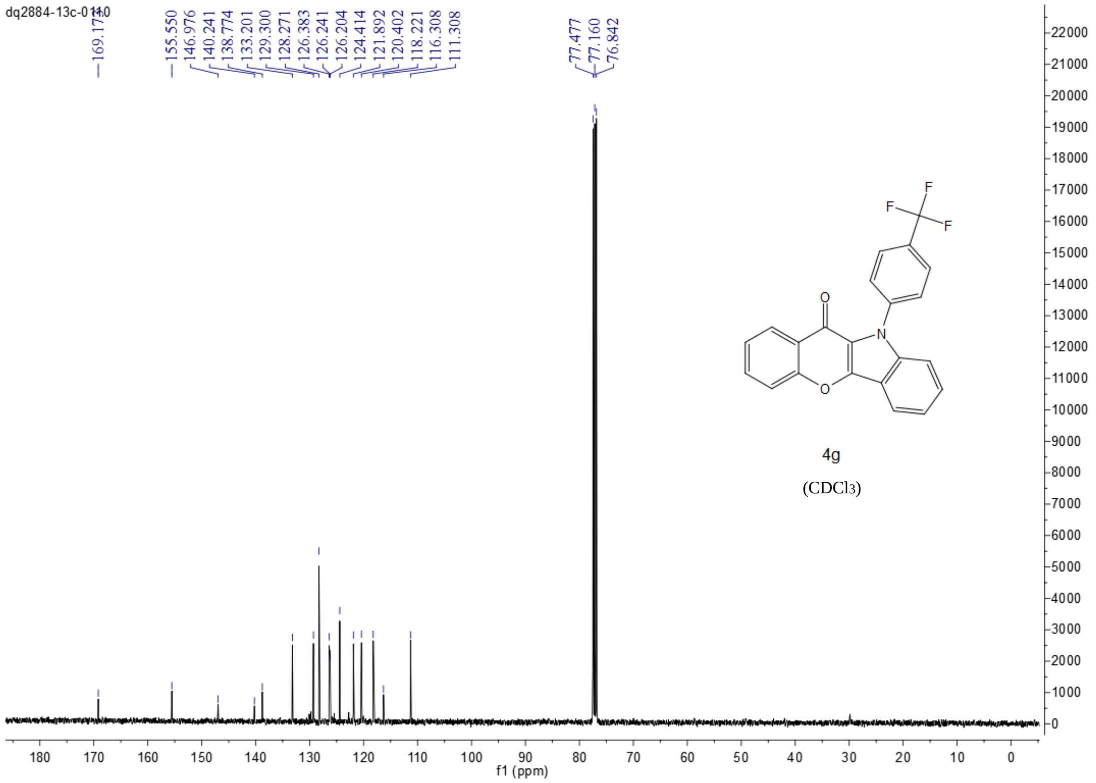
dq2884-13c-0110
169.174
155.550
146.976
140.241
138.774
133.201
129.300
128.271
126.383
126.241
126.204
124.414
121.892
120.402
118.221
116.308
111.308
77.477
77.160
76.842
4g
(CDCl3)
f1 (ppm)

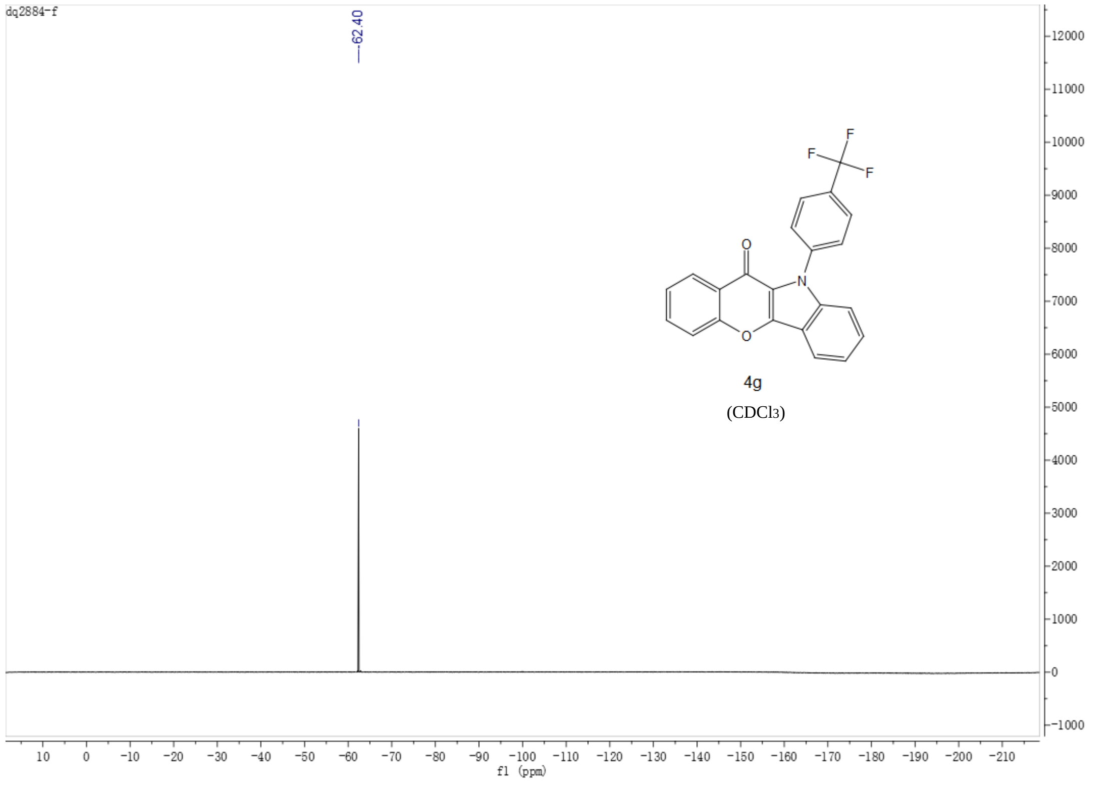
dq2884-f
-62.40
4g
(CDCl3)
f1 (ppm)

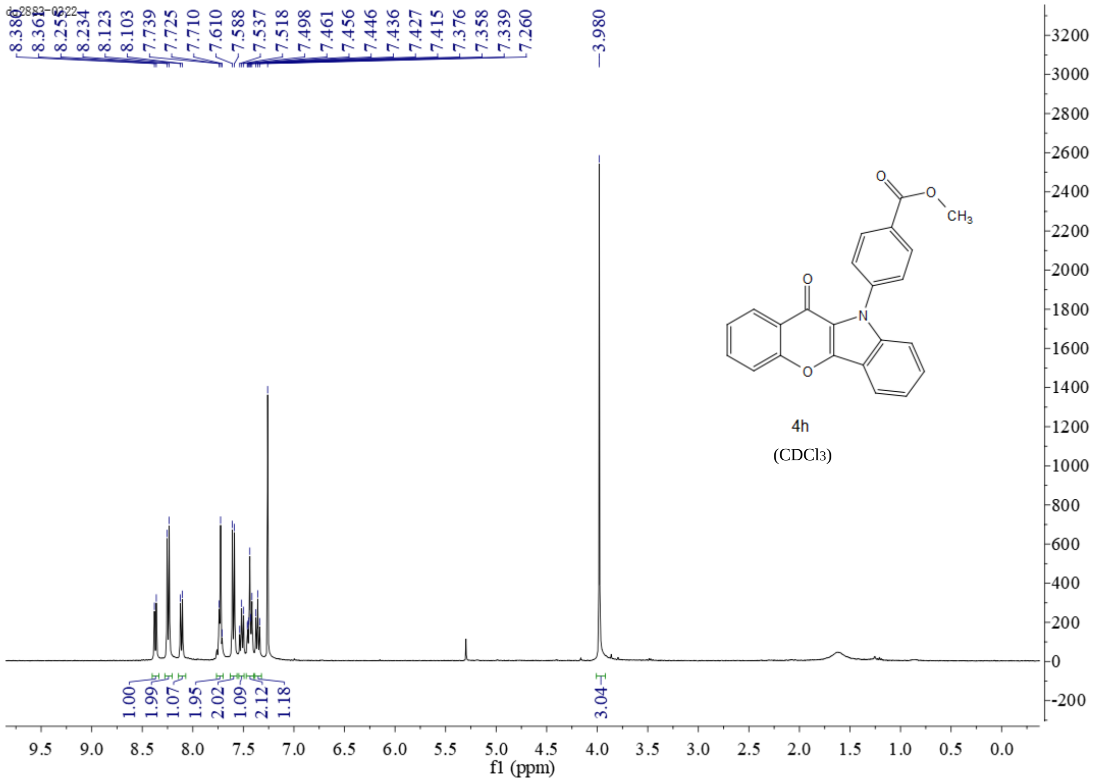
dq2883-0322
3.980
1.00
1.99
1.07
1.95
2.02
1.09
2.12
1.18
3.04
4h
(CDCl3)
f1 (ppm)

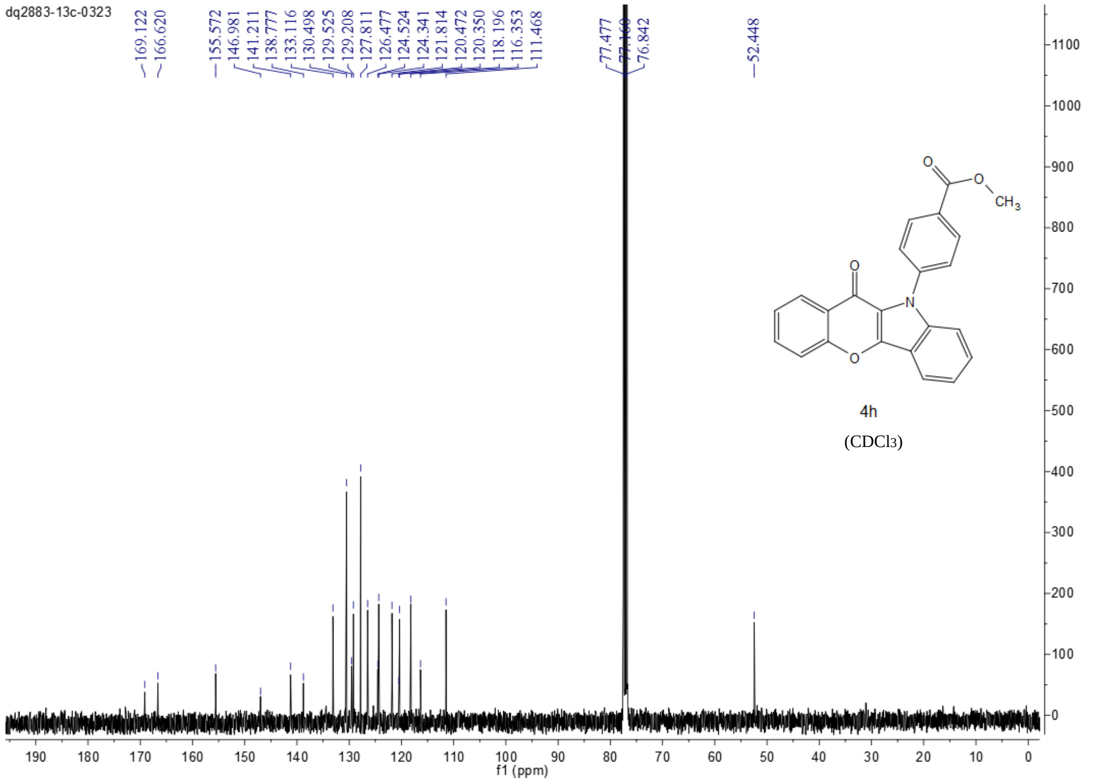
dq2883-13c-0323
169.122
166.620
155.572
146.981
141.211
138.777
133.116
130.498
129.525
129.208
127.811
126.477
124.524
124.341
121.814
120.472
120.350
118.196
116.353
111.468
77.477
76.842
52.448
4h
(CDCl3)
f1 (ppm)

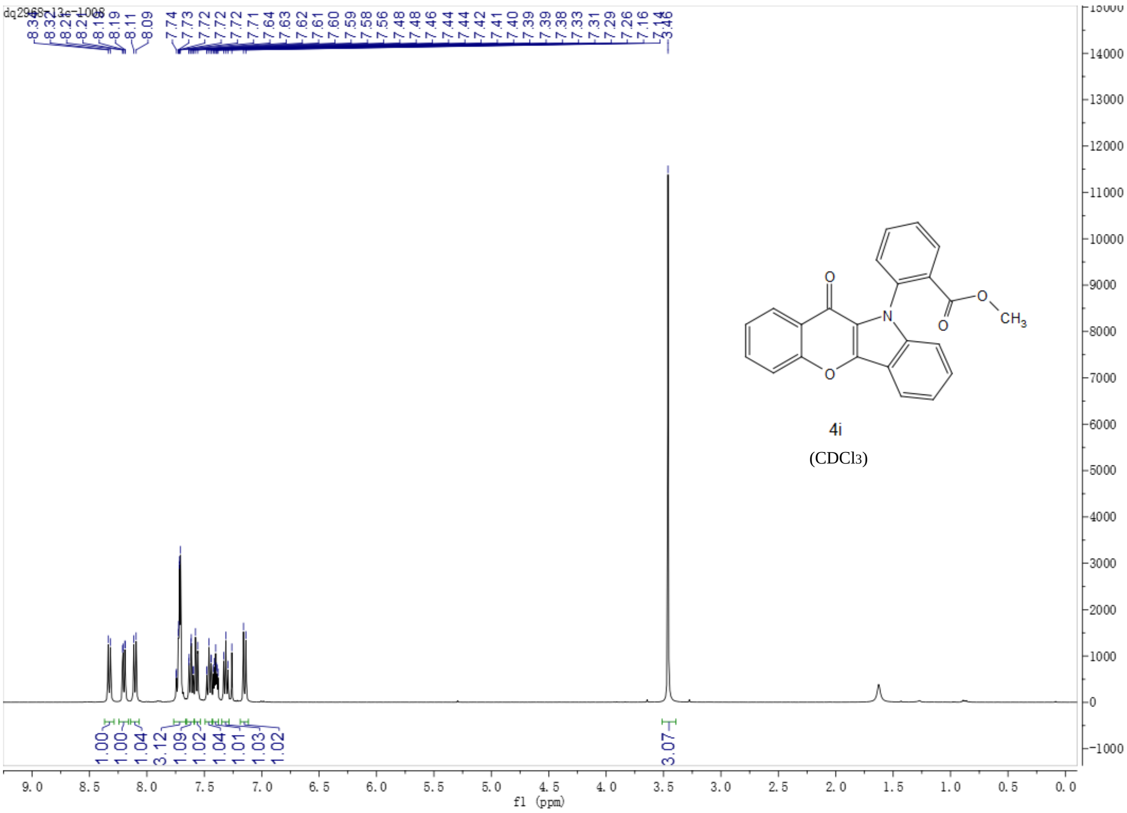
dq2968-13c-1008
8.11
8.09
3.46
1.00
1.00
1.04
3.12
1.09
1.02
1.04
1.01
1.03
1.02
3.07
4i
(CDCl3)
f1 (ppm)

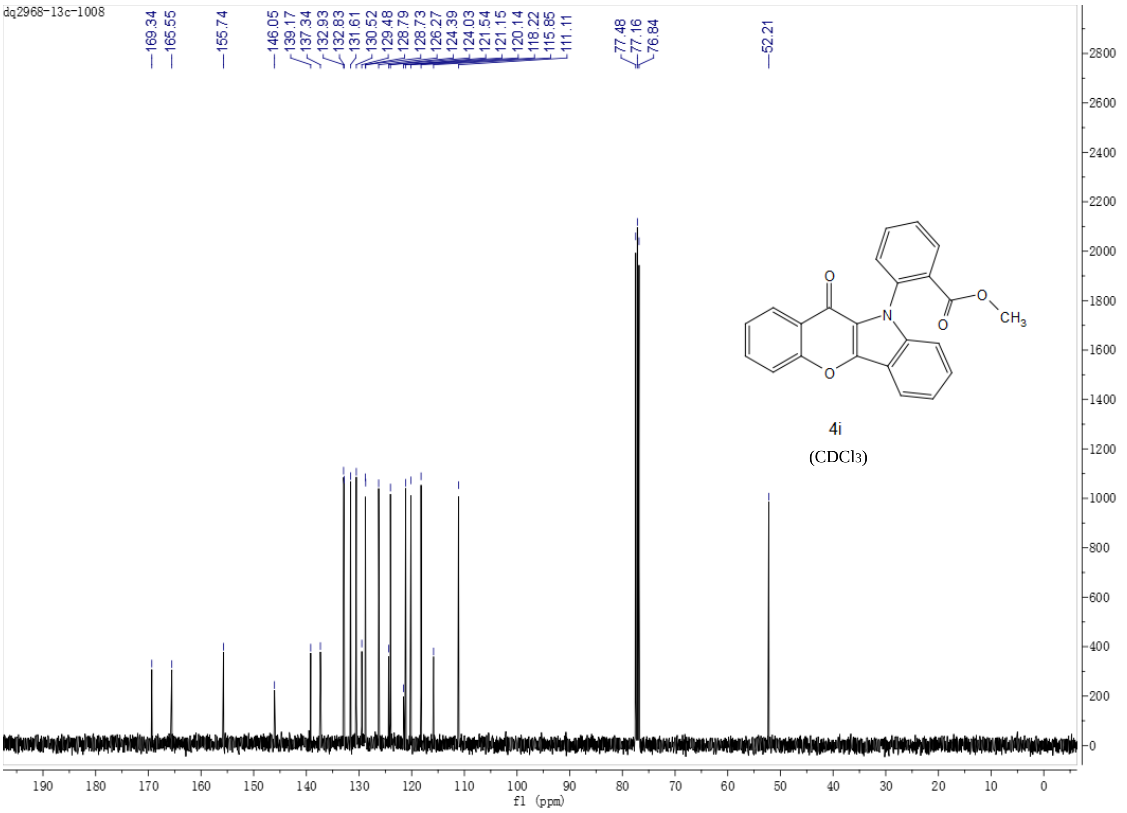
dq2968-13c-1008
169.34
165.55
155.74
146.05
139.17
137.34
132.93
132.83
131.61
130.52
129.48
128.79
128.73
126.27
124.39
124.03
121.54
121.15
120.14
118.22
115.85
111.11
77.48
77.16
76.84
52.21
4i
(CDCl3)
f1 (ppm)

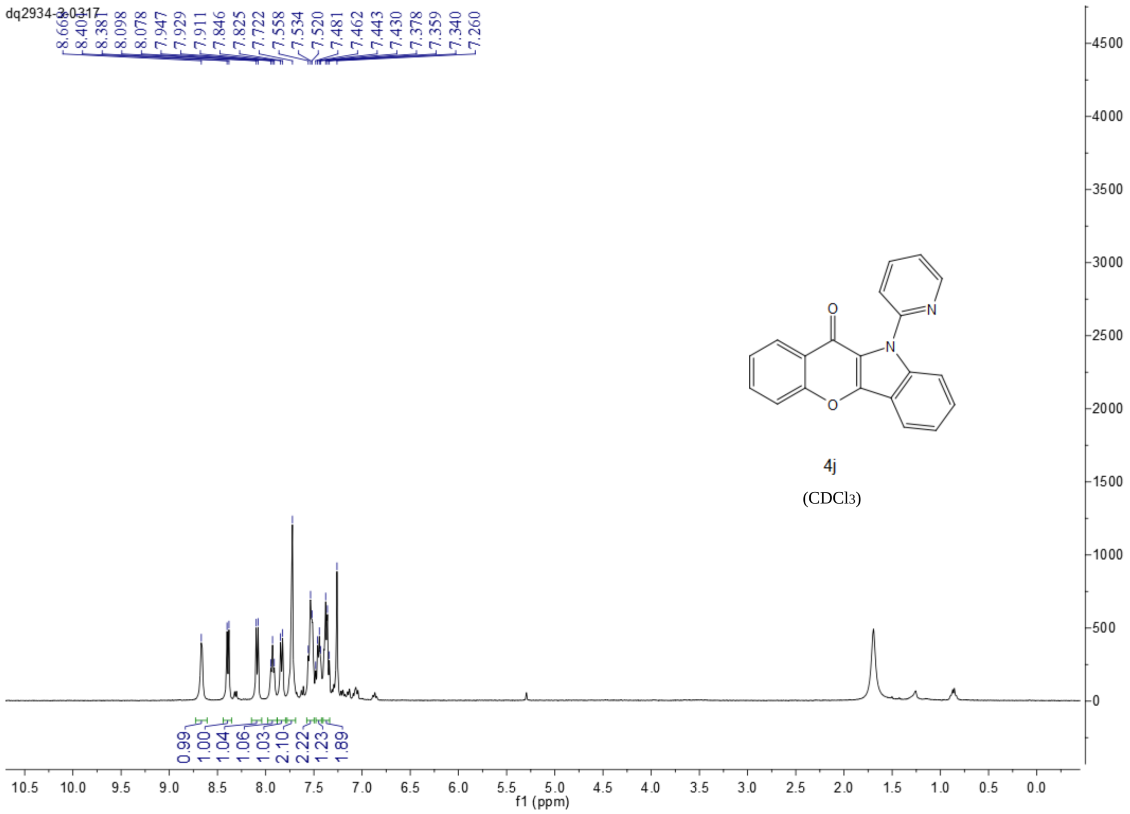
dq2934-3-0317
8.666
8.401
8.381
8.098
8.078
7.947
7.929
7.911
7.846
7.825
7.722
7.558
7.534
7.520
7.481
7.462
7.443
7.430
7.378
7.359
7.340
7.260
4j
(CDCl3)
0.99
1.00
1.04
1.06
1.03
2.10
2.22
1.23
1.89
f1 (ppm)

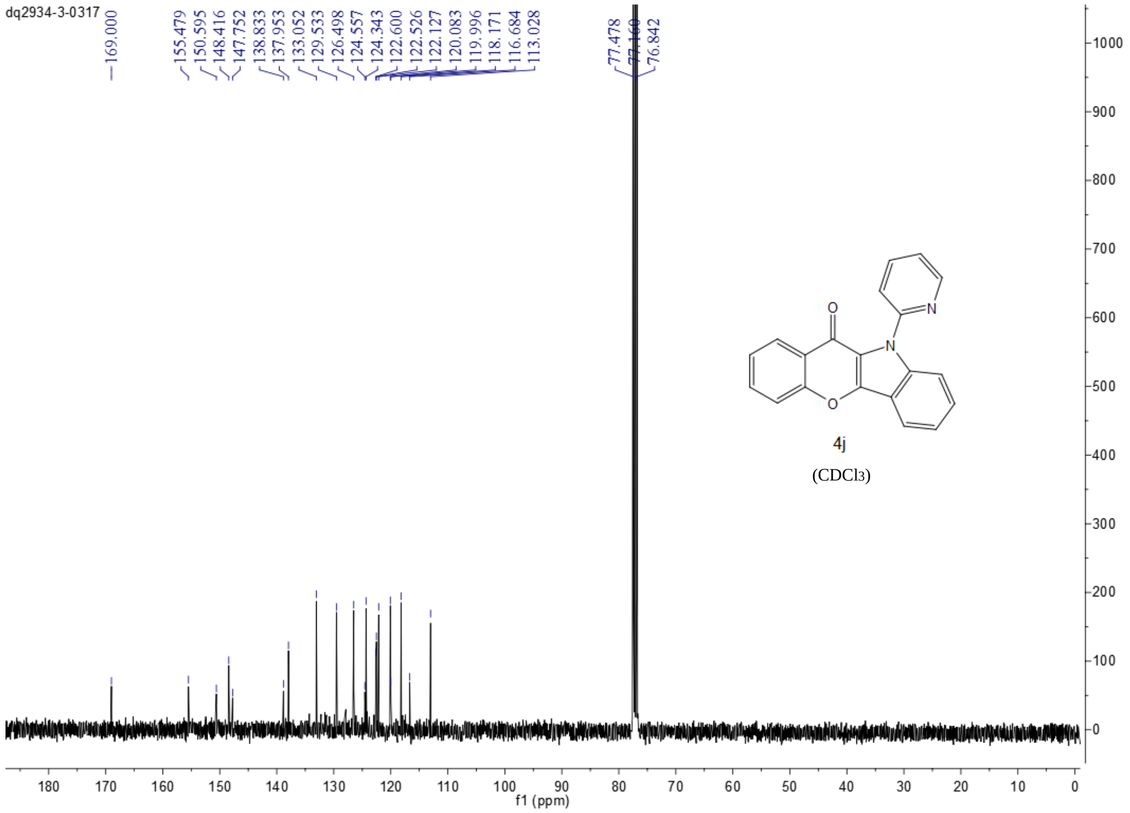
dq2934-3-0317
169.000
155.479
150.595
148.416
147.752
138.833
137.953
133.052
129.533
126.498
124.557
124.343
122.600
122.526
122.127
120.083
119.996
118.171
116.684
113.028
77.478
77.160
76.842
4j
(CDCl3)
f1 (ppm)

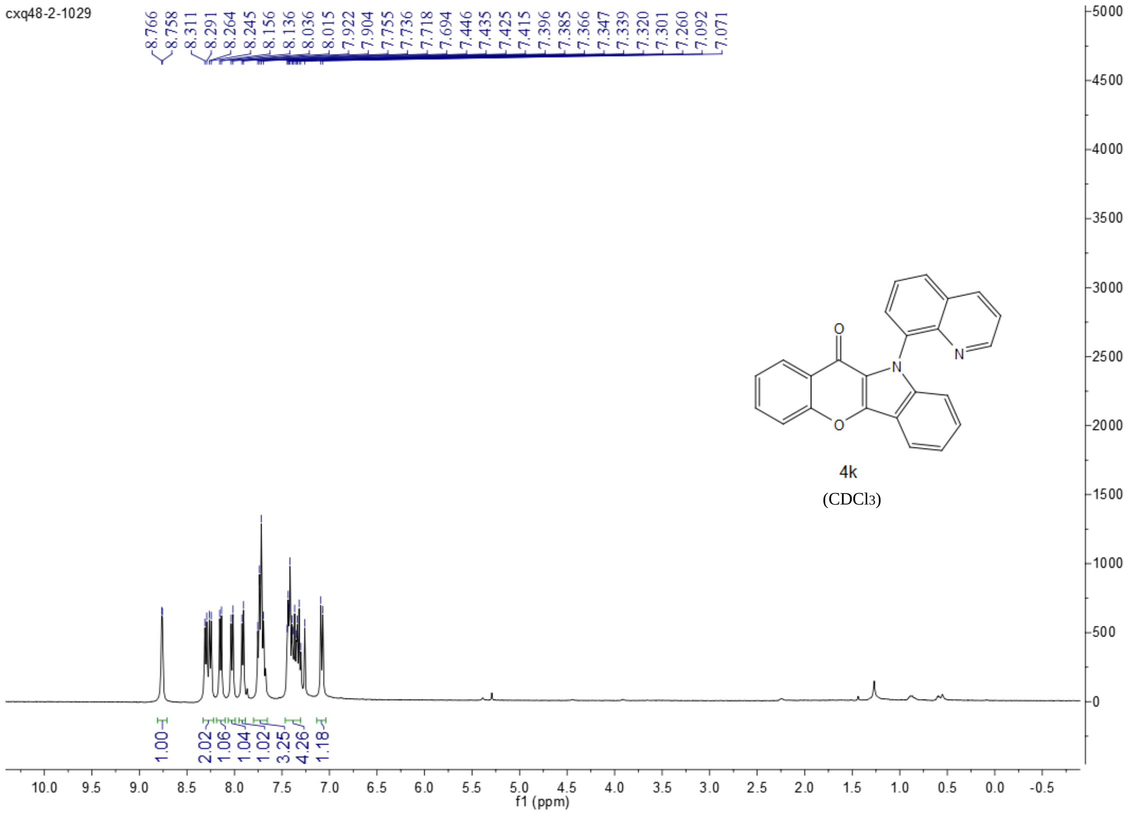
cxq48-2-1029
8.766 8.758 8.311 8.291 8.264 8.245 8.156 8.136 8.036 8.015 7.922 7.904 7.755 7.736 7.718 7.694 7.446 7.435 7.425 7.415 7.396 7.385 7.366 7.347 7.339 7.320 7.301 7.260 7.092 7.071
O
N
N
O
4k
(CDCl3)
1.00 2.02 1.06 1.04 1.02 3.25 4.26 1.18
f1 (ppm)

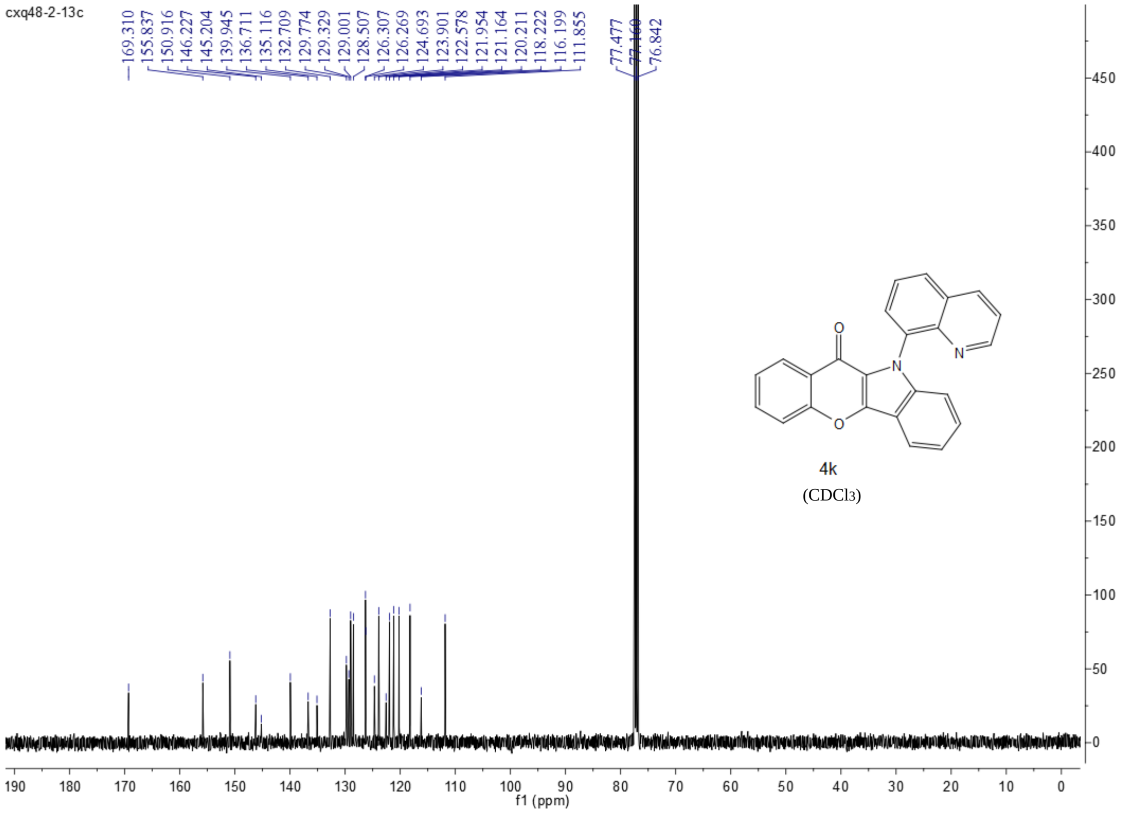
cxq48-2-13c
169.310 155.837 150.916 146.227 145.204 139.945 136.711 135.116 132.709 129.774 129.329 129.001 128.507 126.307 126.269 124.693 123.901 122.578 121.954 121.164 120.211 118.222 116.199 111.855
77.477 77.160 76.842
O
N
N
O
4k
(CDCl3)
f1 (ppm)

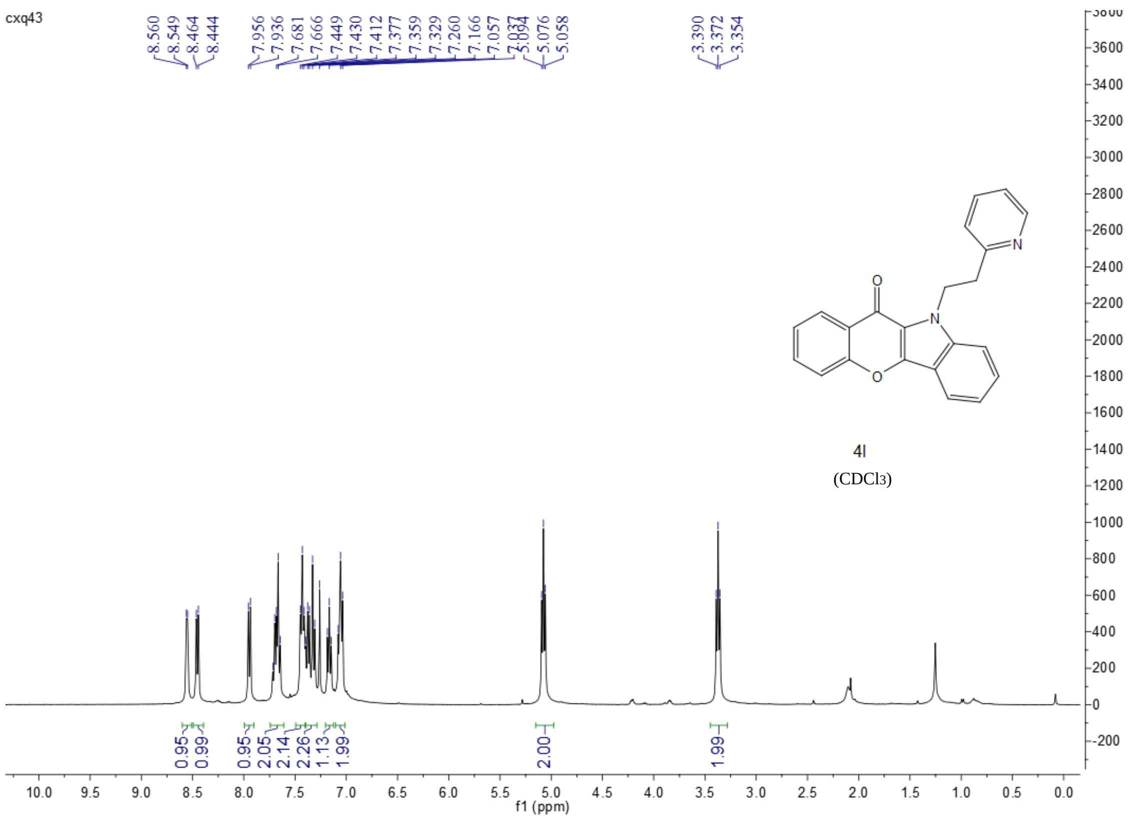

4l
(CDCl3)

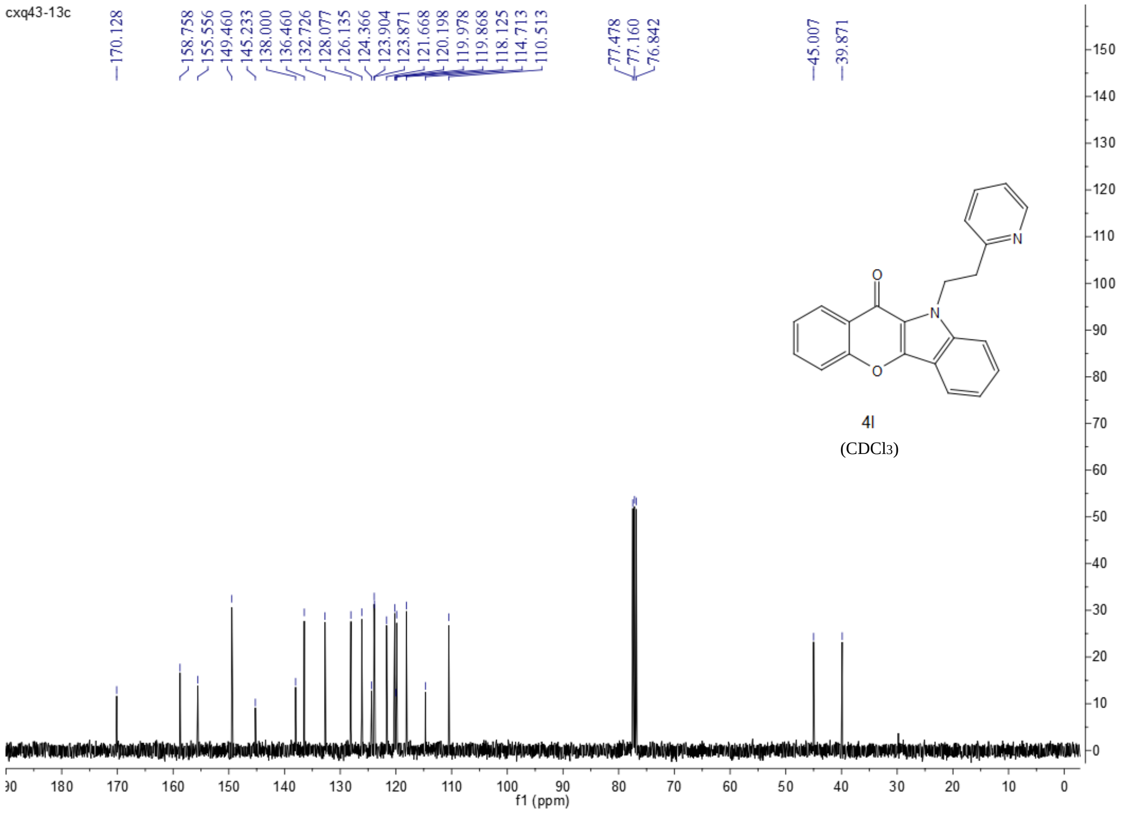

4l
(CDCl3)

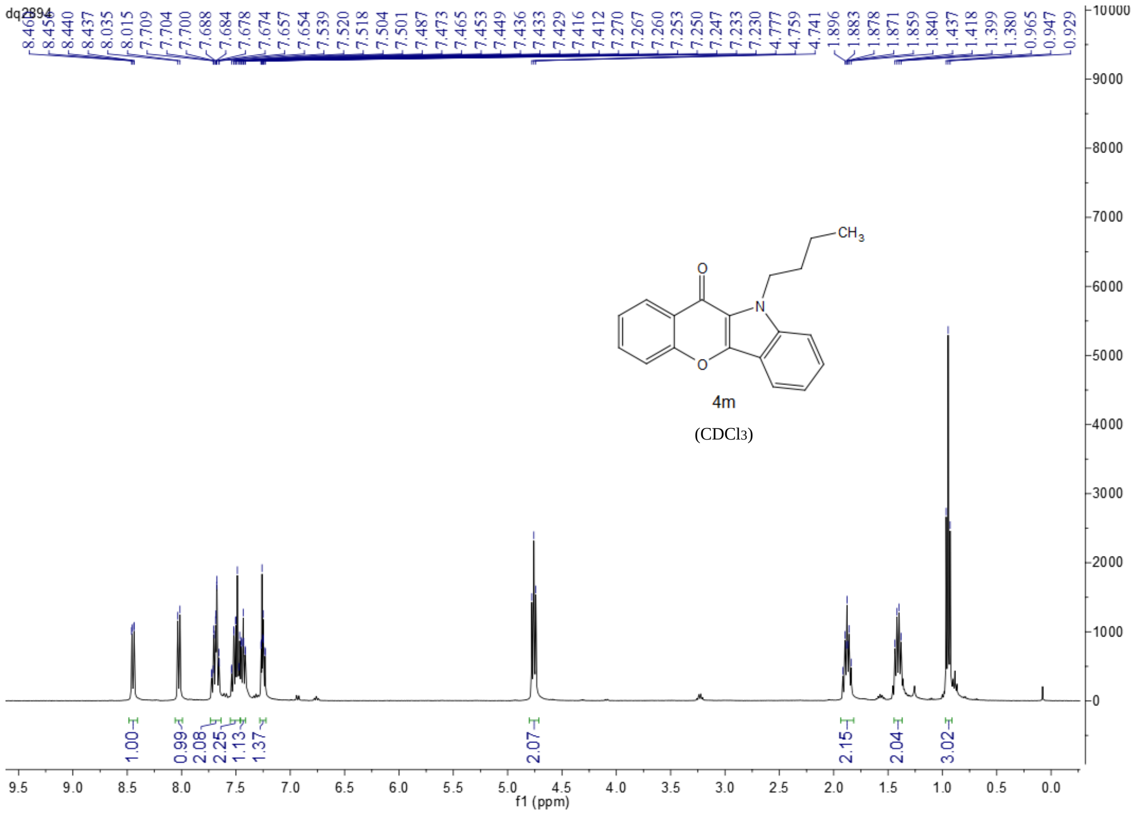
dq2894
8.459 8.456 8.440 8.437 8.035 8.015 7.709 7.704 7.700 7.688 7.684 7.678 7.674 7.657 7.654 7.539 7.520 7.518 7.504 7.501 7.487 7.473 7.465 7.453 7.449 7.436 7.433 7.429 7.416 7.412 7.270 7.267 7.260 7.253 7.250 7.247 7.233 7.230 4.777 4.759 4.741 1.896 1.883 1.878 1.871 1.859 1.840 1.437 1.418 1.399 1.380 0.965 0.947 0.929
CH_3
O
N
O
4m
(CDCl3)
1.00 0.99 2.08 2.25 1.13 1.37 2.07 2.15 2.04 3.02
f1 (ppm)

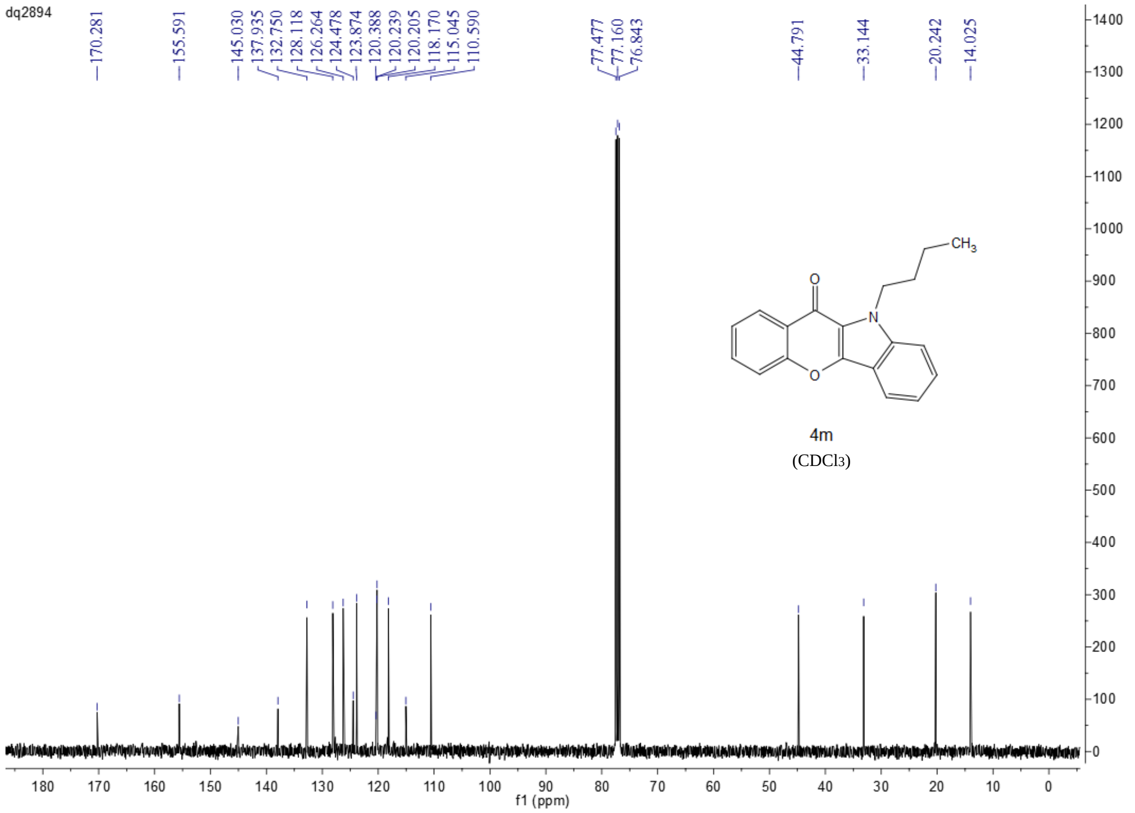
dq2894
170.281 155.591 145.030 137.935 132.750 128.118 126.264 124.478 123.874 120.388 120.239 120.205 118.170 115.045 110.590 77.477 77.160 76.843 44.791 33.144 20.242 14.025
CH_3
O
N
O
4m
(CDCl3)
f1 (ppm)

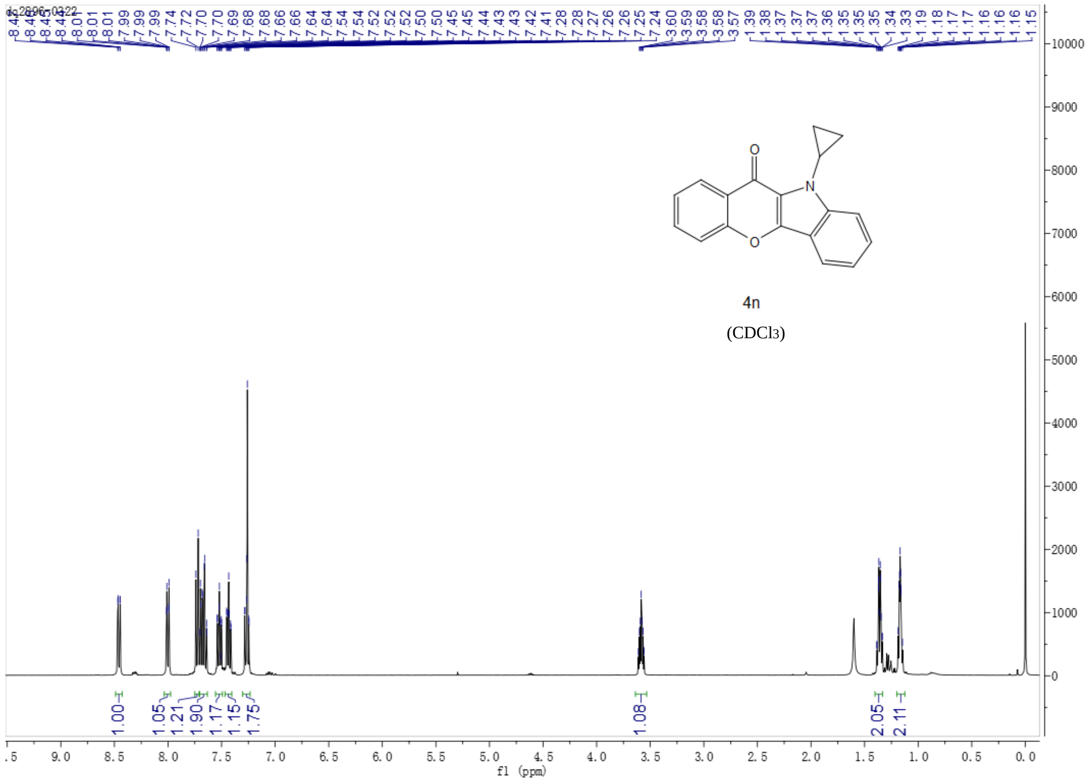

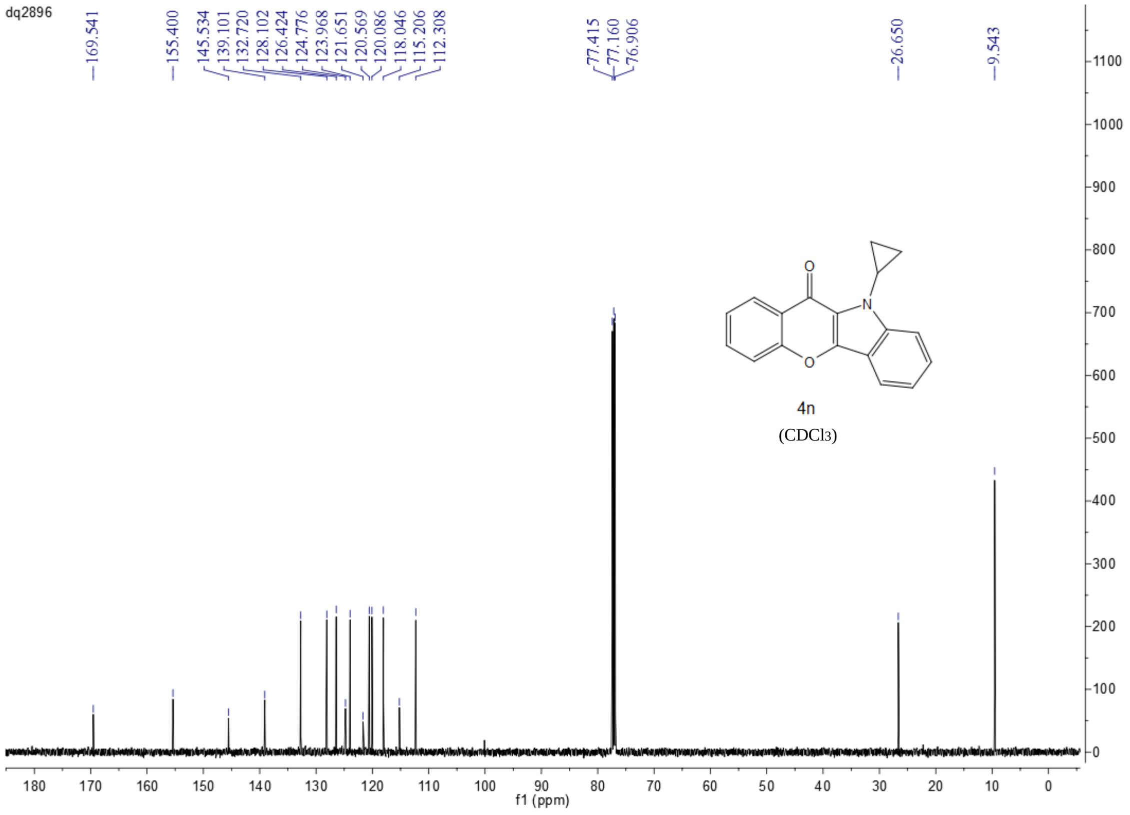

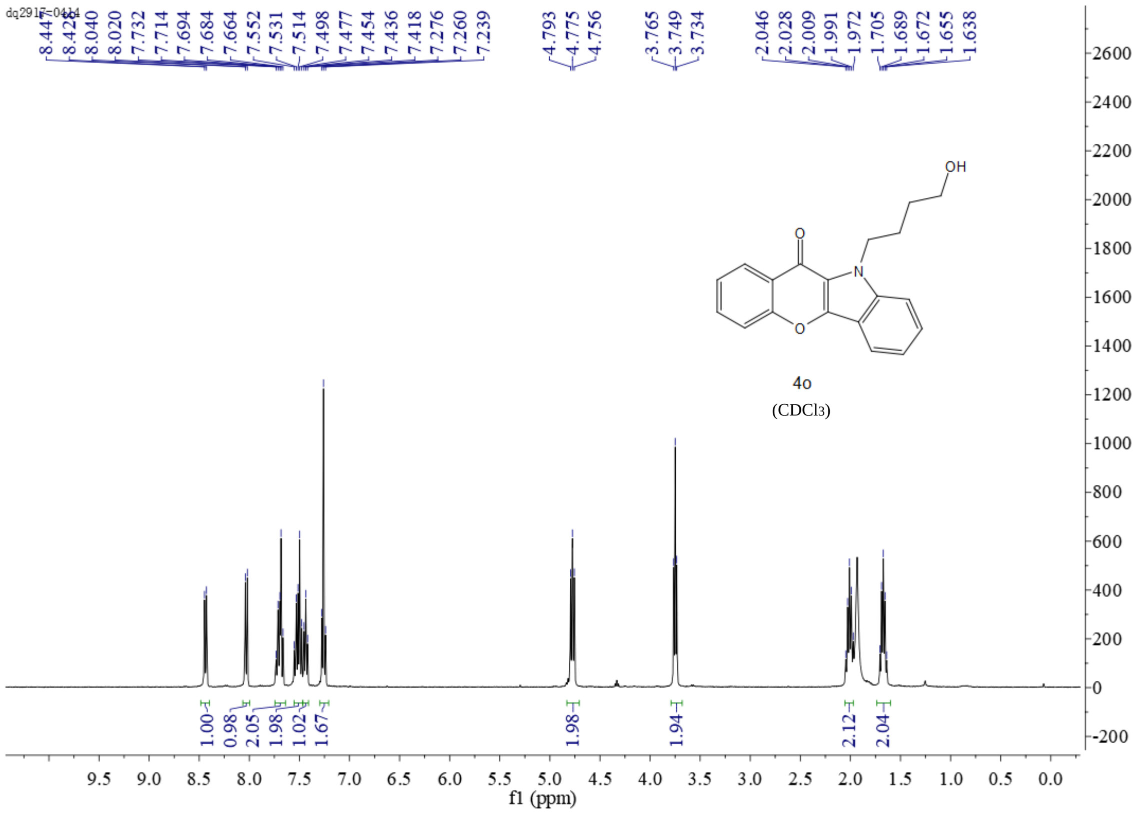
dq2917-0414
8.444
8.428
8.040
8.020
7.732
7.714
7.694
7.684
7.664
7.552
7.531
7.514
7.498
7.477
7.454
7.436
7.418
7.276
7.260
7.239
4.793
4.775
4.756
3.765
3.749
3.734
2.046
2.028
2.009
1.991
1.972
1.705
1.689
1.672
1.655
1.638
OH
O
N
O
4o
(CDCl3)
1.00
0.98
2.05
1.98
1.02
1.67
1.98
1.94
2.12
2.04
f1 (ppm)

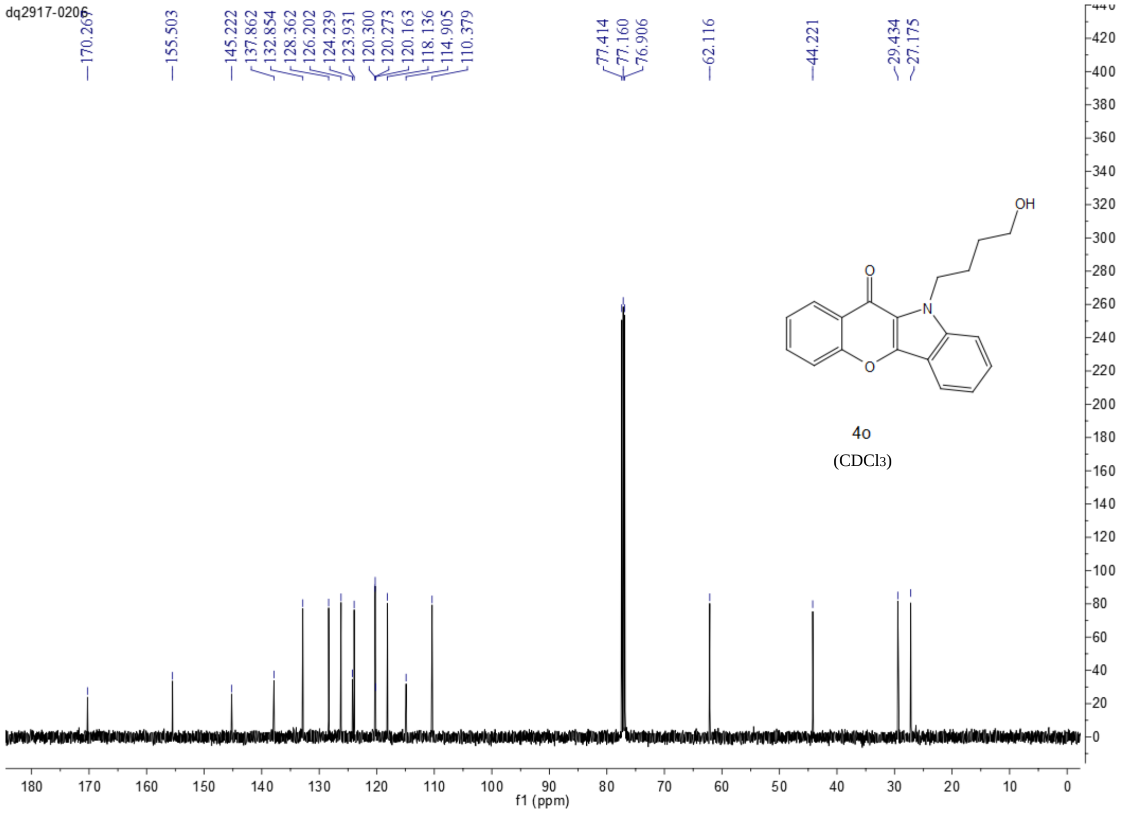
dq2917-0206
170.267
155.503
145.222
137.862
132.854
128.362
126.202
124.239
123.931
120.300
120.273
120.163
118.136
114.905
110.379
77.414
77.160
76.906
62.116
44.221
29.434
27.175
OH
O
N
O
4o
(CDCl3)
f1 (ppm)

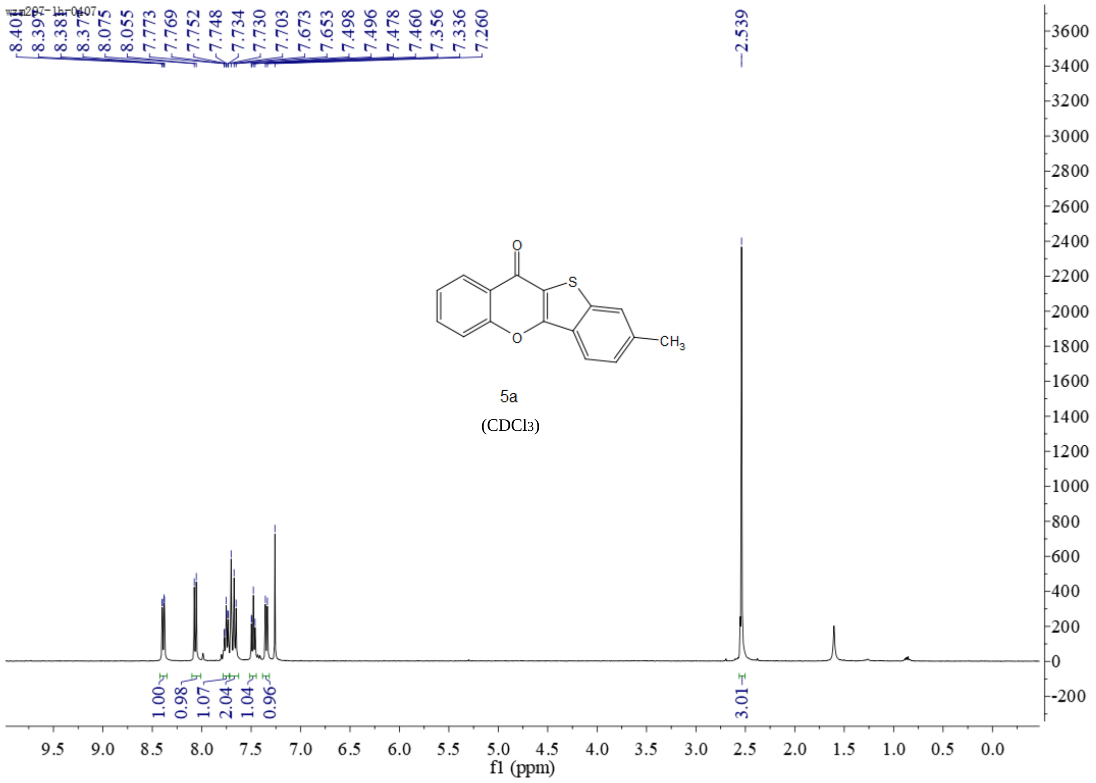
wzm297-1hr-0407
8.401
8.397
8.381
8.377
8.075
8.055
7.773
7.769
7.752
7.748
7.734
7.730
7.703
7.673
7.653
7.498
7.496
7.478
7.460
7.356
7.336
7.260
2.539
O
S
O
CH3
5a
(CDCl3)
1.00
0.98
1.07
2.04
1.04
0.96
3.01
f1 (ppm)

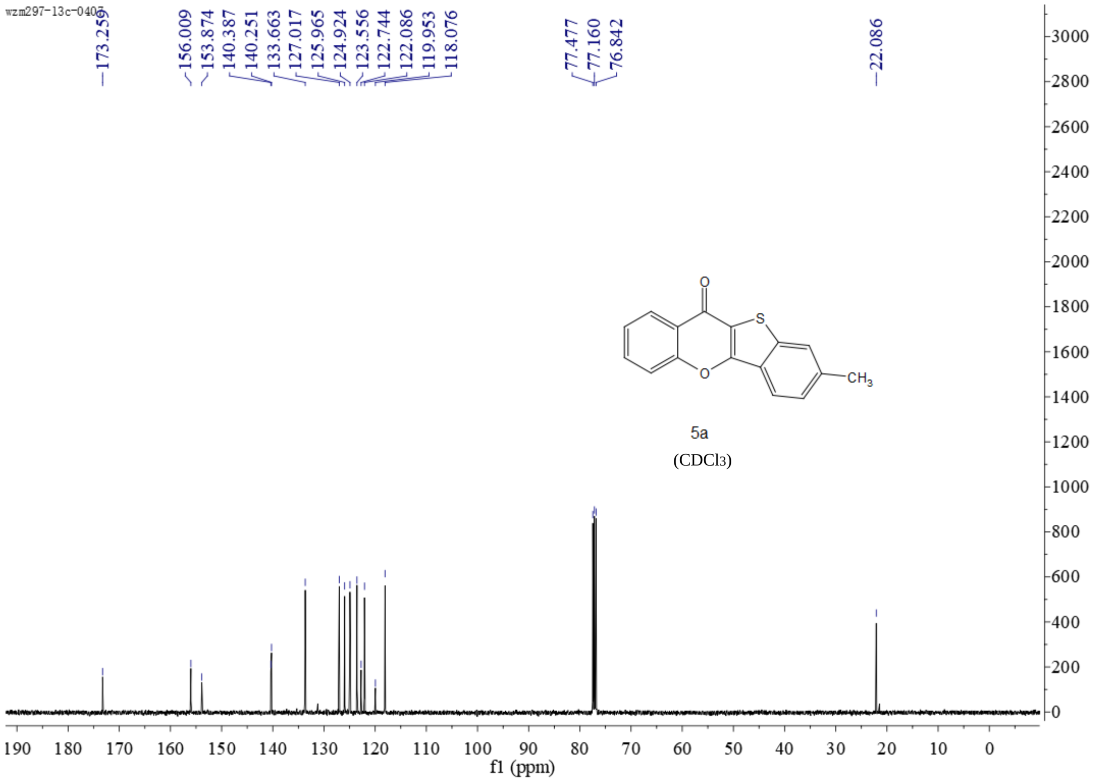
wzm297-13c-0407
173.259
156.009
153.874
140.387
140.251
133.663
127.017
125.965
124.924
123.556
122.744
122.086
119.953
118.076
77.477
77.160
76.842
22.086
O
S
O
CH3
5a
(CDCl3)
f1 (ppm)

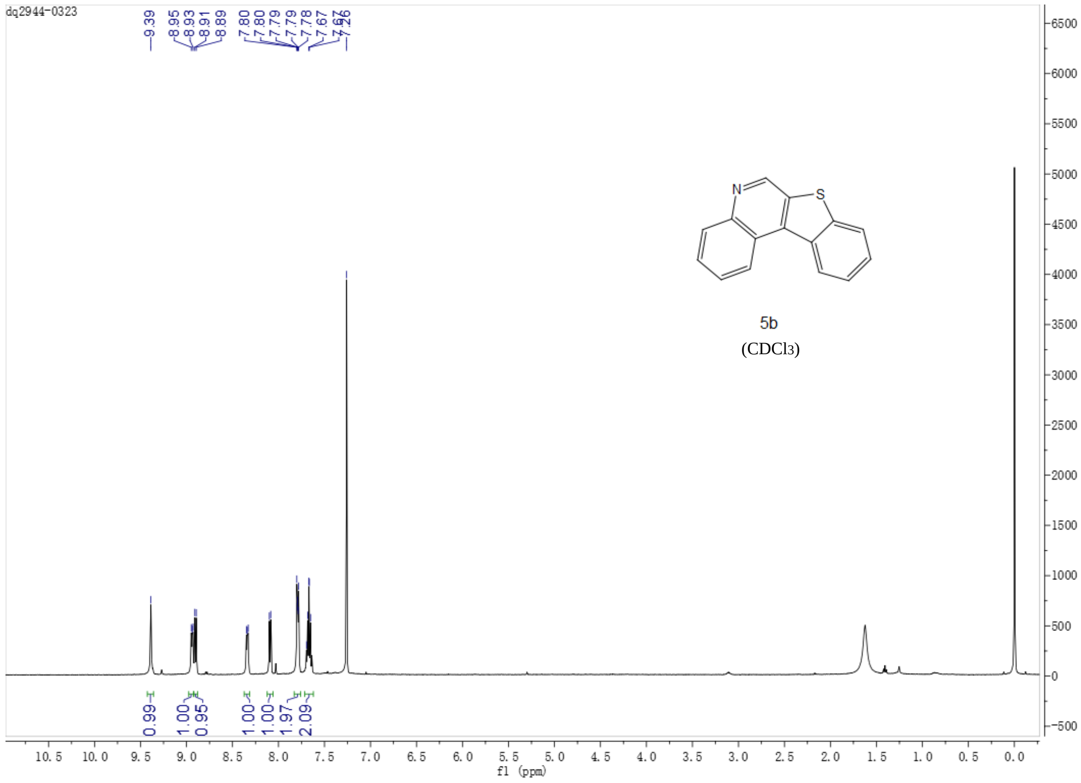
dq2944-0323
9.39
8.95
8.93
8.91
8.89
7.80
7.80
7.79
7.79
7.78
7.67
7.67
7.26
0.99
1.00
0.95
1.00
1.00
1.97
2.09
N
S
5b
(CDCl3)
f1 (ppm)

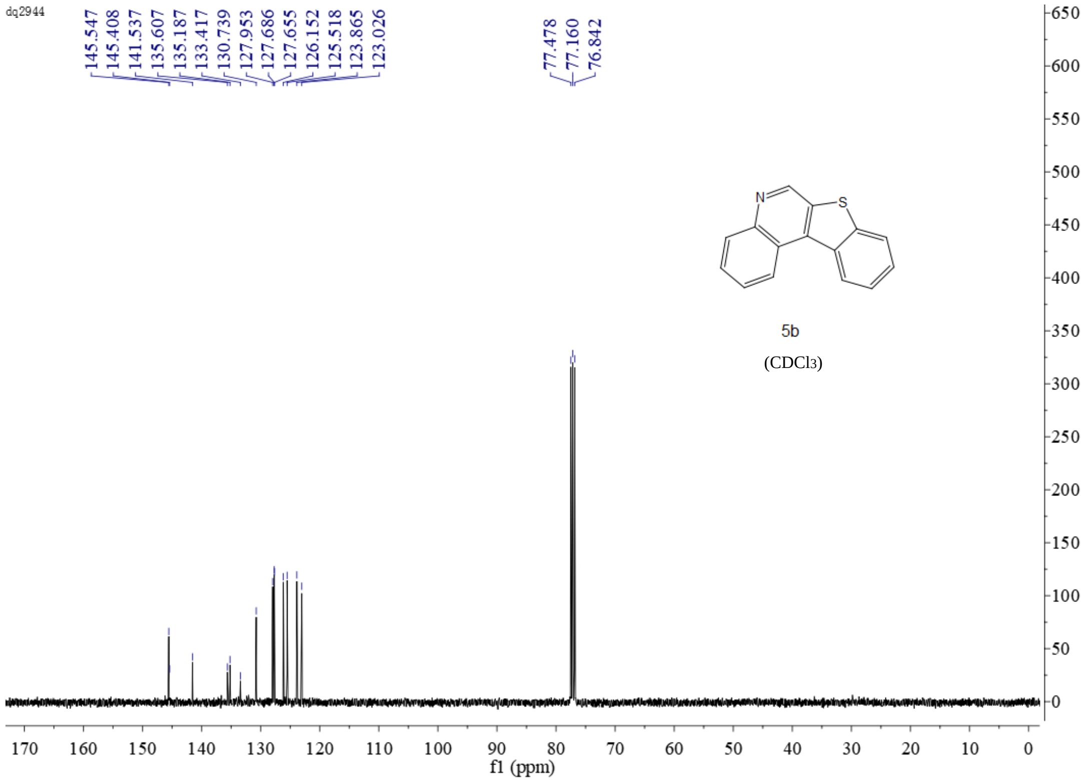
dq2944
145.547
145.408
141.537
135.607
135.187
133.417
130.739
127.953
127.686
127.655
126.152
125.518
123.865
123.026
77.478
77.160
76.842
N
S
5b
(CDCl3)
f1 (ppm)

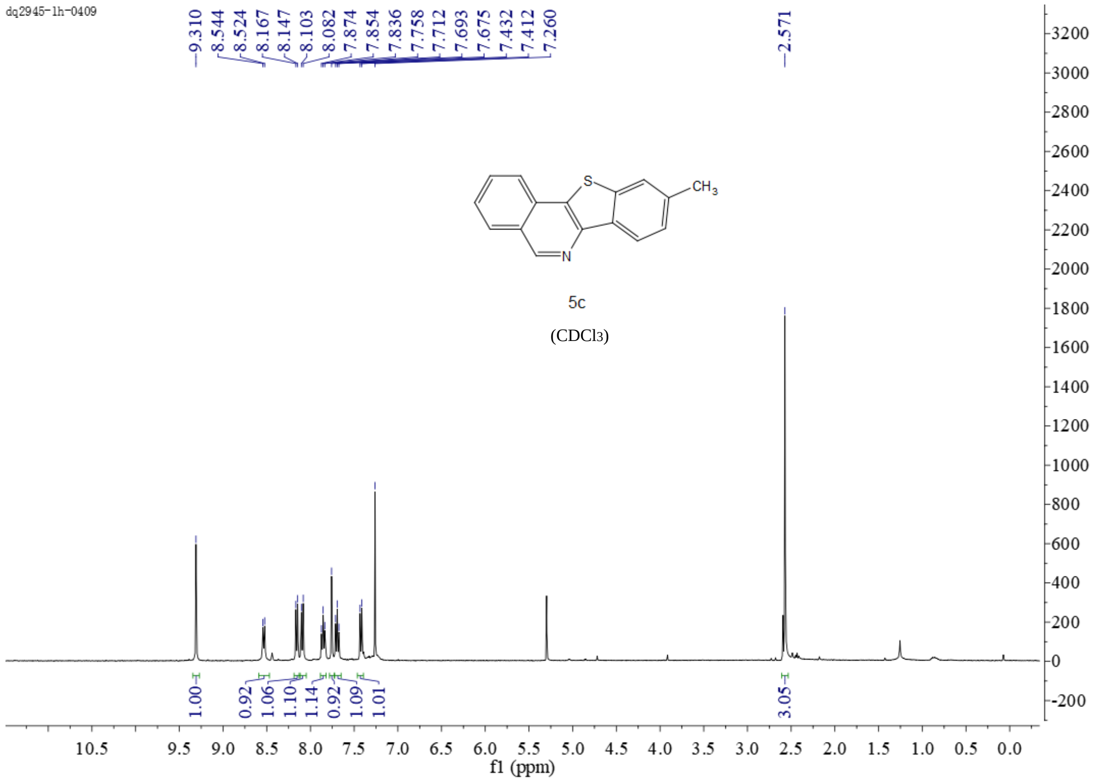

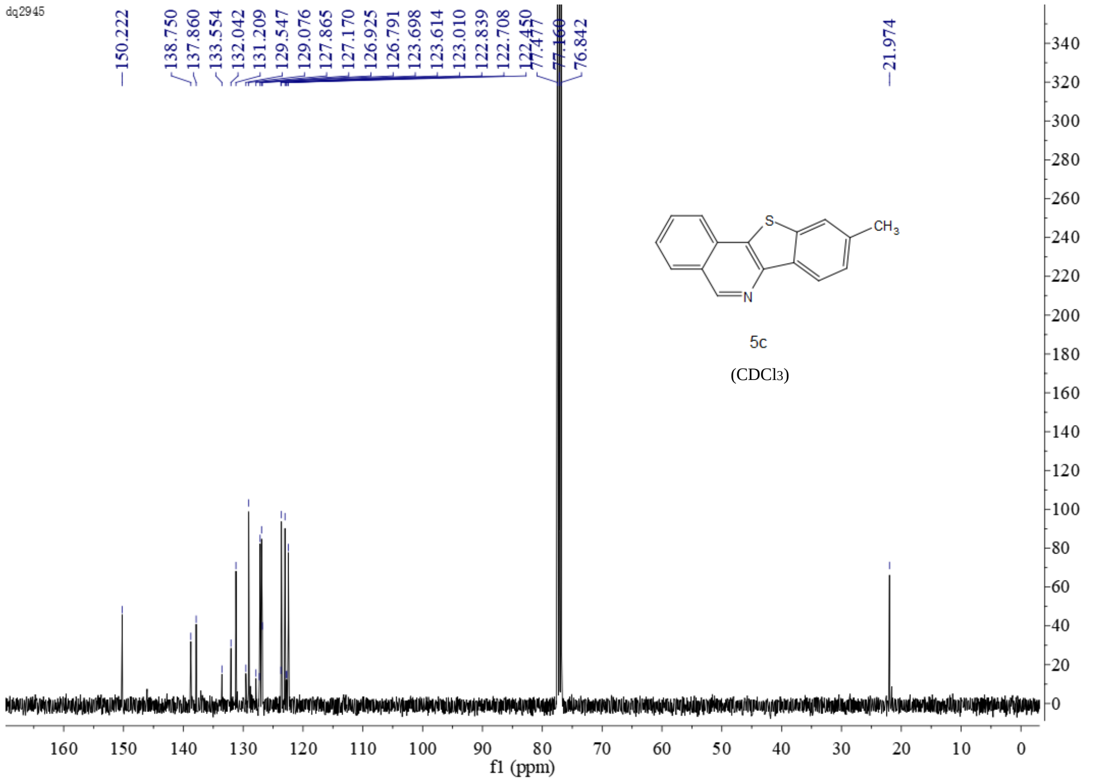

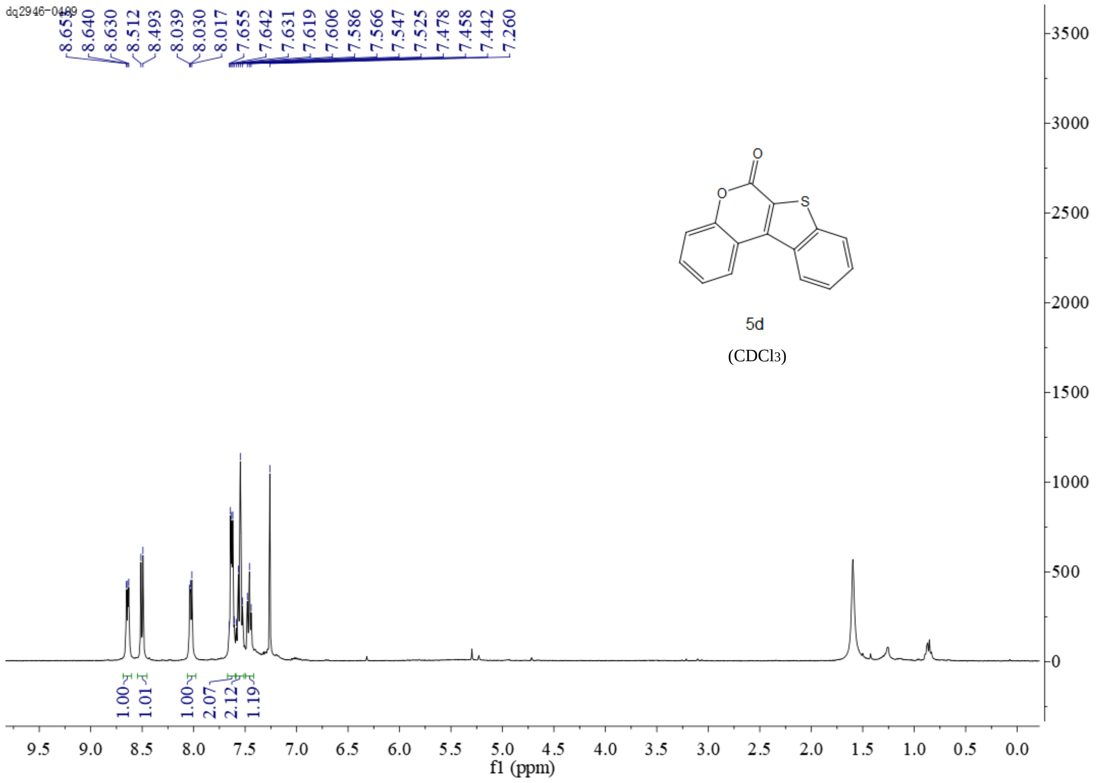
dq2946-0409
8.655
8.640
8.630
8.512
8.493
8.039
8.030
8.017
7.655
7.642
7.631
7.619
7.606
7.586
7.566
7.547
7.525
7.478
7.458
7.442
7.260
O
O
S
5d
(CDCl3)
1.00
1.01
1.00
2.07
2.12
1.19
f1 (ppm)

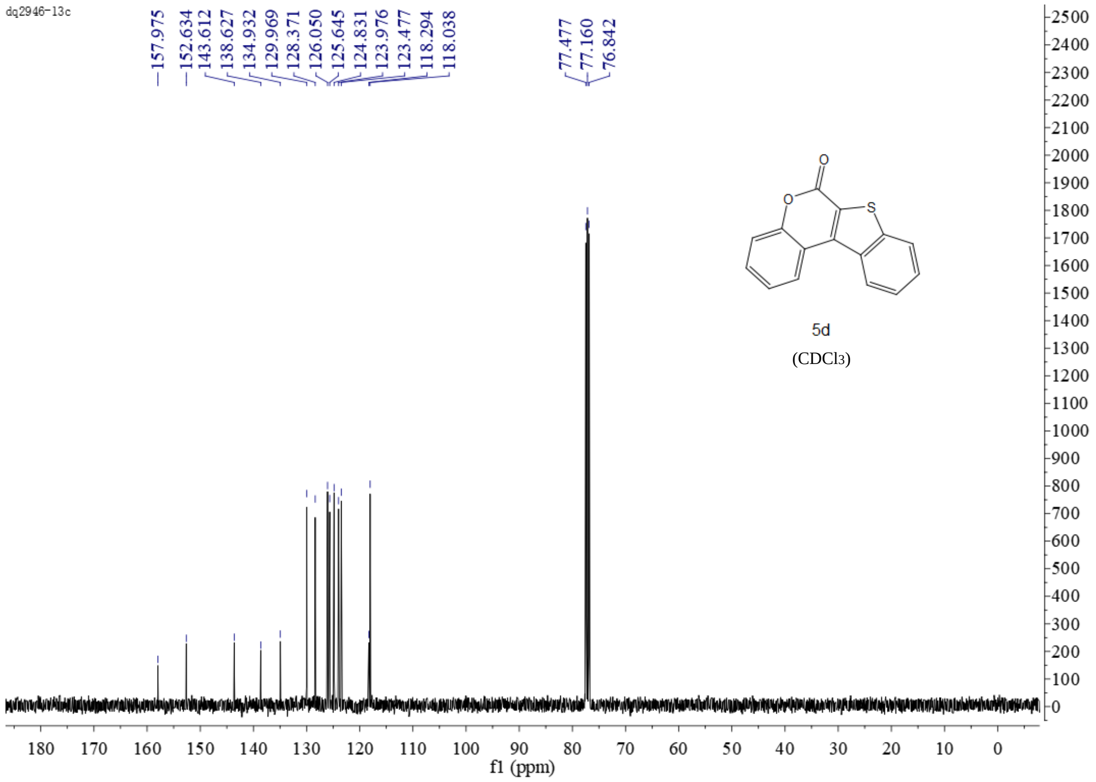
dq2946-13c
157.975
152.634
143.612
138.627
134.932
129.969
128.371
126.050
125.645
124.831
123.976
123.477
118.294
118.038
77.477
77.160
76.842
O
O
S
5d
(CDCl3)
f1 (ppm)

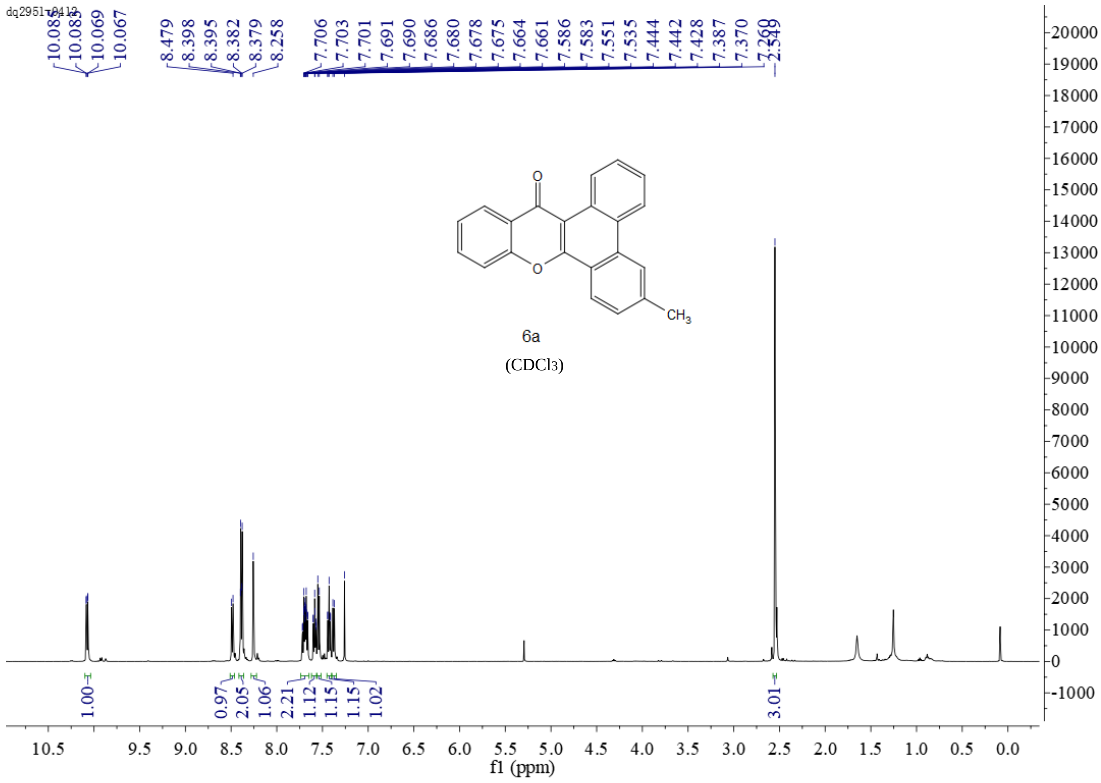
dq2951-0412
10.087
10.085
10.069
10.067
8.479
8.398
8.395
8.382
8.379
8.258
7.706
7.703
7.701
7.691
7.690
7.686
7.680
7.678
7.675
7.664
7.661
7.586
7.583
7.551
7.535
7.444
7.442
7.428
7.387
7.370
7.260
2.549
6a
(CDCl3)
1.00
0.97
2.05
1.06
2.21
1.12
1.15
1.15
1.02
3.01
f1 (ppm)

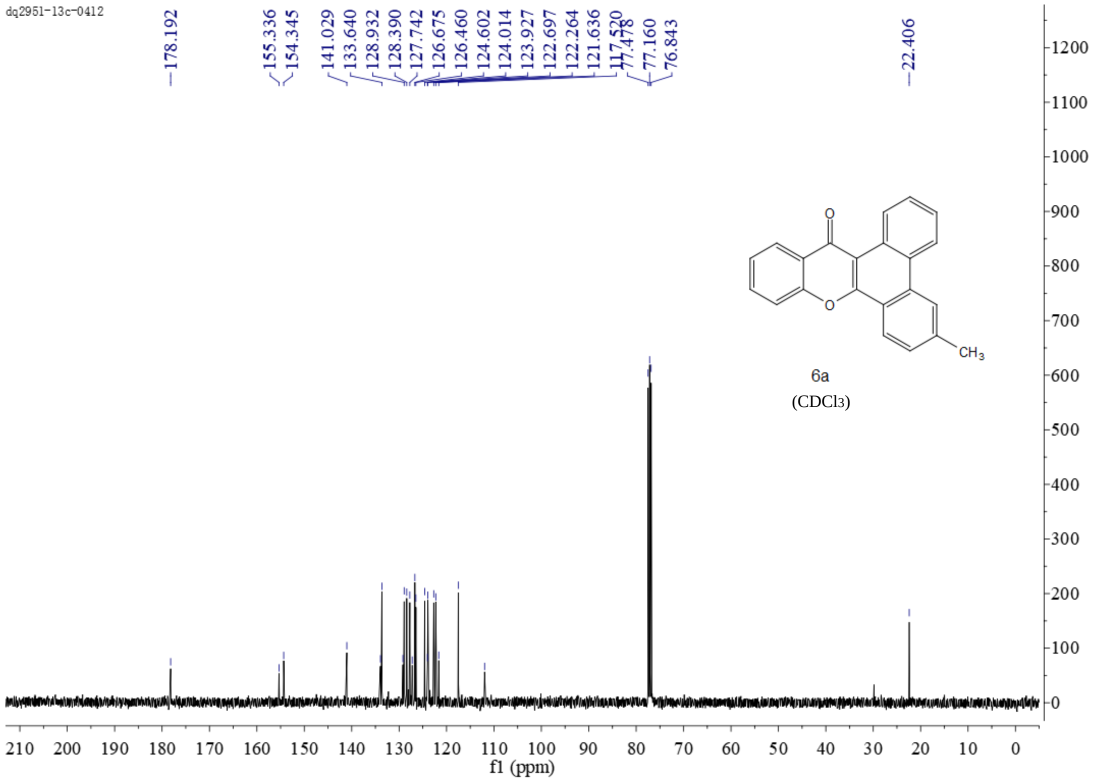
dq2951-13c-0412
178.192
155.336
154.345
141.029
133.640
128.932
128.390
127.742
126.675
126.460
124.602
124.014
123.927
122.697
122.264
121.636
117.520
77.478
77.160
76.843
22.406
6a
(CDCl3)
f1 (ppm)

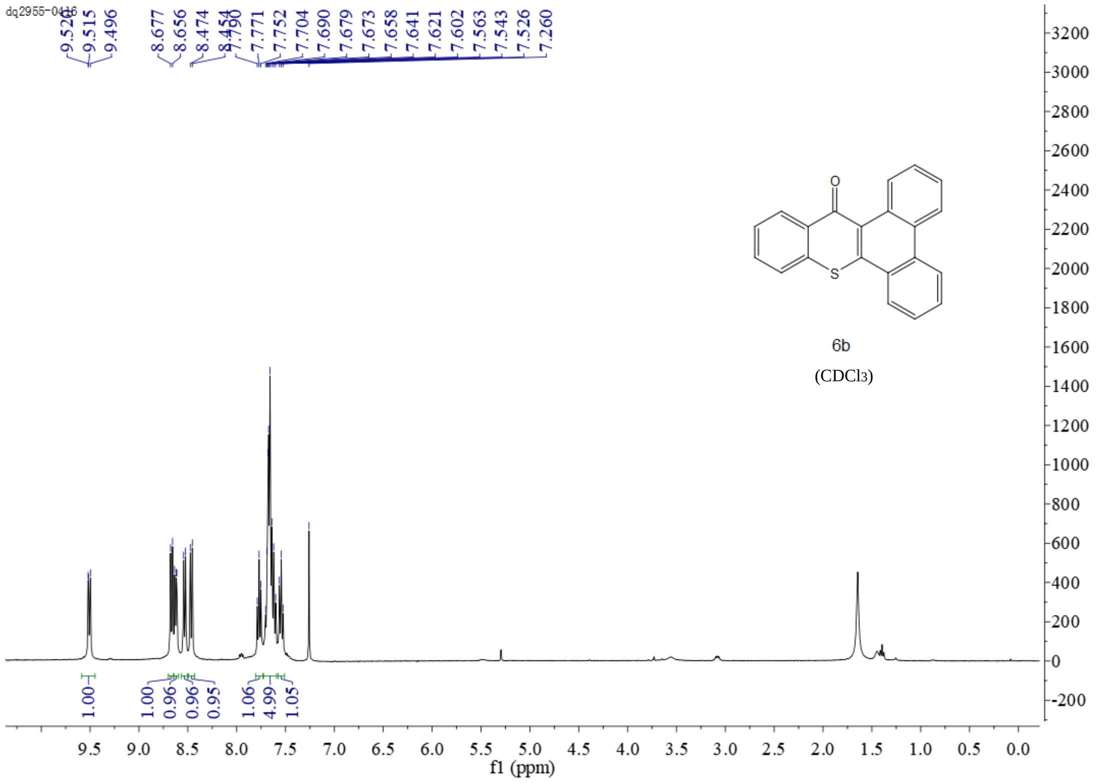

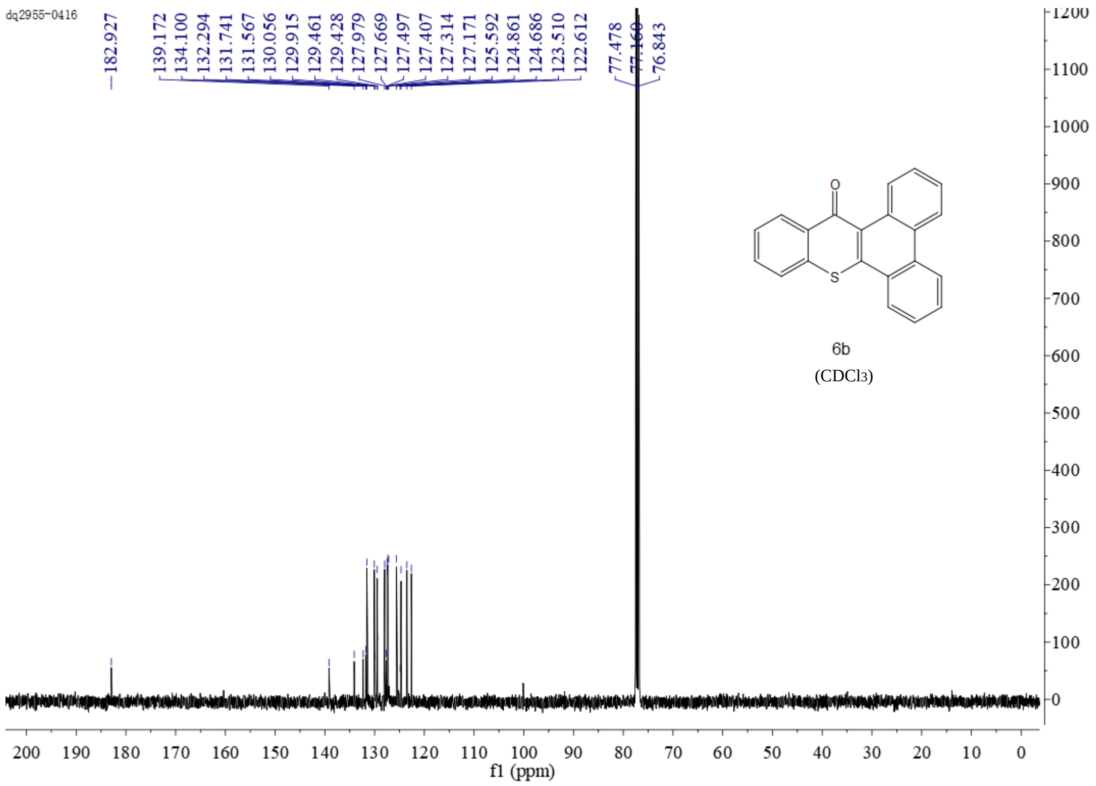

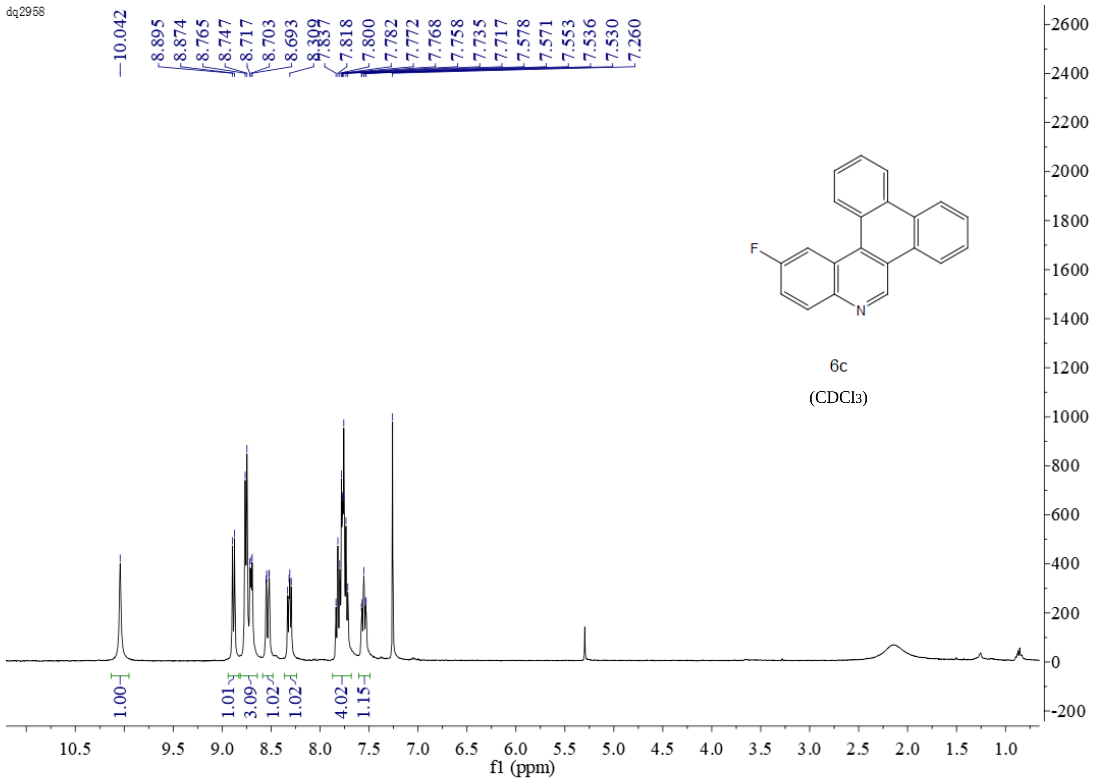
dq2958
10.042
8.895
8.874
8.765
8.747
8.717
8.703
8.693
8.309
7.837
7.818
7.800
7.782
7.772
7.768
7.758
7.735
7.717
7.578
7.571
7.553
7.536
7.530
7.260
F
N
6c
(CDCl3)
1.00
1.01
3.09
1.02
1.02
4.02
1.15
f1 (ppm)

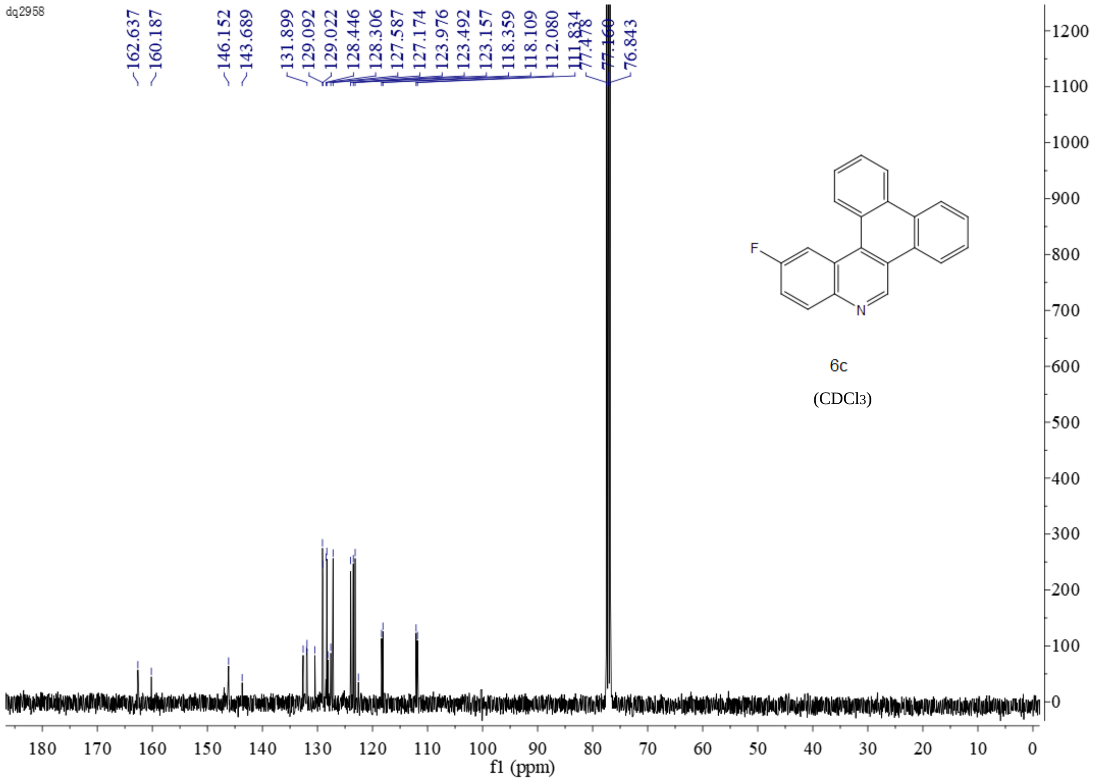
dq2958
162.637
160.187
146.152
143.689
131.899
129.092
129.022
128.446
128.306
127.587
127.174
123.976
123.492
123.157
118.359
118.109
112.080
111.834
77.478
77.160
76.843
F
N
6c
(CDCl3)
f1 (ppm)

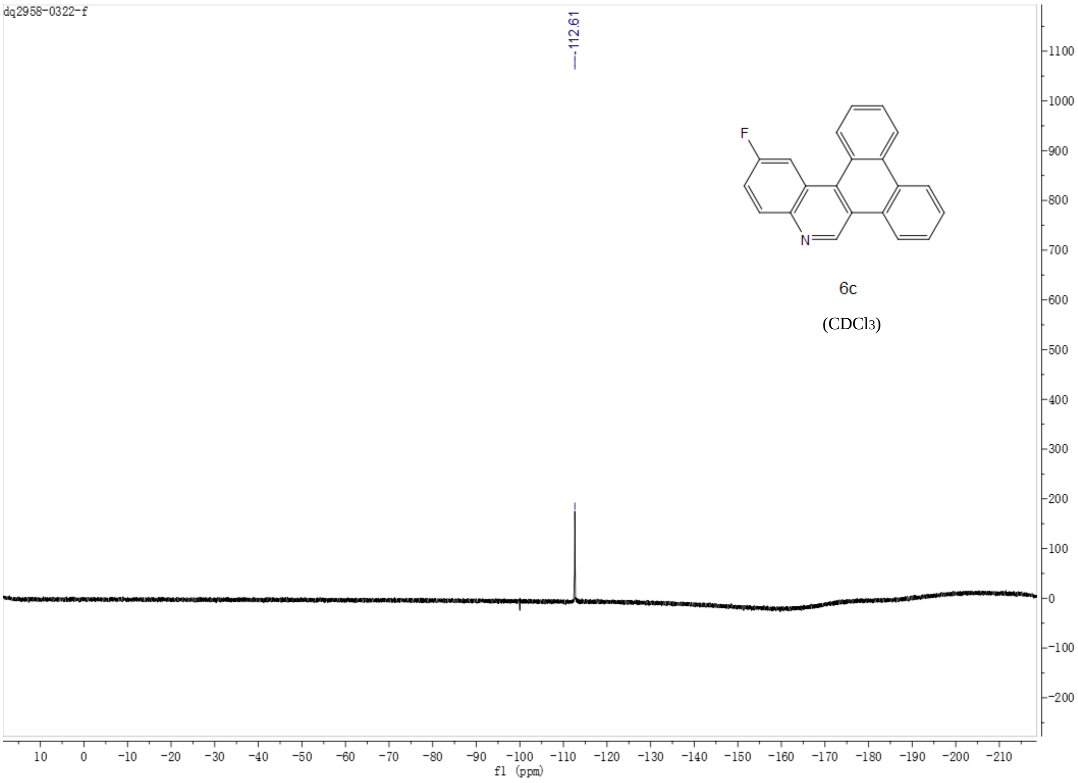
dq2958-0322-f
-112.61
6c
(CDCl3)
f1 (ppm)

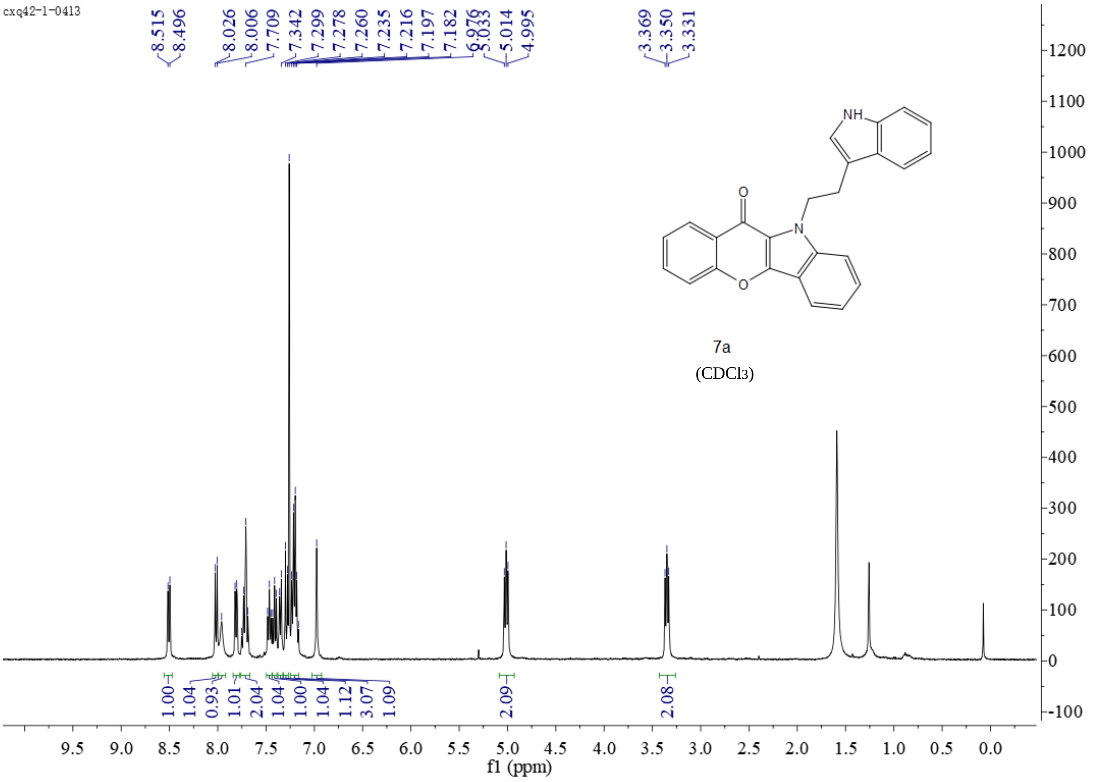
cxq42-1-0413
7a
(CDCl3)
f1 (ppm)

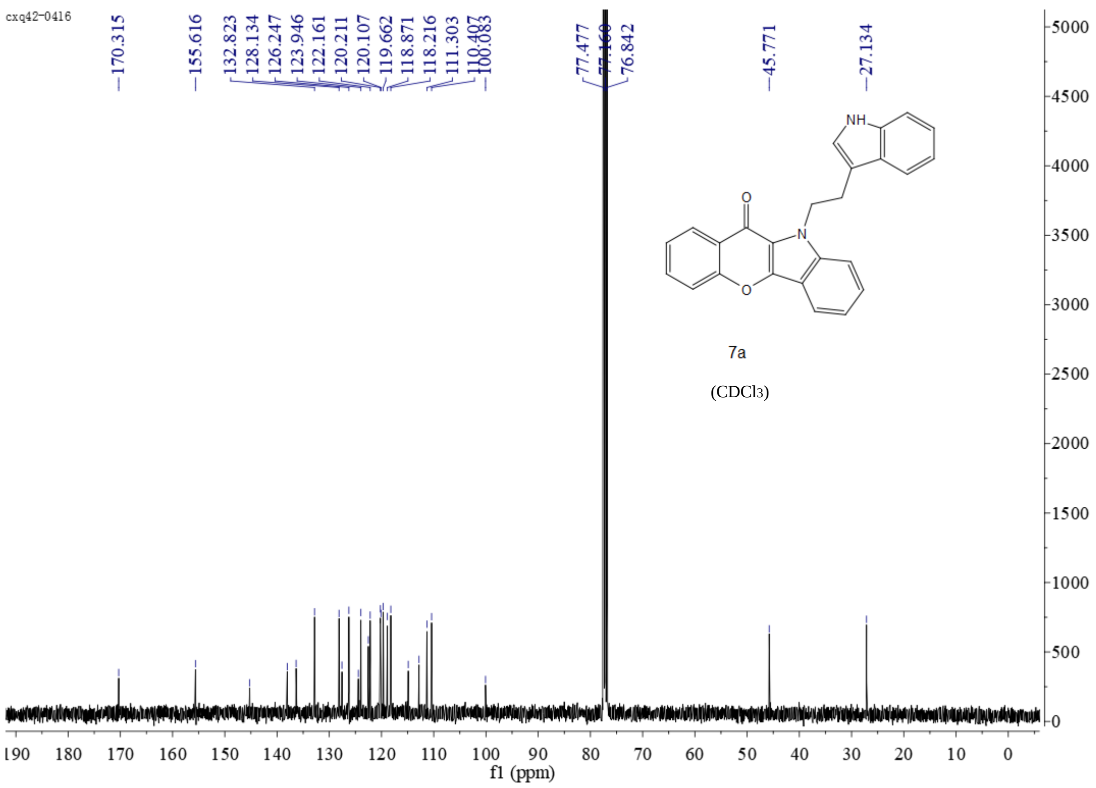
cxq42-0416
170.315
155.616
132.823
128.134
126.247
123.946
122.161
120.211
120.107
119.662
118.871
118.216
111.303
110.407
100.083
77.477
77.160
76.842
45.771
27.134
NH
O
N
O
7a
(CDCl3)
f1 (ppm)

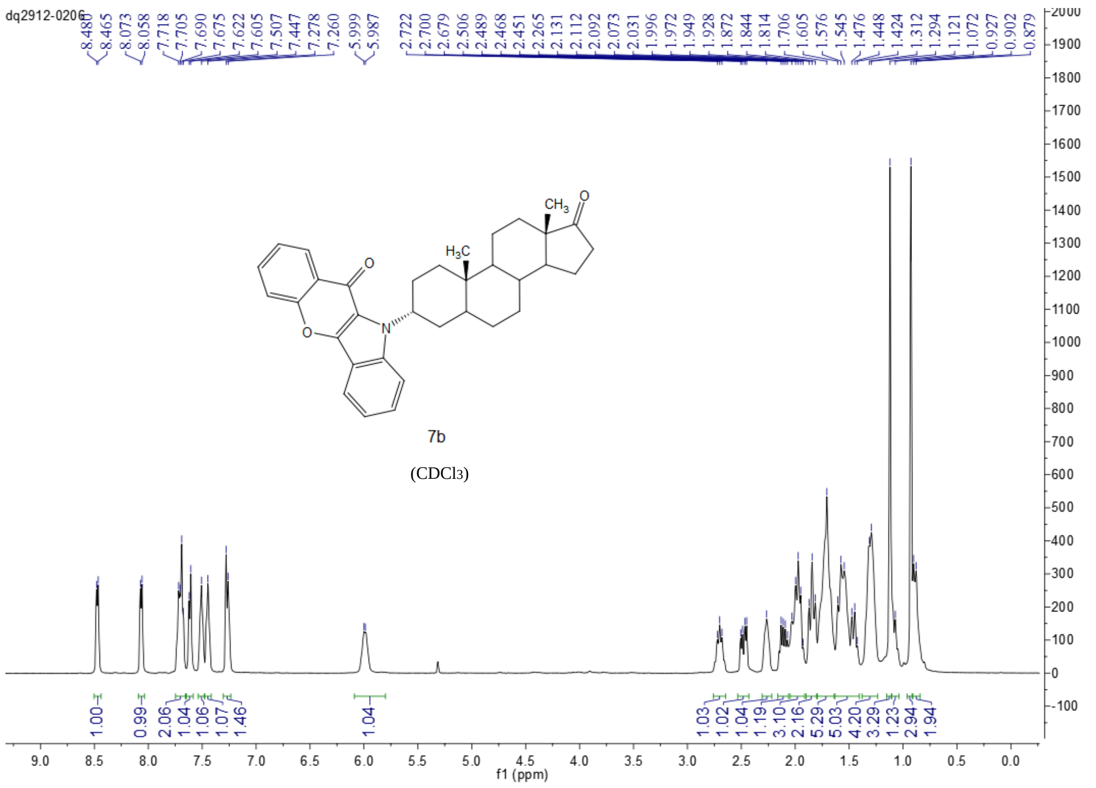
dq2912-0206
8.480
8.465
8.073
8.058
7.718
7.705
7.690
7.675
7.622
7.605
7.507
7.447
7.278
7.260
5.999
5.987
2.722
2.700
2.679
2.506
2.489
2.468
2.451
2.265
2.131
2.112
2.092
2.073
2.031
1.996
1.972
1.949
1.928
1.872
1.844
1.814
1.706
1.605
1.576
1.545
1.476
1.448
1.424
1.312
1.294
1.121
1.072
0.927
0.902
0.879
CH3
H3C
O
O
N
7b
(CDCl3)
1.00
0.99
2.06
1.04
1.06
1.07
1.46
1.04
1.03
1.02
1.04
1.19
3.10
2.16
5.29
5.03
4.20
3.29
1.23
2.94
1.94
f1 (ppm)

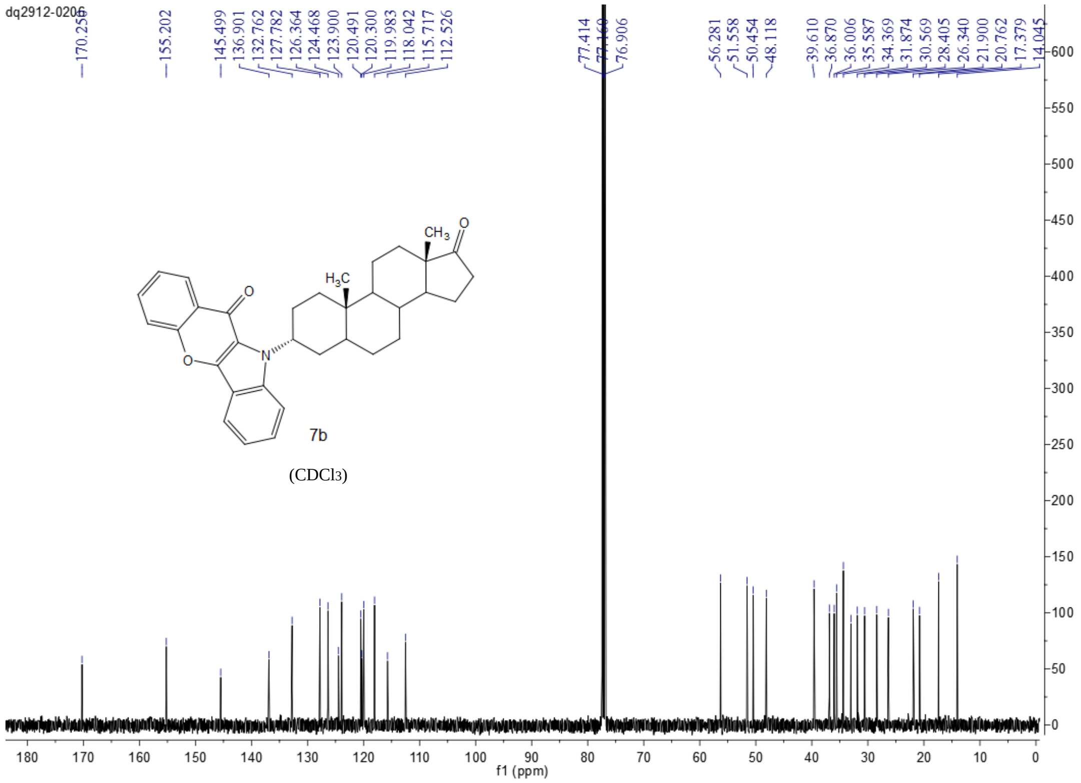
dq2912-0206
170.256
155.202
145.499
136.901
132.762
127.782
126.364
124.468
123.900
120.491
120.300
119.983
118.042
115.717
112.526
77.414
76.906
56.281
51.558
50.454
48.118
39.610
36.870
36.006
35.587
34.369
31.874
30.569
28.405
26.340
21.900
20.762
17.379
CH3
H3C
O
N
7b
(CDCl3)
f1 (ppm)

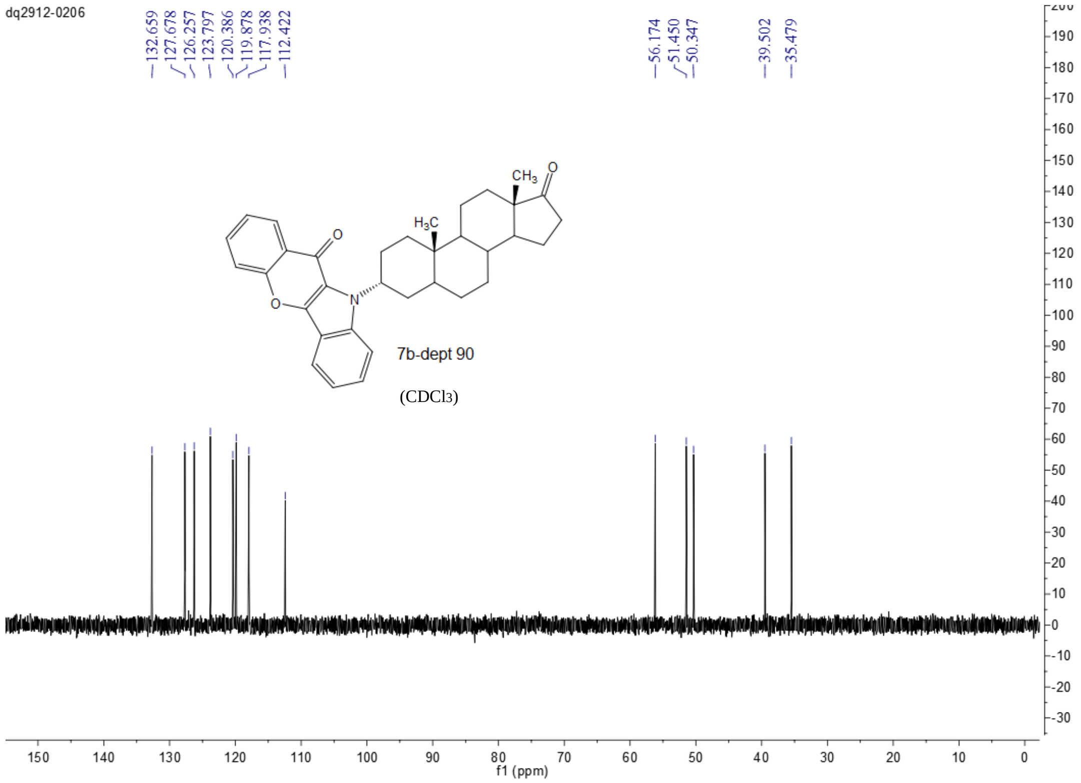
dq2912-0206
132.659
127.678
126.257
123.797
120.386
119.878
117.938
112.422
56.174
51.450
50.347
39.502
35.479
CH3
H3C
O
N
7b-dept 90
(CDCl3)
f1 (ppm)

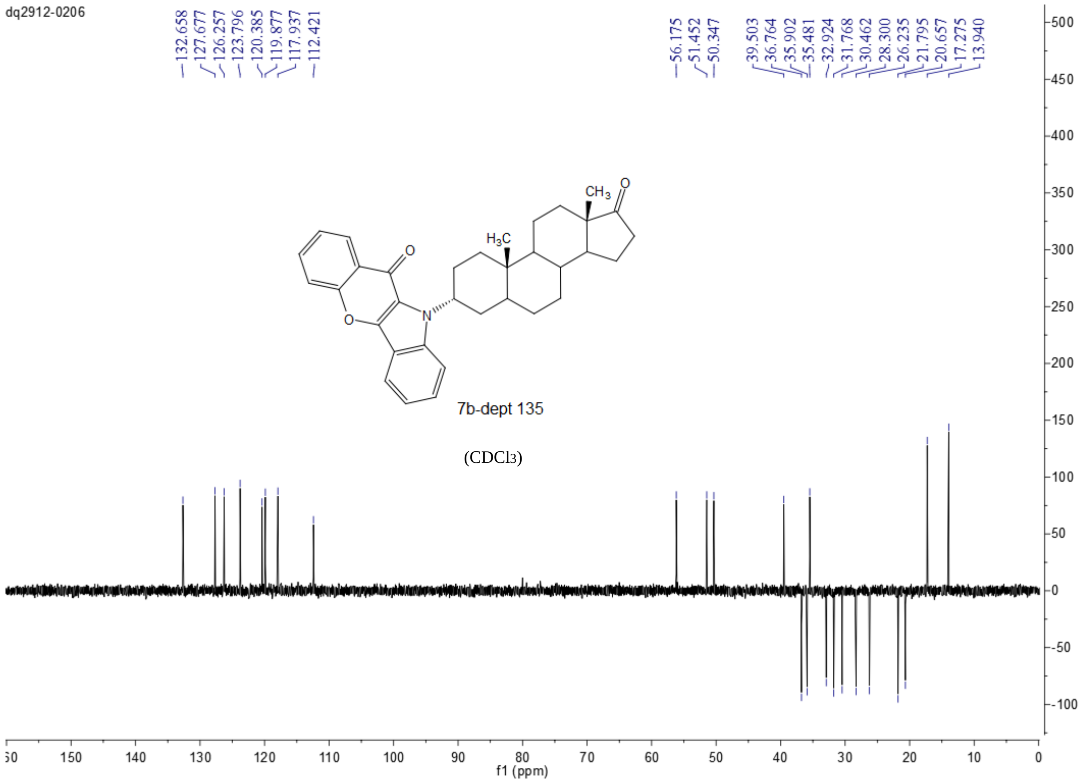
dq2912-0206
7b-dept 135
(CDCl3)
f1 (ppm)

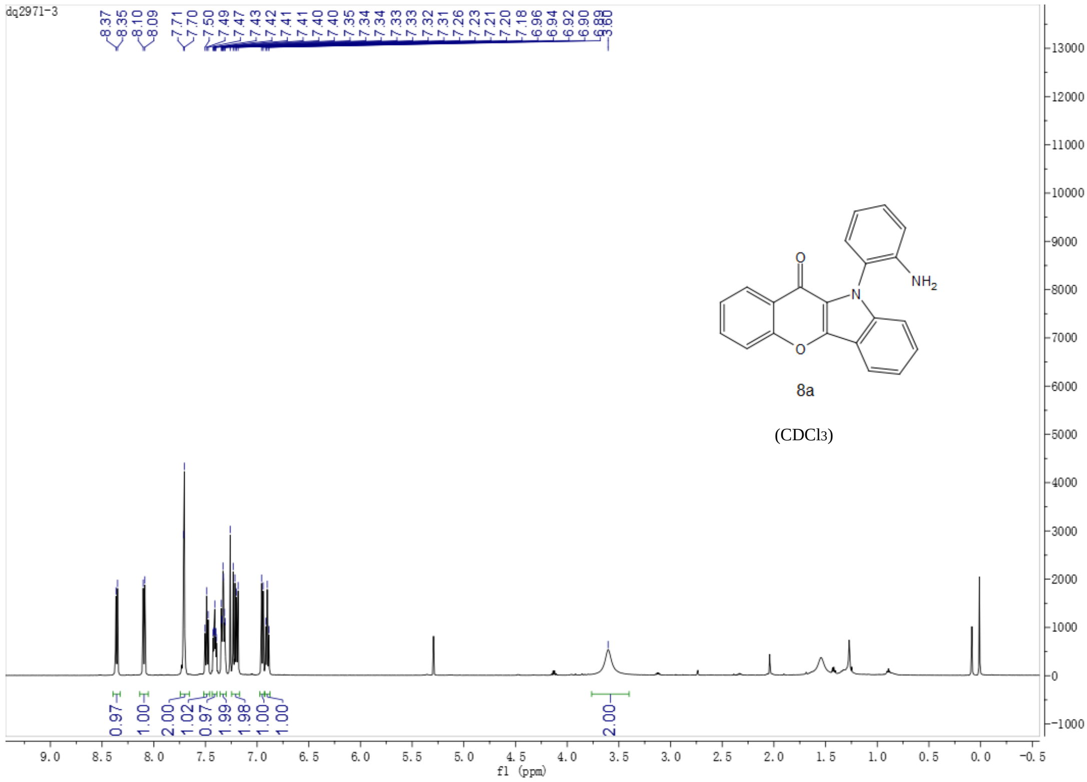
dq2971-3
8a
(CDCl3)
f1 (ppm)

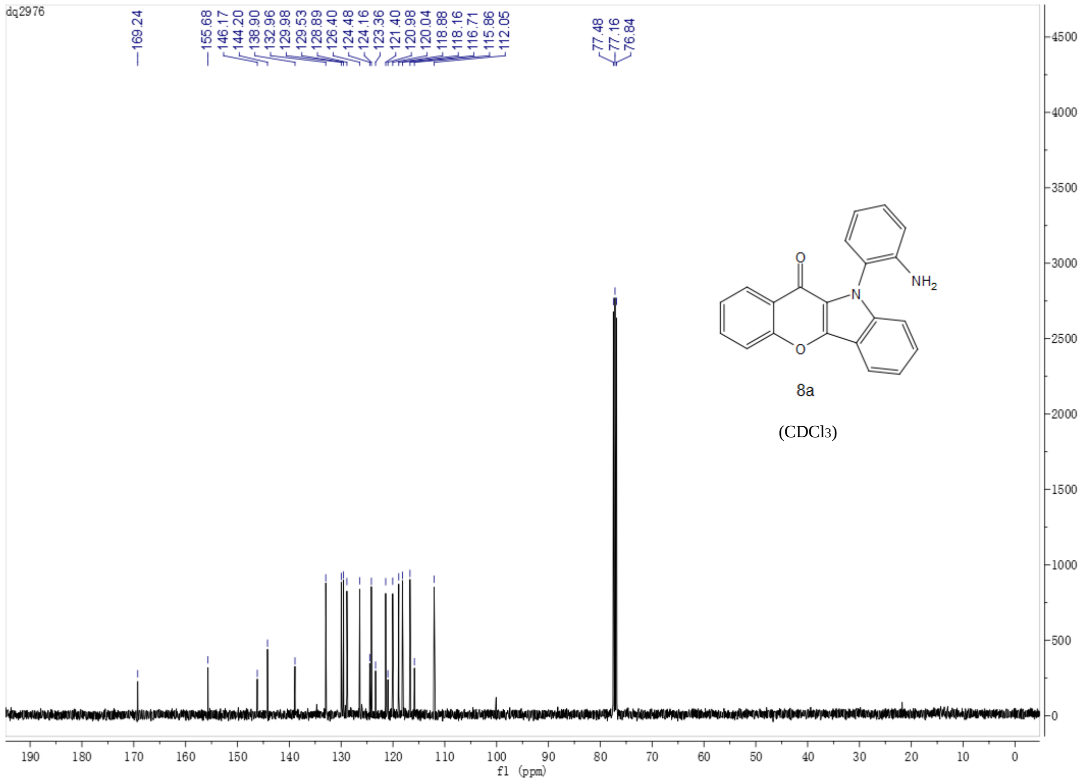
dq2976
169.24
155.68
146.17
144.20
138.90
132.96
129.98
129.53
128.89
126.40
124.48
124.16
123.36
121.40
120.98
120.04
118.88
118.16
116.71
115.86
112.05
77.48
77.16
76.84
8a
(CDCl3)
f1 (ppm)

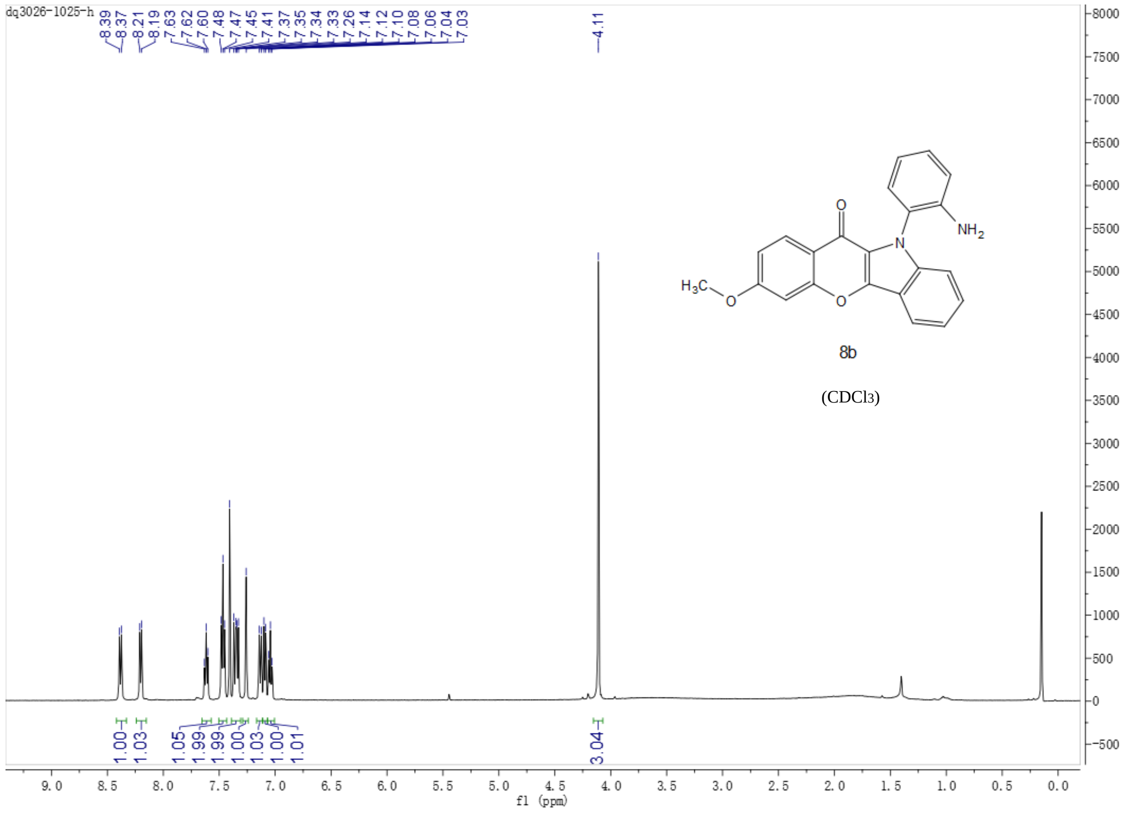
dq3026-1025-h
8.39
8.37
8.21
8.19
7.63
7.62
7.60
7.48
7.47
7.45
7.41
7.37
7.35
7.34
7.33
7.26
7.14
7.12
7.10
7.08
7.06
7.04
7.03
4.11
8b
(CDCl3)
1.00
1.03
1.05
1.99
1.99
1.00
1.03
1.00
1.01
3.04
f1 (ppm)

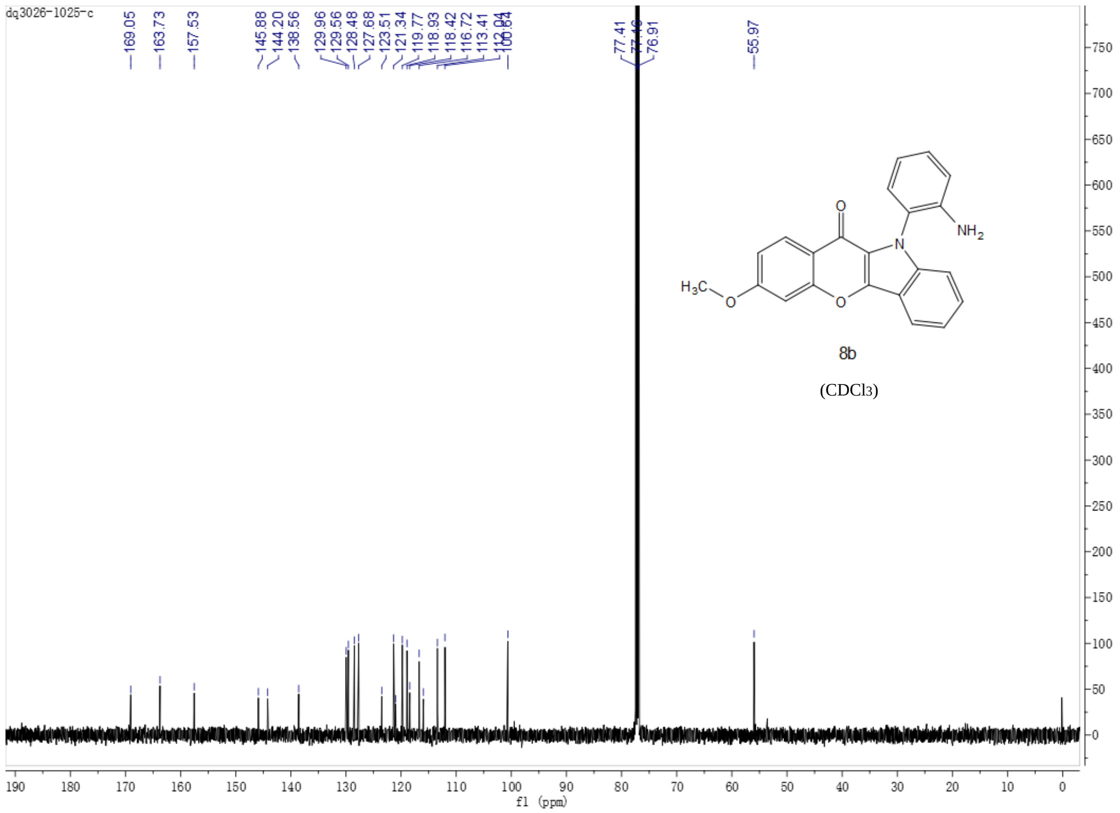
dq3026-1025-c
169.05
163.73
157.53
145.88
144.20
138.56
129.96
129.56
128.48
127.68
123.51
121.34
119.77
118.93
118.42
116.72
113.41
77.41
76.91
55.97
O
N
NH2
H3C
O
O
8b
(CDCl3)
f1 (ppm)

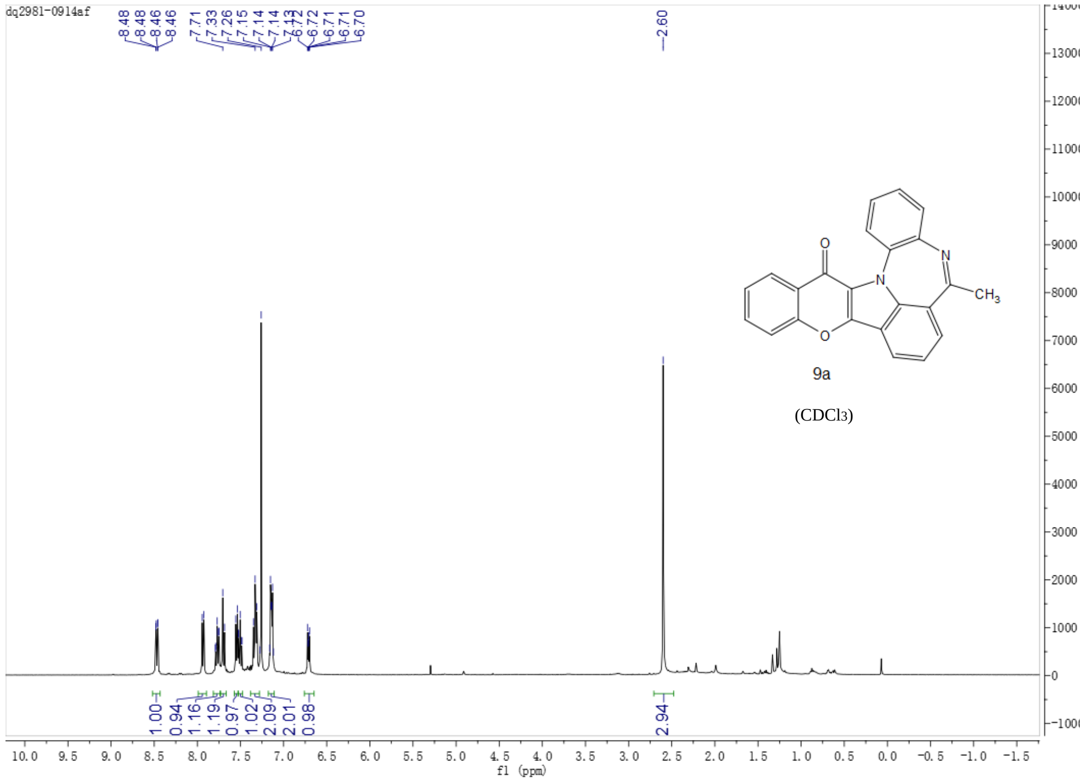
dq2981-0914af
8.48
8.48
8.46
8.46
7.71
7.33
7.26
7.15
7.14
6.72
6.71
6.71
6.70
2.60
O
N
N
CH3
O
9a
(CDCl3)
1.00
0.94
1.16
1.19
0.97
1.02
2.09
2.01
0.98
2.94
f1 (ppm)

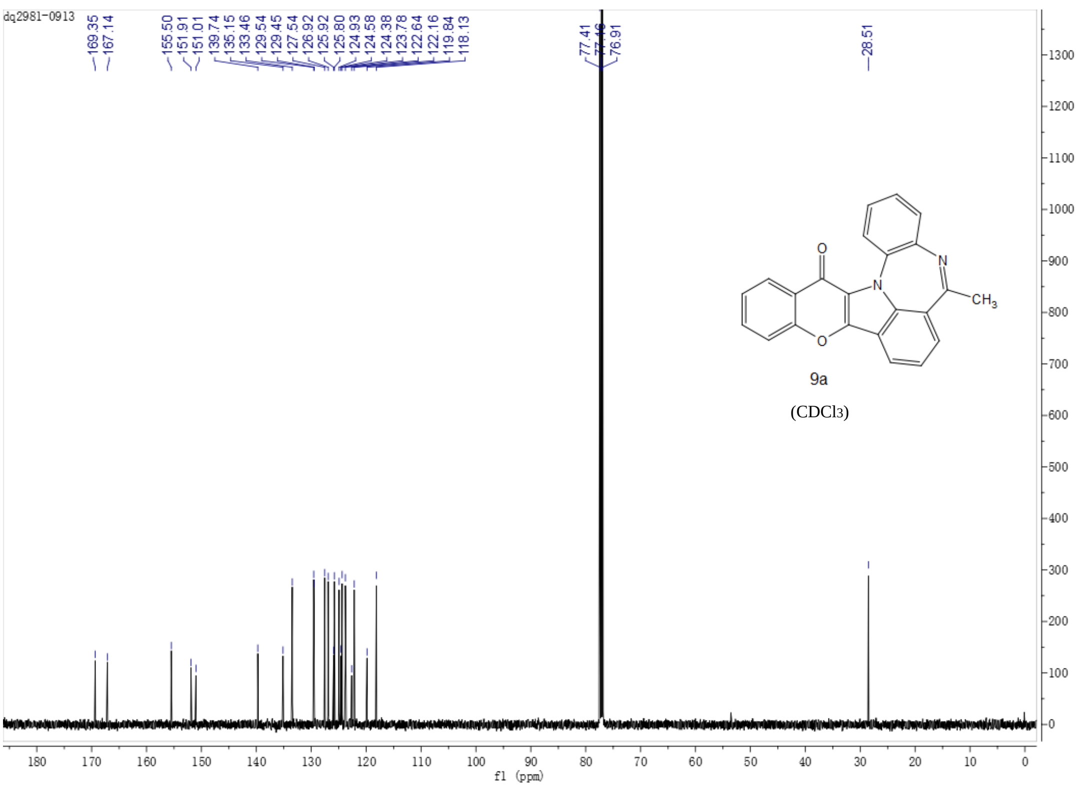
dq2981-0913
169.35
167.14
155.50
151.91
151.01
139.74
135.15
133.46
129.54
129.45
127.54
126.92
125.92
125.80
124.93
124.58
124.38
123.78
122.64
122.16
119.84
118.13
77.41
76.91
28.51
9a
(CDCl3)
f1 (ppm)

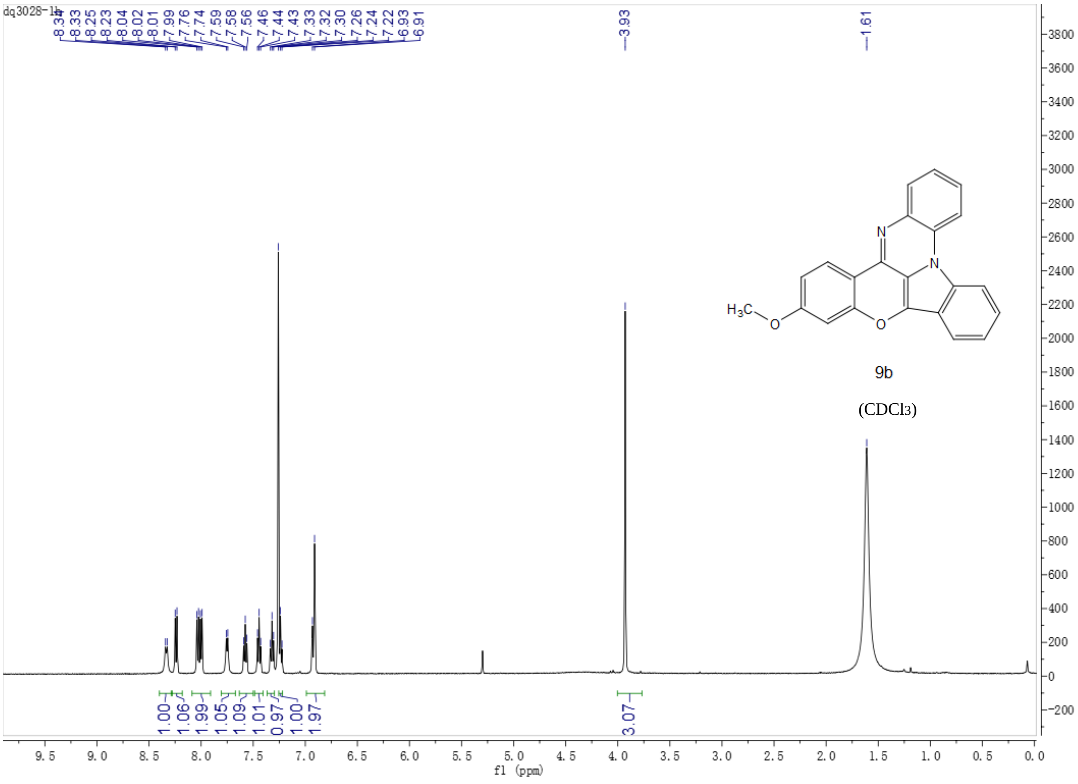
8.33
8.25
8.23
8.04
8.02
8.01
7.99
7.76
7.74
7.59
7.58
7.56
7.46
7.44
7.43
7.33
7.32
7.30
7.26
7.24
7.22
6.93
6.91
3.93
1.61
9b
(CDCl3)
1.00
1.06
1.99
1.05
1.09
1.01
0.97
1.00
1.97
3.07
f1 (ppm)

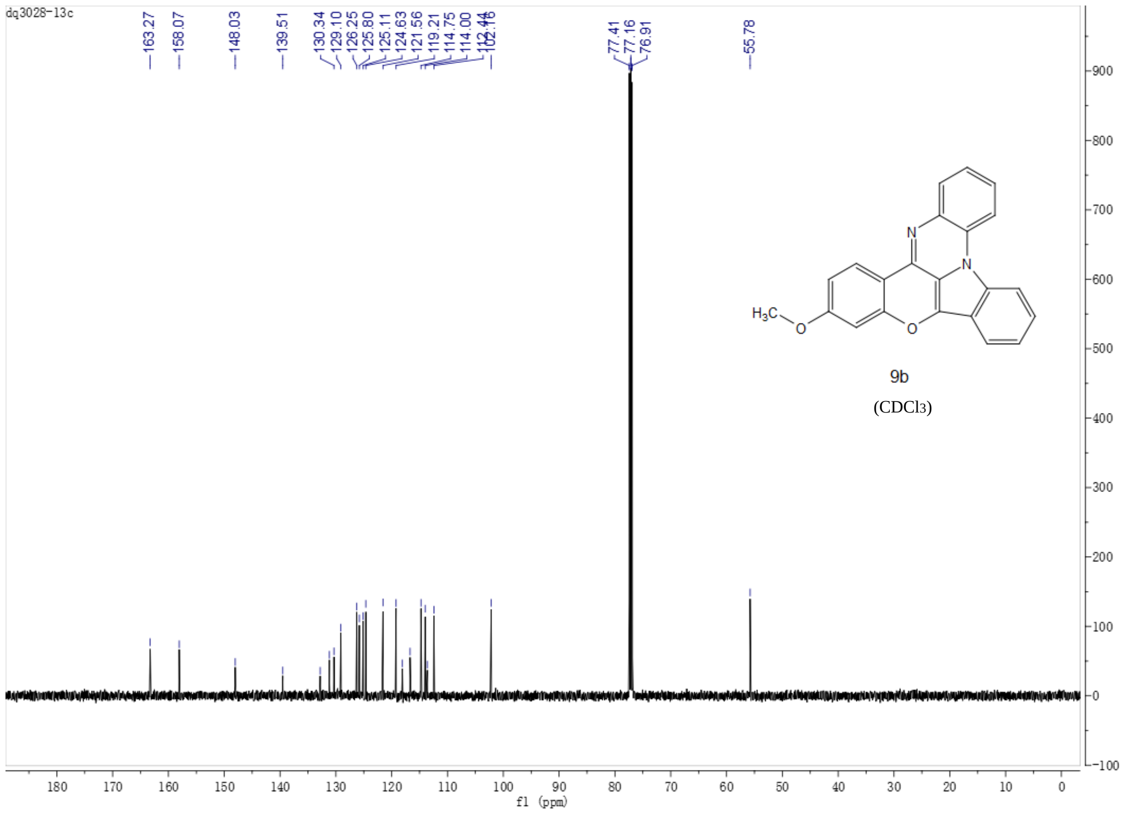
dq3028-13c
163.27
158.07
148.03
139.51
130.34
129.10
126.25
125.80
125.11
124.63
121.56
119.21
114.75
114.00
112.44
102.16
77.41
77.16
76.91
55.78
9b
(CDCl3)
f1 (ppm)
